# Transcriptome profiling of male and female *Ascaris lumbricoides* reproductive tissues

**DOI:** 10.1186/s13071-022-05602-2

**Published:** 2022-12-20

**Authors:** Orawan Phuphisut, Akkarin Poodeepiyasawat, Tippayarat Yoonuan, Dorn Watthanakulpanich, Palang Chotsiri, Onrapak Reamtong, Angela Mousley, Geoffrey N. Gobert, Poom Adisakwattana

**Affiliations:** 1 Department of Helminthology, Faculty of Tropical Medicine, Mahidol University, Bangkok, 10400 Thailand; 2 Mahidol-Oxford Tropical Medicine Research Unit, Mahidol University, Bangkok, 10400 Thailand; 3 Department of Molecular Tropical Medicine and Genetics, Faculty of Tropical Medicine, Mahidol University, Bangkok, 10400 Thailand; 4 School of Biological Sciences, Queenʼs University Belfast, Belfast, BT9 5DL UK

**Keywords:** *Ascaris lumbricoides*, Gene expression, Reproductive tissue, RNA-Seq, Transcriptome, Transcriptomics

## Abstract

**Background:**

*Ascaris lumbricoides* causes human ascariasis, the most prevalent helminth disease, infecting approximately 1 billion individuals globally. In 2019 the global disease burden was estimated to be 754,000 DALYs and resulted in 2090 deaths. In the absence of a vaccination strategy, treatment of ascariasis has relied on anthelminthic chemotherapy, but drug resistance is a concern. The propensity for reinfection is also a major challenge to disease control; female worms lay up to 200,000 eggs daily, which contaminate surrounding environments and remain viable for years, resulting in high transmission rates. Understanding the molecular mechanisms of reproductive processes, including control of egg production, spermatogenesis, oogenesis and embryogenesis, will drive the development of new drugs and/or vaccine targets for future ascariasis control.

**Methods:**

Transcriptome profiles of discrete reproductive and somatic tissue samples were generated from adult male and female worms using Illumina HiSeq with 2 × 150 bp paired-end sequencing. Male tissues included: testis germinal zone, testis part of vas deferens, seminal vesicle and somatic tissue. Female tissues included: ovary germinal zone, ovary part of the oviduct, uterus and somatic tissue. Differentially expressed genes (DEGs) were identified from the fragments per kilobases per million reads (FPKM) profiles. Hierarchical analysis was performed to identify tissue-specific genes. Furthermore, Gene Ontology (GO) and Kyoto Encyclopedia of Genes and Genomes (KEGG) analyses were employed to identify significant terms and pathways for the DEGs.

**Results:**

DEGs involved in protein phosphorylation and adhesion molecules were indicated to play a crucial role in spermatogenesis and fertilization, respectively. Those genes associated with the G-protein-coupled receptor (GPCR) signaling pathway and small GTPase-mediated signal transduction pathway play an essential role in cytoskeleton organization during oogenesis. Additionally, DEGs associated with the SMA genes and TGF-β signaling pathway are crucial in adult female embryogenesis. Some genes associated with particular biological processes and pathways that were identified in this study have been linked to defects in germline development, embryogenesis and reproductive behavior. In the enriched KEGG pathway analysis, Hippo signaling, oxytocin signaling and tight junction pathways were identified to play a role in *Ascaris* male and female reproductive systems.

**Conclusions:**

This study has provided comprehensive transcriptome profiles of discrete *A. lumbricoides* reproductive tissue samples, revealing the molecular basis of these functionally important tissues. The data generated from this study will provide fundamental knowledge on the reproductive biology of *Ascaris* and will inform future target identification for anti-ascariasis drugs and/or vaccines.

**Graphical Abstract:**

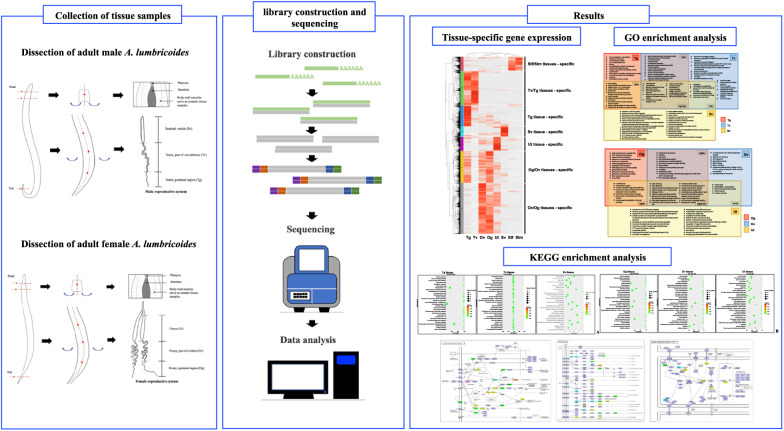

**Supplementary Information:**

The online version contains supplementary material available at 10.1186/s13071-022-05602-2.

## Background

*Ascaris lumbricoides* is a soil-transmitted helminth (STH) that cause human ascariasis, especially in tropical and subtropical regions of the world. Ascariasis has the highest prevalence of all helminthiases, infecting approximately 0.8–1.2 billion individuals worldwide, especially preschool and school-aged children [1]. The reproductive capacity of *Ascaris* drives the life cycle and exacerbates disease transmission where each individual female can produce > 200,000 fertilized eggs daily [2]. When the eggs are deposited and incubated in the soil, they develop into an infective stage (embryonated egg), which can remain viable for several years despite extreme environmental conditions that may include periods of extreme temperature fluctuations and drought; *Ascaris* eggs are also resistant to chemical treatment [3]. Control is primarily based on delivery of anthelminthic therapy to symptomatic patients or periodic mass drug administration in endemic areas. There is currently no vaccine for human ascariasis. Although benzimidazoles are effective in treating ascariasis, the development of drug resistance is a concern [4–6]. As a result, sustainable future control of ascariasis will rely on the identification of new molecular targets. Understanding the molecular mechanisms of egg production and spermatogenesis in female and male *A. lumbricoides* will contribute to the discovery of novel drug and vaccine candidates. Targeting these tissues will limit the large number of eggs produced.

Tissue-specific transcriptomics offers an opportunity to identify gene targets that underlie key functions at the tissue and cellular level. Transcriptome profiles of several helminths, generated using microarray or RNA sequencing (RNA-Seq), have targeted specific tissues [7–10], stages [11–13] and sexes [14, 15]. Indeed, transcriptome profiles of both germline and somatic tissues of the pig roundworm *Ascaris suum*, a closely related species to *A. lumbricoides*, have been generated [16]. In addition to transcriptomics, the microRNA profiles of male and female *A. suum* reproductive tissue samples have also been analyzed, focusing on the regulation of gene expression [17–19]. Despite this, there are no equivalent datasets for *A. lumbricoides*, which would enable comparative analyses of the molecular mechanisms and gene expression profiles between the two *Ascaris* species. As such, transcriptome analysis of *A. lumbricoides* reproductive tissue samples will complete the molecular basis of the genus *Ascaris*, which may aid in the establishment of effective control of both related species that pose zoonotic and anthroponotic threats [20].

The large worm size and the linear structure of the reproductive tissue in *A. lumbricoides* allows for the dissection and isolation of the discrete stages of gametogenesis in both male and female worms. This study employs RNA-Seq to generate transcriptome profiles of discrete reproductive and somatic tissue samples in adult male and female *A. lumbricoides* to identify significant differentially expressed genes (DEGs) that can be attributed to roles in egg production and spermatogenesis. Data generated in this study will contribute to better understanding of the molecular mechanisms involved in *A. lumbricoides* egg development and spermatogenesis. These insights will inform future development of target-specific ascariasis control strategies in humans, and potentially in pigs.

## Methods

### Study area

The study site was located in the Ban Mae Salid Luang village, Mae Song Sub-District, Thasongyang District and Tak Province close to the Thai-Myanmar border. This study was approved by the Human Research Ethics Committee of the Faculty of Tropical Medicine, Mahidol University, Bangkok, Thailand (MUTM 2021–020-01 and MUTM 2021–020-02).

### Stool examination

With their assent and consent, a total of 186 participants with ages ranging from 7 to 17 years were recruited to provide stool samples for the investigation of *A. lumbricoides* infection. Participants received labeled sample containers to collect their stools and send them to the study site for stool examination. Along with collecting stool samples, data such as age, gender and address were also collected.

All human stool samples were subjected to the Kato-Katz method as previously reported [21–23]. In brief, a stainless-steel sieve (size 40 mesh: 420 µm sieve opening) was used to press individual stool samples, and the non-retained material was used to fill a kit template with 39.2 mg material. The sample was covered with glycerin-malachite green-soaked cellophane and firmly pressed to disseminate the stool across the surface. After 30 min, the slide was examined under a light microscope. *Ascaris lumbricoides* eggs were recorded. All samples were anonymized.

When utilizing the Kato-Katz method, 32 of the 186 participants had *A. lumbricoides*-positive stools. *Ascaris lumbricoides*-infected participants were treated orally with a single dose of albendazole (400 mg) according to WHO guidelines [24]. The medical treatment was delivered under the physical examination and supervision of a medical doctor individually. Patients were monitored for 1 h at the study site for the appearance of any side effects from the medication, such as nausea, vomiting, headaches and dizziness. After the participant returned home, they continued to monitor any side effects on their own, and if any were found, they promptly received medical attention. No patients presented any side effects at any stage. The results of the stool examination were reported to the local health-promoting hospitals for further investigation and action on the prevention and control strategy.

### Collection of adult *A. lumbricoides* for RNA-Seq

A total of 32 individuals with *A. lumbricoides*-positive stool eggs were approached for treatment, and whole stools were collected daily for 4 days with their assent and consent. Adult *A. lumbricoides* were obtained from participants after albendazole treatment; infected individuals collected whole stool in plastic garbage bags daily for 4 days post-albendazole administration. Since worms could be expelled at different times from each participant, the stool was collected immediately after defecation and transferred to the field laboratory station within 3 h. At the field laboratory station, adult worms were recovered from the stool sample and rinsed several times with sterile 0.85% normal saline solution (NSS) to remove fecal contamination. According to the worm expulsion, each adult worm collected was examined to ensure its vitality; 15 of the 39 individual worms that were retrieved were still viable. To obtain the best quality adult worm for RNA isolation, only worms that were viable and in good physical condition were collected. Subsequently, male and female worms were distinguished morphologically under a stereomicroscope. Three adult male and three adult female worms of good quality, which were retrieved from different participants, were employed in the experiment. Male and female worms were then dissected under a stereomicroscope to retrieve their somatic and reproductive tissues. Body wall muscles at the anterior region were used as somatic tissue samples. The reproductive tissue samples were completely excised and placed in a Petri dish containing NSS. Four distinct regions with the male reproductive tissues were dissected: (i) the germinal zone of the testis (Tg), approximately 5 cm in length from the tip of the anterior testis; (ii) testis, part of vas deferens (Tv); (iii) seminal vesicle (Sv); (iv) male somatic tissue (Stm). Four distinction regions with the female reproductive tissue were dissected: (i) the germinal zone of the ovary (Og); (ii) ovary, part of the oviduct (Ov); (iii) uterus (Ut); (iv) female somatic tissue (Stf). The dissection of somatic and reproductive tissues is shown in Additional file 1: Figure S1. Note that the distinct regions within the male and female reproductive tracts, as outlined above, were identified based on a previous publication [18]. Dissected tissue samples were immediately frozen on dry ice and transported back to the main laboratory in Bangkok, Thailand, where they were stored at − 80 °C prior to RNA extraction. Six worms in total, three distinct male worms and three distinct female worms, were used in this study. Each worm was dissected for four different tissue types for examination. Therefore, RNA sequencing was performed on a total of 24 tissue samples. Each worm was taken from a different participant.

### Total RNA extraction, library construction and sequencing

Total RNA was isolated from each sample using TRIzol Reagent according to the manufacturer's instructions (Thermo Fisher Scientific, Waltham, MA, USA). The quantity and quality of total RNA was determined using an Agilent 2100 Bioanalyzer (Agilent Technologies, Palo Alto, CA, USA), NanoDrop (Thermo Fisher Scientific Inc.), and 1% agarose gel electrophoresis. One microgram of total RNA with RIN value > 6.5, which met the quality requirements of the manufacturer, was processed for library preparation and RNA sequencing by Vishuo Biomedical (Thailand) Ltd. Briefly, next-generation sequencing libraries were prepared according to the manufacturer’s protocol [NEBNext^®^ Ultra™ RNA Library Prep Kit for Illumina^®^, New England Biolabs (NEB), Ipswich, MA, USA]. Poly(A) mRNA isolation was performed using the Poly(A) mRNA Magnetic Isolation Module (NEB). mRNA fragmentation and priming were performed using First-Strand Synthesis Reaction Buffer and random primers (NEB). First-strand cDNA was synthesized using ProtoScript II Reverse Transcriptase, and second-strand cDNA was synthesized using Second-Strand Synthesis Enzyme Mix. Following this, the purified double-stranded cDNA was treated with End Prep Enzyme Mix to repair both ends and add a dA-tailing in one reaction, followed by a T-A ligation to add adaptors to both ends. Next, size selection of adaptor-ligated DNA was performed using VAHTS DNA clean beads (Vazyme Biotech Co., Ltd, Nanjing, China), and fragments of approximately 420 bp (with an approximate insert size of 300 bp) were recovered. Each sample was then amplified by PCR for 13 cycles using P5 and P7 primers (see Additional file 2: Table S1), with both primers carrying sequences that can anneal with flow cells to perform bridge PCR and the P7 primer carrying a six-base index that allows for multiplexing. The PCR products were cleaned up using VAHTS DNA clean beads, validated using Qsep 100 (Bioptic, Taiwan, China) and quantified using a Qubit 3.0 Fluorometer (Invitrogen, Carlsbad, CA, USA). Subsequently, libraries with different indices were multiplexed and loaded on an Illumina HiSeq instrument according to the manufacturer’s instructions (Illumina, San Diego, CA, USA). Sequencing was performed using a 2 × 150-bp paired-end configuration, with 6.0-Gb raw data per sample. Image analysis and base calling were conducted using the HiSeq Control Software (HCS) + OLB + GAPipeline-1.6 (Illumina).

### Bioinformatics analysis

Bcl2fastq (v.2.17.1.14) was used to process the original image data for base calling and preliminary quality analysis. The quality assessment of the sequencing data was performed using FastQC (v.0.10.1) [25]. The base quality scores, expressed in *Q* Phred. Cutadapt (v.1.9.1), were used for data filtering to remove the adapter sequences, 5′ or 3′ end bases containing N’s or quality values < 20, and reads that were < 75 bp long after trimming [26]. Subsequently, filtered data were aligned to the reference *A. lumbricoides* genome (WormBase ParaSite, BioProject PRJEB4950, Taxonomy ID 6252). Short-read alignment was performed using Hisat2 (v.2.0.1) with default parameters [27].

### Gene differential expression analysis

Read density was used to calculate the level of gene expression of all genes. Based on the read counts from HT-seq (v.0.6.1), fragments per kilobases per million reads (FPKM) were used to calculate gene expression using the formula outlined below [28]. Since three biological tissue replicates were performed, Pearson’s correlation was used to calculate the correlation of gene expression between samples to assess RNA-Seq quality. Principal component analysis (PCA) was also used to assess the correlation of gene expression between samples to reduce data complexity.

$$\mathrm{FPKM}=\frac{\mathrm{Total \ Exon \ fragments }}{(\mathrm{Mapped\ reads }\left(\mathrm{Millions}\right)\times \mathrm{Exon\ length }\left(\mathrm{kb}\right))}$$

To identify the DEGs of the discrete reproductive tissue samples, the gene expression level of all genes (FPKM profiles) of each discrete reproductive tissue was compared across the tissue samples using the Bioconductor package DESeq2 (v.1.6.3) [29]. Subsequently, significant DEGs were identified based on fold change > 2 and *Q*-value (FDR, *P*-adj) < 0.05. Normalized FPKM were hierarchically clustered to classify DEGs with similar expression patterns using Pearson’s correlation. Clustered data were graphically depicted (heatmap) using gplots in the R package.

### Gene ontology (GO) and Kyoto Encyclopedia of Genes and Genomes (KEGG) enrichment analyses

The annotation was carried out using the gene classification system, Gene Ontology (GO) database. GO annotation provides a set of dynamically updated standard vocabulary to describe the properties of genes and gene products in the organism. GO contains three ontologies that describe the molecular function, cellular component and biological process of the gene. To identify the GO terms that were enriched among DEGs against the transcriptomic background, GO enrichment analysis was performed using GOSeq (v.1.34.1) [30], based on an extension of the hypergeometric distribution with a threshold for filtering overrepresented *P*-value $\le$ 0.05.

Kyoto Encyclopedia of Genes and Genomes (KEGG) is the primary public pathway database used in this analysis [31]. Pathway enrichment analysis performed in this section is based on KEGG pathway units and used a hypergeometric test, with a threshold *Q*-value of $\le$ 0.05, to identify the pathways of the DEGs that are significantly enriched against the transcriptome background [32]. The ratio of the number of genes differentially expressed in the pathway to the total number of genes in the pathway (rich factor), *Q*-value and the number of genes enriched in the pathway were used to assess the degree of KEGG enrichment.

### Validation of gene expression by RT-qPCR

The top DEGs specific for each cluster were selected based on top three normalized expression levels within each cluster to validate the RNA-Seq results. RT-qPCR was used as follows. A DEG was selected from Tg, Sv and Ut tissue samples, which are exclusively indicated in Clusters C, D and E, respectively. If genes were expressed in groups of tissues, a DEG was selected primarily from the dominantly expressed tissue as follows: a DEG was selected primarily from Stf, Tv, Og and Ov tissue samples as predominantly expressed in the Stf/Stm tissue-specific (Cluster A), Tv/Tg—tissue-specific (Cluster B), Og/Ov—tissue-specific (Cluster F) and Ov/Og—tissue-specific (Cluster G), respectively.

All tissue samples with three biological replicates from the same samples utilized in RNA-Seq experiments were used as templates to perform RT-qPCR validation assays for each representative differentially expressed tissue-specific gene. In summary for RT-qPCR, initially, first-strand cDNA was synthesized as follows; total RNA (1 μg) from each tissue sample was treated with 1 U of DNase I (Thermo Fisher Scientific) before synthesizing first-strand cDNA using a RevertAid First-Strand cDNA Synthesis kit (Thermo Fisher Scientific) according to the manufacturer’s instructions. Each 20 μL reaction mixture contained 1 µg of total RNA, 1 mM of each dNTP and 10 μM Oligo (dT) primer (Sigma-Aldrich, Inc., Saint Louis, MO). The reaction mixture was chilled on ice for 1 min after being incubated at 65 °C for 5 min. The reaction mixture was mixed with 4 μl of 5 × RT buffer and 1 μl of Revert Aid RT, and it was then incubated at 42 °C for 1 h. Afterwards, the reaction mixture was incubated for 5 min at 70 °C. Second, first-strand cDNA was utilized as the template for the SYBR Green RT-qPCR. Each 20 μl reaction mixture contained 2 μl of first-strand cDNA, 1 × iTaq Universal SYBR Green Supermix (Bio-Rad Laboratories, Philadelphia, PA, USA) and 300 nM of each forward and reverse primer. Lastly, amplification was carried out using a CFX96 Real-Time PCR System (Bio-Rad Laboratories, Hercules, CA, USA) according to the following protocol: pre-incubation at 95 °C for 5 min, followed by 40 cycles of 95 °C for 20 s and 60 °C for 1 min. Melting curve analysis was performed from 65 °C to 95 °C.

Eukaryotic translation initiation factor 6 (eIF6) and NADH cytochrome b5 reductase were used as internal controls to normalize gene expression levels [2, 7]. The relative gene expression levels were calculated using the 2^−∆∆Ct^ method [33, 34]. RT-qPCR experiments were performed in triplicate. Primer sequences for each target gene were designed using Primer3Plus (http://www.bioinformatics.nl/cgi-bin/primer3plus/primer3plus.cgi) with default parameters (see Additional file 2: Table S1). Prior to further investigation, each primer pair was verified for specificity by blasting it against the *A. lumbricodes* transcriptome database to identify any non-target transcripts (https://parasite.wormbase.org/Ascaris_lumbricoides_prjeb4950/Info/Index/).

## Results and discussion

### RNA sequencing data and quality assessment of discrete *A. lumbricoides* reproductive tissues

After quality assessment and trimming, sequences ranging from 40 to 50 million pair-end 150-bp reads were generated from biological replicates of each tissue sample. As a result, pooled technical replicates of each tissue sample yielded over 120 million reads, of which more than 98.0% of total raw reads passed the quality filter. Furthermore, approximately 97% and 92% of total nucleotide bases had Phred quality scores > 20 and 30, respectively, indicating high read quality; GC content in the clean reads ranged from 46 to 49% (Additional file 3: Table S2).

More than 75% of all clean reads from almost all tissue samples were successfully mapped to the *A. lumbricoides* reference genome, indicating that no contamination had occurred and the appropriate reference genome was selected. Note that one replicate of *A. lumbricoides* Ut tissue sample generated a clean read that mapped to the reference genome at a lower rate than the other tissues and/or replicates, at approximately 65%, which could be explained by variation in tissue samples. Consistent variation in gene expression profiles among tissue samples would require further investigation, such as analyzing more samples from the same tissues/regions within a tissue and/or generating additional reference genomes. The mappable reads, which ranged from 58 to 81% of the total clean reads, were uniquely mapped to one location within the *A. lumbricoides* reference genome, whereas the remaining reads were mapped to multiple locations within the genome (see Additional file 4: Table S3 for raw, filtered and alignment data statistics). Further analysis of the distribution of the mappable reads within the reference genome revealed that > 80% of reads were mapped to the exon region in all tissue samples whereas the remaining reads were distributed in the intergenic and intron regions (Additional file 5: Figure S2).

**Fig. 1:**
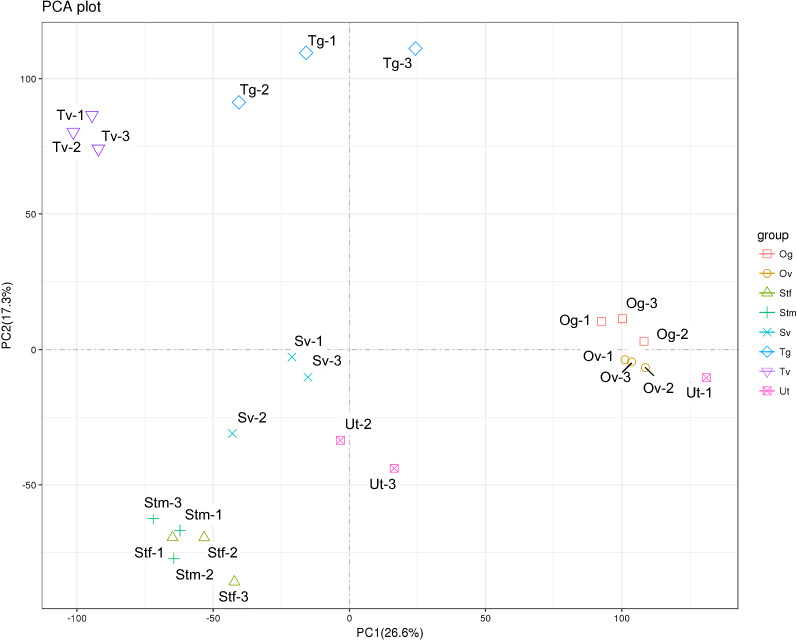
Principal component analysis (PCA) showed congruence within the replicates of the different samples (replicates for each sample are indicated by the same color), except one sample of Ut tissue. Male tissue samples include Tg: the germinal zone of the testis; Tv: testis, part of vas deferens; Sv: seminal vesicle; Stm: male somatic tissues. Female tissue samples include Og: the germinal zone of the ovary; Ov: ovary, part of oviduct; Ut: uterus; Stf: female somatic tissues. Three biological replicates of each male and female tissue type were analyzed in this study, with each replicate from a worm derived from a different participant

Pearson’s correlation analysis was used to assess RNA-Seq quality (Additional file 6: Figure S3). The results indicate that most tissue samples displayed reproducibility and consistent quality across biological replicates, but Ut tissue samples had the lowest congruence within replicates, which, as highlighted above, could be due to the tissue-specific variation within samples. On the basis of the PCA, the sample was divided into six groups, including (i) Tg tissue, (ii) Tv tissue, (iii) Sv tissue, (iv) two replicates of Ut tissue, (v) Og, Ov and one replicate of Ut tissue and (vi) Stf and Stm tissue samples (Fig. 1). There was clearly a distinct grouping for male reproductive tissue samples. However, Og and Ov appeared to be grouped together for female reproductive tissue samples, with one replicate of Ut tissue merging in this group, suggesting that they were closely related to the Og and Ov tissue samples. This might be due to an immature female adult used in this study, in which the reproductive organs, especially the Ut tissue, had not fully developed. In addition, the somatic tissue samples of the male and female worms were grouped together, demonstrating a high level of correlation between the transcriptome of the samples (see Additional file 6: Figure S3 for data on correlation between samples).

However, the limitations based on the acquisition of clinical material do not make this surprising. Particularly the application of anthelmintic treatment of individuals and the clearance of adult worms limited the quality of isolated RNA. We highlight that the value of our unique clinical data and samples needs to be considered in the context of our human ethical requirements.

### Determination of DEGs across the discrete tissue samples

**Fig. 2:**
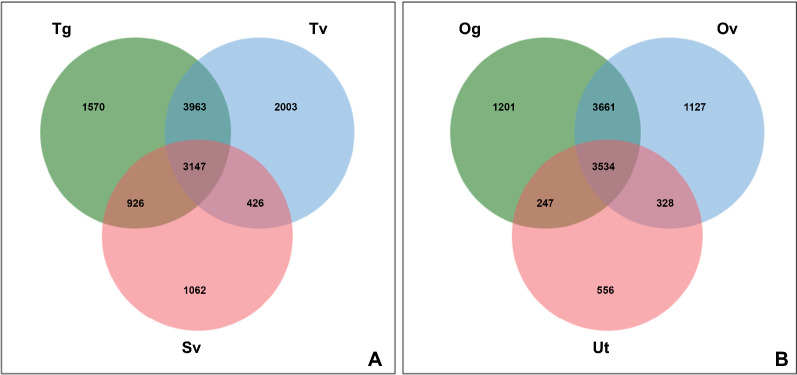
Venn diagram demonstrating the number of DEGs in each tissue type and degree of overlapping in expression of those genes among the male (A) and female (B) reproductive tissues. Male tissue samples include Tg: the germinal zone of the testis; Tv: testis, part of vas deferens; Sv: seminal vesicle. Female tissue samples include Og: the germinal zone of the ovary; Ov: ovary, part of oviduct; Ut: uterus. The complete list of DEGs is shown in Additional file 7: Table S4

According to a Venn diagram, analysis of expression indicated overlap in expression (3147 DEGs) among three male reproductive tissues (Fig. 2). Genes associated with protein phosphorylation, such as protein kinase domain-containing proteins and serine/threonine-protein phosphatases, had overlapped expression in male reproductive tissues. In two tissue comparisons, 3963 (Tg and Tv), 926 (Tg and Sv) and 426 DEGs (Tv and Sv) had expression overlap, while only Tg, Tv and Sv tissues were discovered to particularly express 1570, 2003 and 1062 DEGs, respectively. All female reproductive tissues showed overlapping expression of 3534 DEGs. There were 3661 (Og and Ov), 247 (Og and Ut) and 328 DEGs (Ov and Ut) that had expression overlap in the two tissue comparisons. It was discovered that only Og, Ov and Ut tissues specifically expressed 1201, 1127 and 556 DEGs, respectively. The complete list of DEGs is shown in Additional file 7: Table S4. It is interesting to note that DEGs associated with vesicle-mediated transport, such as the protein transport protein SEC23, were discovered to be particularly expressed in the Tv tissue. This protein is the core component of the coat protein complex II (COPII) and functions to transport newly synthesized proteins and lipids from the endoplasmic reticulum (ER) to the Golgi apparatus in cells for secretion (anterograde transport) [35]. Moreover, DEGs (ALUE 0000599901) associated with COPI (retrograde transport) were discovered to be expressed in the Tv tissue, indicating that this tissue participates in both anterograde and retrograde transport. However, Sv tissue only displayed retrograde transport activity since only the expressed DEGs (ALUE 0000977901 and ALUE 0001963101) were linked to COPI. According to a report, COPI and COPII are both involved in transport of vesicles during acrosome development [36], suggesting they may be important for *Ascaris* spermatogenesis.

### Tissue-specific gene expression patterns

**Fig. 3:**
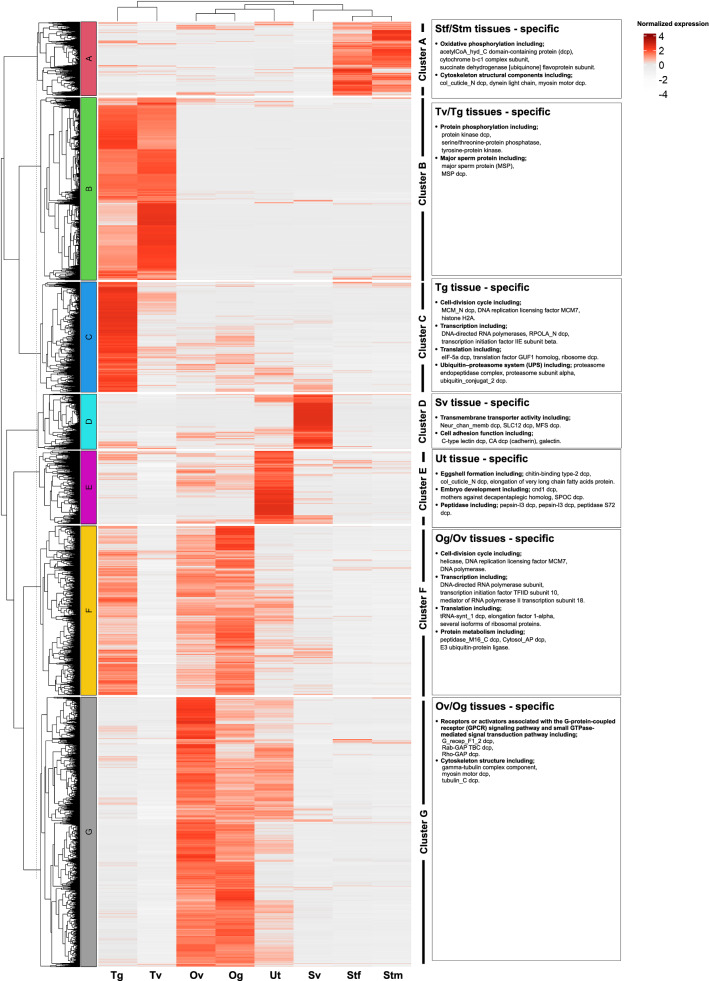
Hierarchical clustering of *Ascaris lumbricoides* transcriptome profiles to discrete tissue samples. Male tissue samples include Tg: the germinal zone of the testis; Tv: testis, part of vas deferens; Sv: seminal vesicle; Stm: male somatic tissue. Female tissue samples include Og: the germinal zone of the ovary; Ov: ovary, part of oviduct; Ut: uterus; Stf: female somatic tissue. Data from three biological replicates were combined prior to clustering. Only a few DEG examples were presented in the right box, which revealed DEGs preferentially expressed in particular tissues. Tissue-specific clusters are labeled A–G on the left and were used for subsequent tissue-specific functional analysis. The number indicates the normalized expression and ranges from white (no expression) to red (extremely high expression). Additional file 8: Table S5 shows a list of all genes, but only those that are expressed in at least one tissue

To identify genes preferentially transcribed in specific tissues, hierarchical clustering was performed using the FPKM values of all *A. lumbricoides* protein-coding genes expressed in the eight tissue samples analyzed. The hierarchical clustering result revealed that 10,157 DEGs were identified and classified into seven clusters (A–G) (Fig. 3), of which DEGs were most classified in Cluster G (2935 DEGs), followed by Clusters B (1988 DEGs), F (1844 DEGs), C (1199 DEGs), A (800 DEGs), E (792 DEGs) and D (599 DEGs). Clusters C, D and E demonstrated unique tissue specificity and were specifically expressed in Tg, Sv and Ut, respectively, whereas Clusters A, B, F and G were predominantly expressed in more than one tissue and were observed in Stf/Stm, Tv/Tg, Og/Ov and Ov/Og, respectively. Based on the numbers of DEGs distributed in specific tissue, > 50% of the total DEGs were predominantly expressed in female reproductive tissue samples (5571 genes, 54.85%), followed by the male reproductive tissue samples (3786 genes, 37.27%) and male and female somatic tissue samples (800 genes, 7.88%) (see Additional file 8: Table S5 for all genes expressed in at least one tissue).

#### DEGs that are predominantly expressed in Stm and Stf tissue samples (Cluster A)

Cluster A yielded results that were specific to Stm and Stf tissue samples. It was expected that expressed genes associated with oxidative phosphorylation would be predominant in somatic tissue. These included acetylCoA_hyd_C domain-containing protein, cytochrome b-c1 complex subunit, cytochrome b561 domain-containing protein, cytochrome c domain-containing protein, cytochrome c oxidase subunit 1, cytochrome c oxidase subunit 3 and succinate dehydrogenase [ubiquinone] flavoprotein subunit and indicated that energy production was the primary activity in this tissue. Additionally, several genes encoding cytoskeleton structural components (Fig. 3 and Additional file 8: Table S5) were also dominantly expressed in this tissue. This was expected since the cuticle and musculature are major structural components of the somatic tissue. These findings align with the active movement of the worm in the intestine to protect itself from the weep-and-sweep response in the expulsion mechanism [37]. In addition to tubulin, the target for benzimidazole drugs, other candidates involved in movement and energy generation should be explored in the future for innovative anthelminthic chemotherapy.

#### DEGs that are predominantly expressed in Tv and Tg tissue samples (Cluster B)

Cluster B was highly expressed in Tv and Tg tissue samples but was more predominant in Tv. The enriched genes are associated with protein phosphorylation, including protein kinase domain-containing protein, serine/threonine-protein phosphatase and tyrosine-protein kinase, all of which encode protein kinases which are pivotal regulators of cellular function [38]. When activated, these signaling enzymes phosphorylate transcription factors and other intracellular proteins, leading to alteration in gene expression or other cellular behavior and activities [38]. Moreover, protein kinases can drive multiple important functions in addition to influencing one another through cross-talk [39]. The role of a serine/threonine kinase member, vaccinia-related kinase, in *Caenorhabditis elegans* has been investigated where it was found to be localized in the germline and early embryos. Mutation of *C. elegans* vaccinia-related kinase resulted in defects in germline, uterine and vulval development [40, 41]. Consistent with our finding in *A. lumbricoides*, the genes encoding serine/threonine kinase and phosphatase were overrepresented among the sperm-enriched genes in *C. elegans*, which suggests an important function during spermatogenesis [2]. In *Schistosoma*, inhibitions of several protein kinases, e.g., Syk kinase, Src kinase and polo-like kinases, impaired spermatogenesis and oogenesis, which have also been proposed as a potential target for novel therapy [38, 42–44]. Several genes encoding serine/threonine and tyrosine kinases were found in spermatogenesis-enriched genes in a study on *A. suum* germline transcriptomes [2], which aligns with data presented here. These potential targets in *A. suum* and *A. lumbricoides* should be immediately selected and thoroughly characterized for the development of anthelminthic drugs. Aside from protein kinases, the genes encoding major sperm protein (MSP) (Fig. 3 and Additional file 8: Table S5) were highly expressed in this cluster; these are structural molecules that have been reported to play a role in the motility of amoeboid sperm by the regulated assembly and disassembly of MSP [45, 46].

#### DEGs that are predominantly expressed in Tg tissue sample (Cluster C)

Genes in Cluster C were specifically expressed in Tg tissue, which are involved in the cell-division cycle, including MCM_N domain-containing protein, DNA replication licensing factor MCM7, histone H2A, histone H2B, histone H3, histone H4 and dynein light chain and myosin_tail_1 domain-containing protein (Fig. 3 and Additional file 8: Table S5). The strong expression of these genes might be due to pronuclear migration and spindle assembly in the cell division cycle. Furthermore, a set of genes associated with transcription, namely, DNA-directed RNA polymerases, RPOLA_N domain-containing protein, F-box domain-containing protein, cleavage and polyadenylation specificity factor subunit 2, U2 snRNP auxiliary factor large subunit, transcription initiation factor IIE subunit beta and C2H2-type domain-containing protein, and a set of genes associated with translation, namely eIF-5a domain-containing protein, translation factor GUF1 homolog and several isoforms of ribosome domain-containing protein, were predominantly expressed. The overexpression of these genes suggests that protein synthesis is particularly active in this tissue. Furthermore, other genes of interest, including several isoforms of proteasome (proteasome endopeptidase complex, proteasome subunit alpha and proteasome subunit beta as well as ubiquitin_conjugat_2 domain-containing protein and E3 ubiquitin-protein ligase) were also highly expressed in Tg. The ubiquitin-proteasome system (UPS) is one of the most important proteolytic systems for protein degradation and is associated with numerous dynamic cellular processes [47]. UPS maintains protein homeostasis in the male reproductive system to regulate the progression of spermatogenesis at various levels [48]. Proteasome and ubiquitin have been proposed as embryonic lethal genes in *C. elegans* because of the demonstration that their inhibition causes cell division arrest to the embryo [49]. Inhibition of proteasomes has been reported to shorten the longevity of *C. elegans* [50]. In this regard, UPS may also be essential for spermatogenesis and *Ascaris* viability.

#### DEGs that are predominantly expressed in Sv tissue sample (Cluster D)

Several genes associated with transmembrane transporter activity were highly expressed in Sv tissue (Cluster D). Moreover, the group of genes with cell adhesion function, including C-type lectin domain-containing protein, CA domain-containing protein (cadherin), galectin and VWFA domain-containing protein, was also highly expressed in Sv tissue. Adhesion molecules play a crucial role in the early reproduction event, including gamete transport, fertilization, embryonic development and implantation [51]. The function of these genes is predominantly required for gamete fusion. As expected, the high expression of adhesion molecules in this tissue may indicate that sperm are ready for fertilization [52]. In addition to Sv tissue, adhesion molecules and metalloproteases (zinc metalloproteinases) were also detected in Ut tissue to again facilitate gamete fusion. High expressions of calponin-homology domain-containing protein, profilin and tubulin alpha chain were also observed in Sv, indicating that the cytoskeleton supports amoeboid sperm movement [46].

#### DEGs that are predominantly expressed in Ut tissue sample (Cluster E)

Interrogation of Cluster E revealed that genes predominantly expressed in Ut tissue were primarily focused on eggshell formation, including chitin-binding type-2 domain-containing protein, col_cuticle_N domain-containing protein and elongation of very long chain fatty acids protein (Fig. 3 and Additional file 8: Table S5). While embryo development may be supported by the expression of the genes including cnd1 domain-containing protein, mothers against decapentaplegic homolog, SPOC domain-containing protein and SUEL-type lectin domain-containing protein. Interestingly, mothers against decapentaplegic (Mad) homolog was discovered in this study. Mad is a vital gene in *Drosophila melanogaster*, which contributes to the development of the early embryo and 15 imaginal discs. Its mutation can cause embryonic dorsal-ventral patterns and adult appendage defects [53–55]. The Mad homolog, named SMA gene, was discovered in *C. elegans*, and its alteration affects body size and male tail development. The SMA gene encodes the signaling components of the transforming growth factor-beta (TGF-β) signaling pathway, such as sma-2, sma-3 and sma-4. The TGF-β signaling pathway contributes to body size and dauer formation in *C. elegans* and is involved in innate immunity, mesoderm and ectoderm patterning, longevity and fat metabolism [56]. Additionally, it was discovered that a component of the TGF-β signaling pathway is localized in reproductive tissue samples [57] and plays a role in embryogenesis in *Haemonchus contortus* [58]. The expression of a signaling component in this tissue was associated with the TGF signaling pathway, implying that this route is crucial in *Ascaris* embryogenesis.

Several isoforms of genes encoding peptidases, including pepsin-I3 domain-containing protein, pepsin-I3 domain-containing protein, peptidase S72 domain-containing protein, peptidase_M13 domain-containing protein and peptidase_M28 domain-containing protein, were expressed in Ut tissue. It has been reported in *C. elegans* that peptidases are actively expressed during embryogenesis and at the first stage of larval development because of tissue remodeling and the degradation of the nematode cuticle [59]. Since peptidases have been proposed as therapeutic and vaccine targets, our discovery provides a novel target that potentially contributes to embryogenesis and could be employed as a future target for effective control.

#### DEGs that are predominantly expressed in Og and Ov tissue samples (Cluster F)

In Cluster F (Og and Ov-specific), genes associated with the cell-division cycle, including helicase, histone H2A, histone H3, DNA replication licensing factor MCM7, DNA polymerase, Rad51 domain-containing protein, RECA_2 domain-containing protein and SMC_N domain-containing protein, were predominantly expressed in Og tissue. Furthermore, upregulation of genes involved in transcription and translation was observed in this study (Fig. 3 and Additional file 8: Table S5). In addition to the groups of genes mentioned above, the Og tissue highly expressed several genes associated with protein metabolism (Additional file 8: Table S5).

#### DEGs that are predominantly expressed in Ov and Og tissue samples (Cluster G)

Genes encoding receptors or activators associated with the G-protein-coupled receptor (GPCR) signaling pathway and small GTPase-mediated signal transduction pathway were discovered to be expressed preferentially in Cluster G (Ov and Og specific), including G_recep_F1_2 domain-containing protein, G-protein gamma domain-containing protein, Rab-GAP TBC domain-containing protein and Rho-GAP domain-containing protein. Both signaling pathways are associated with cytoskeletal activity [60, 61]. Rho-GAP (Rho GTPase) domain-containing proteins act as molecular switches, transducing signals by switching between an inactive and active GTP-bound state. Signal transductions through these pathways regulate a range of diverse cellular functions, including actin cytoskeleton rearrangement, regulation of gene transcription, cell cycle regulation, the control of apoptosis and membrane trafficking [62, 63]. The Rho GTPase family has been reported to play a crucial role in cytoskeleton organization, and our study discovered that a collection of genes associated with cytoskeleton structure was expressed in Ov and Og (Fig. 3 and Additional file 8: Table S5). The cellular cytoskeleton undergoes significant changes during oogenesis due to cell migration, membrane fusion and cytoskeleton remodeling [64], and our results showed that Rho GTPase may provide a vital function in these tissues examined. Rho-1 inhibition in *C. elegans* caused early embryonic arrest and cytokinesis failure, indicating the importance of Rho signaling at the earliest stages of development [65]. Rho GTPase inactivation has been reported to cause embryonic mortality in *Drosophila* and mice [66]. In this regard, Rho GTPase may be a promising target for *Ascaris* drug development.

### Differential gene GO enrichment analysis

#### The enriched GO Terms of the male reproductive tissue samples

**Fig. 4:**
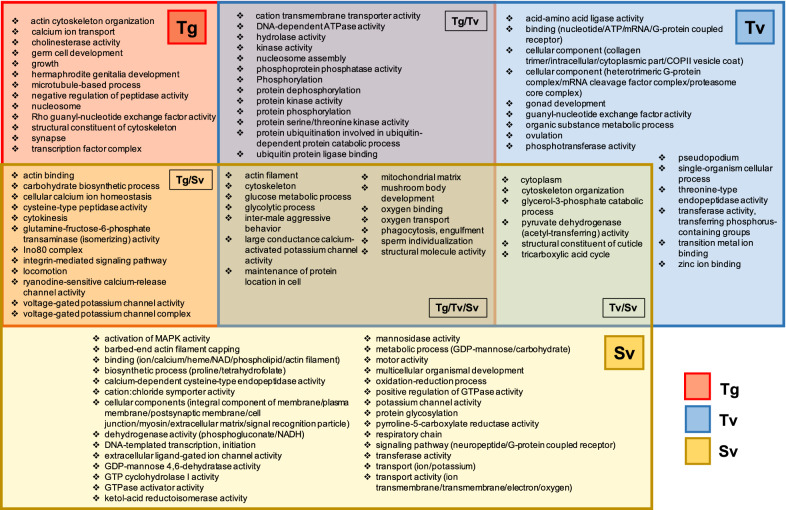
GO Term significantly enriched (*P*-value < 0.05) in the male discrete reproductive tissues. Red, blue and yellow boxes represent Tg (the germinal zone of the testis), Tv (testis, part of vas deferens) and Sv (seminal vesicle) tissues, respectively. Overlapping colored boxes represent GO Terms enriched between tissue samples. Due to the large number of GO Terms enriched within each tissue sample, groupings under general terms were used. The complete list of tissue-enriched GO Terms is shown in Additional file 9: Table S6

On the basis of the enriched GO Terms of the male reproductive tissue samples, 52, 56 and 83 enriched GO Terms were identified for Tg, Tv and Sv tissue samples, respectively, relative to the male somatic tissue. Owing to the large number of enriched GO Terms, groupings were performed under general terms (Fig. 4). Additional file 9: Table S6 contains the complete list of enriched GO Terms for male reproductive tissue. On the basis of DEGs under GO Terms, the top three GO Terms for Tg tissue identified were protein phosphorylation (88 DEGs), phosphorylation (78 DEGs) and protein kinase activity (72 DEGs); those for Tv tissue were ATP binding (224 DEGs), nucleotide binding (141 DEGs) and protein phosphorylation (106 DEGs); those for Sv tissue were an integral component of membrane (122 DEGs), membrane (83 DEGs) and cytoplasm (61 DEGs). When analyzing the top GO Terms based upon statistical significance (*P*-value), the three most significant GO Terms for Tg were protein kinase activity, phosphoprotein phosphatase activity and phosphorylation; those for Tv were protein phosphorylation, protein kinase activity and protein serine/threonine kinase activity; those for Sv were cytoskeleton, an integral component of membrane and actin binding.

According to the Go Term findings, both Tg and Tv tissues presented a set of genes related to protein phosphorylation, which included a range of activities: kinase, phosphoprotein phosphatase, phosphorylation, protein dephosphorylation, protein kinase activity, protein phosphorylation and protein serine/threonine kinase activity. These findings corroborated the DEG hierarchical clustering, which highlighted that protein phosphorylation, especially kinase activity, is significantly expressed in Tg and Tv tissue samples, which is likely to be essential for spermatogenesis.

The majority of the GO Terms specific to Sv tissue were associated with carbohydrate metabolism and respiratory chain reactions. Genes associated with these functions included phosphogluconate dehydrogenase activity, NADH dehydrogenase activity, GDP-mannose 4,6-dehydratase activity, mannosidase activity, GDP-mannose metabolic process, carbohydrate metabolic process, oxidation-reduction process and respiratory chain, implying a requirement of strong energy output promotes sperm movement within this tissue.

Based on the functional enrichment in the male reproductive tissues of *A. suum* [16], our findings were concordant in terms of high phosphatase and kinase activity in the Tg and Tv tissues. The enrichment of the GO term "phosphoprotein phosphatase activity" (GO:0004721) supports the presence of high phosphatase activity, and the enrichment of three different GO terms describing kinase activity, such as "protein kinase activity" (GO:0004672), "kinase activity" (GO:0016301) and "protein serine/threonine kinase activity" (GO:0004674), supports the presence of high kinase activity. MSP was also discovered to be overexpressed in the testis of *A. suum*. Our study supported that MSP and MSP domain-containing proteins were both substantially expressed in the testes. Our study, however, also revealed that genes were more prominent in the Tg and Tv tissues.

Furthermore, our findings confirmed the presence of actin and cytoskeleton activity in the previous study, which has been reported to be crucial for nematode sperm motility and activation [46]. This was due to the enrichment of several different GO Terms that indicated actin and cytoskeleton activity in the Sv tissue, including “actin binding” (GO:0003779), “actin filament” (GO:0005884), “actin filament binding” (GO:0051015), “barbed-end actin filament capping” (GO:0051016), “cytoskeleton” (GO:0005856), “cytoskeleton organization” (GO:0007010) and “motor activity” (GO:0003774).

#### The enriched GO Terms of the female reproductive tissue samples

A total of 50, 43 and 72 GO Terms were identified for Og, Ov and Ut tissue samples, respectively, relative to the female somatic tissue. The top three GO Terms for Og tissue were binding (66 DEGs), actin binding (22 DEGs) and locomotion (22 DEGs); those for Ov tissue were binding (66 DEGs), calcium ion binding (41 DEGs) and transmembrane transport (35 DEGs); those for Ut tissue were metal ion binding (49 DEGs), structural constituent of ribosome (30 DEGs) and translation (30 DEGs) (Additional file 9: Table S6). When analyzing the GO Term based on *P*-value, the top three most significant GO Terms for Og were respiratory chain, cysteine-type peptidase activity and glutamine metabolic process; those for Ov were cysteine-type peptidase activity, cell cycle and glycolytic process; those for Ut were cytokinesis, heterotrimeric G-protein complex and serine-type endopeptidase inhibitor activity. Additional file 9: Table S6 contains the complete list of enriched GO Terms for female reproductive tissue.

**Fig. 5:**
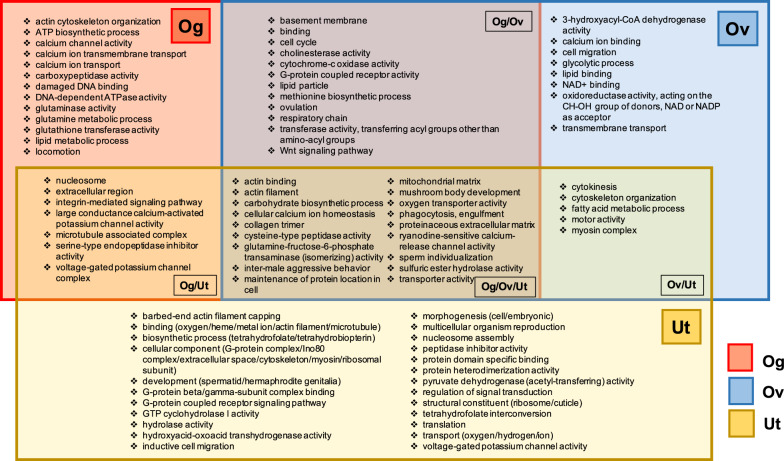
GO Term significantly enriched (*P*-value < 0.05) in the female discrete reproductive tissues. Red, blue and yellow boxes represent GO Term enriched in Og (the germinal zone of the ovary), Ov (ovary, part of oviduct) and Ut (uterus), respectively. Overlapping colored boxes represent GO Terms enriched between tissue samples. Due to the large number of GO Terms enriched within each tissue sample, groupings under general terms were used. The complete list of tissue-enriched GO Terms is shown in Additional file 9: Table S6

The high activity of cytokinesis indicative of embryo development within Ut tissue was reflected in the enriched GO Terms (Fig. 5) associated with structural components and cytoskeleton organization. Genes within these GO Terms included barbed-end actin filament capping, actin binding, microtubule binding, cytoskeleton, myosin filament and myosin complex. Furthermore, heterotrimeric G-protein complex was the second significantly enriched GO Term, indicating that genes associated with G-protein complex, including adenylate cyclase-modulating GPCR signaling pathway, G-protein beta/gamma-subunit complex binding and GPCR signaling pathway, were predominantly expressed in Ut tissue. The GO Term for GPCR activity was also found in both Og and Ov tissue samples (Fig. 5). According to the results mentioned in the previous section, the GPCR signaling pathway and small GTPase-mediated signal transduction pathway may play a pivotal role in cytoskeleton organization due to cytokinesis. Our finding highlights the roles of the GPCR signaling pathway and small GTPase-mediated signal transduction pathway in cytoskeleton organization [60, 61, 67] due to cytokinesis in oogenesis and embryogenesis of female reproductive tissue.

Rosa et al. (2014) discovered that DNA binding and replication were among the top five enriched GO terms in the ovary of *A. suum* [16]. This result was consistent with our findings, which identified several GO terms that related to these biological functions in the Og and Ov tissues, including "nucleosome" (GO:0000786), "damaged DNA binding" (GO:0003684) and "cell cycle" (GO:0007049). This result demonstrated that our approach was successful in identifying the expected function in the ovary. Moreover, our finding supported the presence of phosphatidylinositol signaling in the ovary of *A. suum* as the PIPK domain-containing protein associated with phosphatidylinositol phosphate kinase activity (GO:0016307) was identified in the Ov and Og tissues. Phosphatidylinositol signaling has been reported in the previous study to be necessary for ovulation of in *C. elegans* [68]. The previous study reported that “protein binding” (GO:0005515) and “catalytic activity” (GO:0003824) were associated with the *A. suum* uterus. Our findings, however, showed that there were other GO Terms of Ut tissue that indicated "protein binding" and "catalytic activity," such as “protein domain specific binding” (GO:0019904), “sulfuric ester hydrolase activity” (GO:0008484), “cysteine-type peptidase activity” (GO:0008234), “GTP cyclohydrolase I activity” (GO:0003934), “hydroxyacid-oxoacid transhydrogenase activity” (GO:0047988) and “glutamine-fructose-6-phosphate transaminase activity” (GO:0004360). This finding provided a comprehensive view of gene expression in *Ascaris* reproductive tissues.

### KEGG enrichment analysis

**Fig. 6:**
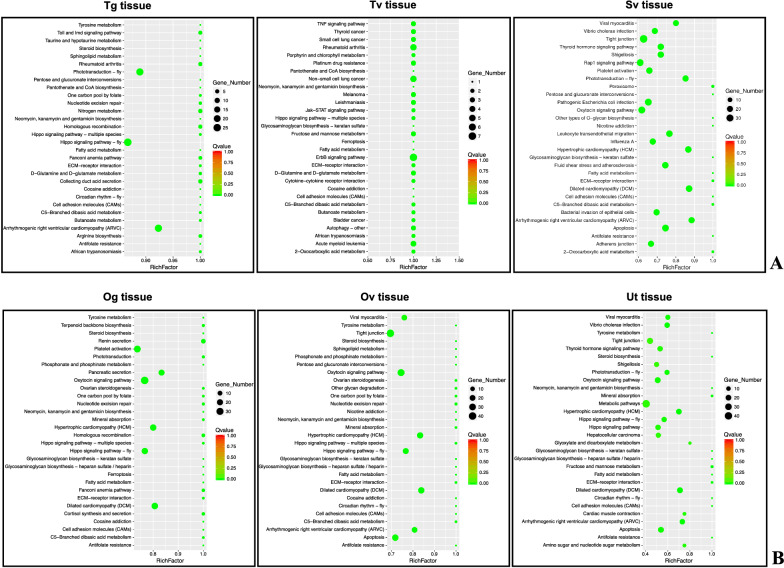
Scatter plot of differential gene KEGG enrichment of male **A** and female **B** reproductive tissues. RichFactor is on the X axis, while KEGG pathways are presented on the Y axis. The number of DEGs in the pathway is positively associated with the size of the dot. Distinct *Q*-value ranges are indicated by different color codes

On the basis of the rich factor (Fig. 6A), the top three most significant KEGG terms for Tg tissue were the Hippo signaling pathway – fly (ko04391), phototransduction – fly (ko04745) and arrhythmogenic right ventricular cardiomyopathy (ko05412). For Tv tissue, the top three most significant KEGG terms include the ErbB signaling pathway (ko04012), rheumatoid arthritis (ko05323) and non-small cell lung cancer (ko05223), whereas the top three most significant KEGG terms for Sv tissue were the rap1 signaling pathway (ko04015), oxytocin signaling pathway (ko04921) and tight junction (ko04530) (see Additional file 10: Table S7 for complete list of enriched KEGG for male reproductive tissue). In female reproductive tissues, the top three most significant KEGG terms for Og tissue were platelet activation (ko04611), the oxytocin signaling pathway and the Hippo signaling pathway-fly. Tight junction, apoptosis (ko04210) and the oxytocin signaling pathway were the top three most significant KEGG terms enriched in Ov tissue. The top three most significant KEGG terms for Ut tissue were metabolic pathways (ko01100), tight junction and shigellosis (ko05131) (Fig. 6B). The complete list of enriched KEGG for female reproductive tissue is available in Additional file 10: Table S7.

The results of the KEGG enrichment analysis indicated that the Hippo signaling pathway-fly was common to both Tg and Og tissue samples, suggesting that this pathway is predominantly specific to both male and female germinal tissue samples. Most of the genes involved in this pathway of Tg and Og tissue samples were similar, such as actin beta/gamma 1 (ACTB_G1), transcriptional enhancer factor (TEAD), 14-3-3 protein epsilon (YWHAE), serine/threonine-protein phosphatase 2A catalytic subunit (PPP2C), serine/threonine-protein phosphatase 2A regulatory subunit B (PPP2R2) and serine/threonine-protein kinase LATS1/2 (LATS1_2) (Additional file 11: Figure S4 and Additional file 12: Table S8). The Hippo signaling pathway-fly is the signaling pathway that promotes cell apoptosis and restricts organ size overgrowth, indicating that this pathway may be involved in control during the early phase of germinal cell division in oogenesis and spermatogenesis. In addition, it has been reported in *C. elegans* that the Hippo signaling pathway is essential for maintenance of apicobasal polarity in the growing intestine [69].

The oxytocin signaling pathway was indicative of both male (Sv tissue) and female reproductive tissue samples (Og and Ov tissue samples) (Additional file 11: Figure S4). In depth into the pathway, there are six genes that these tissue samples shared in common, including actin beta/gamma 1 (ACTB_G1), ryanodine receptor 2 (RYR2), Ras homolog gene family member A (RHOA), calmodulin (CALM), inositol 1,4,5-triphosphate receptor type 1 (ITPR1) and adenylate cyclase 9 (ADCY9). Although eight genes are shared by the Og and Ov tissue samples, but not the Sv tissue [atrial natriuretic peptide receptor A (ANPRA), calcium/calmodulin-dependent protein kinase I (CAMK1), guanine nucleotide-binding protein G(o) subunit alpha (GNAO), guanine nucleotide-binding protein G(s) subunit alpha (GNAS), GTPase Kras (KRAS), serine/threonine-protein phosphatase PP1 catalytic subunit (PPP1C), serine/threonine-protein phosphatase 2B catalytic subunit (PPP3C) and 5′-AMP-activated protein kinase, catalytic alpha subunit (PRKAA)], this indicates that certain genes play a role in Og and Ov tissue.

Furthermore, elongation factor 2 (EEF2), guanine nucleotide-binding protein G(q) subunit alpha (GNAQ) and classical protein kinase C alpha type (PRKCA) were shown to be specific for Og, Ov and Sv, respectively. Additional file 12: Table S8 contains the complete list of significant DEGs annotated in KEGG enrichment.

Although oxytocin neuropeptides are strongly implicated in mammalian reproductive and social behaviors, an oxytocin/vasopressin-like signaling pathway was identified in sexually dimorphic patterns of *C. elegans*. This pathway involves a peptide, nematocin (encoded by *ntc*-1) and two receptors (encoded by *ntr*-1 and *ntr*-2), which are associated with the GPCR superfamily [70]. Mutations in the *ntc*-1 gene or its receptors cause defects in reproductive behavior, such as mate searching, mate recognition and mating [71]. As a result, the properties and functions of the signaling molecules involved in the oxytocin signaling pathway in *Ascaris* should be further investigated to identify prospective therapeutic targets.

Tight junction was another crucial pathway discovered in both male and female reproductive tissue samples (Additional file 11: Figure S4). Several genes could be commonly identified in Ov, Ut and Sv tissue samples, including actin beta/gamma 1 (ACTB_G1), integrin beta 1 (ITGB1, CD29), myosin heavy chain (MYH), E3 ubiquitin-protein ligase NEDD4-like (NEDD4L), Ras-related protein Rab-8A (RAB8A, MEL) and tubulin alpha. Additionally, myosin regulatory light chain 2 (MYL2), Ras-related C3 botulinum toxin substrate 1 (RAC1) and Ras homolog gene family, member A (RHOA) were discovered in Ov and Sv tissue samples but not in Ut tissue. Although serine/threonine-protein phosphatase 2A catalytic subunit (PPP2C), 5′-AMP-activated protein kinase, catalytic alpha subunit (PRKAA, AMPK), atypical protein kinase C iota type (PRKCI) and Ras-related protein Rap-1A (RAP1A) were specifically discovered in Ov tissue, this suggests that those genes play a unique role in Ov tissue (Additional file 12: Table S8). According to pathway mapping, genes involved in this pathway are associated with actin assembly, adherens junction assembly, tight assembly, cell polarity and cell migration, implying that chromosome organization is active because of cell division in reproductive tissue.

### Validation of gene expression by RT-qPCR

Seven tissue-specific genes were selected as a representative of each cluster. All selected tissue-specific genes demonstrated consistent expression, as revealed in the hierarchical clustering analysis (Additional file 13: Figure S5). ALUE_0000279901, ALUE_0002034701, ALUE_0001490701, ALUE_0000300701, ALUE_0000531301, ALUE_0000064301 and ALUE_0001851601 genes were expressed specifically to Tg, Sv, Ut, Stf/Stm, Tv/Tg, Og/Ov and Ov/Og tissue samples, respectively. On the basis of our transcriptome data, RT-qPCR was performed in this study using two putative housekeeping genes, namely, eIF6 and NADH cytochrome b5 reductase genes. However, only the eIF6 gene was discovered to display stable gene expression in all reproductive tissue samples and could be used to normalize target gene expression.

## Conclusion

This study generated transcriptome profiles of discrete reproductive and somatic tissue samples from male and female *A. lumbricoides*. On the basis of the FPKM profiles, gene expression analysis was used to identify DEGs from each distinct tissue type examined. Hierarchical clustering analysis identified seven clusters associated with specific tissues and groups of tissues. The findings revealed that DEGs involved in protein phosphorylation were differentially expressed particularly in Tv and Tg tissue samples and played a crucial role in spermatogenesis. Although DEGs were shared between Tg and Tv tissue samples, adhesion molecules were specifically identified in Sv tissue and played a critical role in the fertilization process. Several DEGs associated with the cell division cycle and transcription process were identified in Og and Ov tissue samples. DEGs involved in GPCR signaling pathway and small GTPase-mediated signal transduction pathway were discovered to play a crucial role in cytoskeleton organization due to oogenesis in Ov and Og tissue samples. DEGs associated with the SMA genes and TGF-β signaling pathway were discovered to be crucial in the embryogenesis of Ut tissue. Additionally, Hippo signaling, oxytocin signaling and tight junction pathways were identified to play a role in *Ascaris* male and female reproductive systems. Some of the genes discovered in this study that are related to protein phosphorylation, the TGF-signaling system and the oxytocin signaling pathway have been linked to defects in germline development, embryogenesis and reproductive behavior. In addition to providing the transcriptome profiles of discrete reproductive tissue samples of *A. lumbricoides*, the results of this study highlight genes that are likely to play significant roles in the functions of these important tissues. Data generated here will inform appropriate selection of gene targets for therapeutic exploitation to aid future sustained control of ascariasis.

## Supplementary Information

**Additional file 1: Figure S1.** The dissection of somatic and reproductive tissues.

**Additional file 2: Table S1.** The primer list for constructing library and validating gene expression.

**Additional file 3: Table S2**. Sequencing data and quality assessment of *A. lumbricoides* discrete tissue samples.

**Additional file 4: Table S3**. Raw data, filtered data, and data alignment statistics.

**Additional file 5: Figure S2.** The distribution of reads in different genomic regions of somatic and reproductive tissue samples.

**Additional file 6: Figure S3.** Overall quality assessment of RNA sequencing.

**Additional file 7: Table S4.** The complete list of significant DEGs comparing between the tissue samples.

**Additional file 8: Table S5.** List of all genes for hierarchical clustering of DEGs.

**Additional file 9: Table S6.** The complete list of GO Terms significantly enriched in *A. lumbricoides* discrete reproductive tissues.

**Additional file 10: Table S7.** The complete list of KEGG pathway significantly enriched in *A. lumbricoides* discrete reproductive tissues.

**Additional file 11: Figure S4.** Demonstration of important signaling pathways, including the Hippo signaling pathway-fly, Oxytocin signaling pathway and tight junction pathway

**Additional file 12: Table S8.** The complete list of significant DEGs annotated in KEGG enrichment.

**Additional file 13: Figure S5.** Gene expression level of tissue-specific genes in *A. lumbricoides* discrete tissues

## Data Availability

RNA-Seq reads have been deposited in the NCBI-Sequence Read Archive (SRA) database under BioProject: PRJNA775847. (https://dataview.ncbi.nlm.nih.gov/object/PRJNA775847?reviewer=haofctjm3ludqsdjh9iik3fv5r).
