# Supplementary material for: Transcriptome profiling of male and female Ascaris lumbricoides reproductive tissues
Source: Parasit Vectors. 2022 Dec 20;15:477. doi: 10.1186/s13071-022-05602-2 (PMC9768952; doi:10.1186/s13071-022-05602-2)

## Slide 1
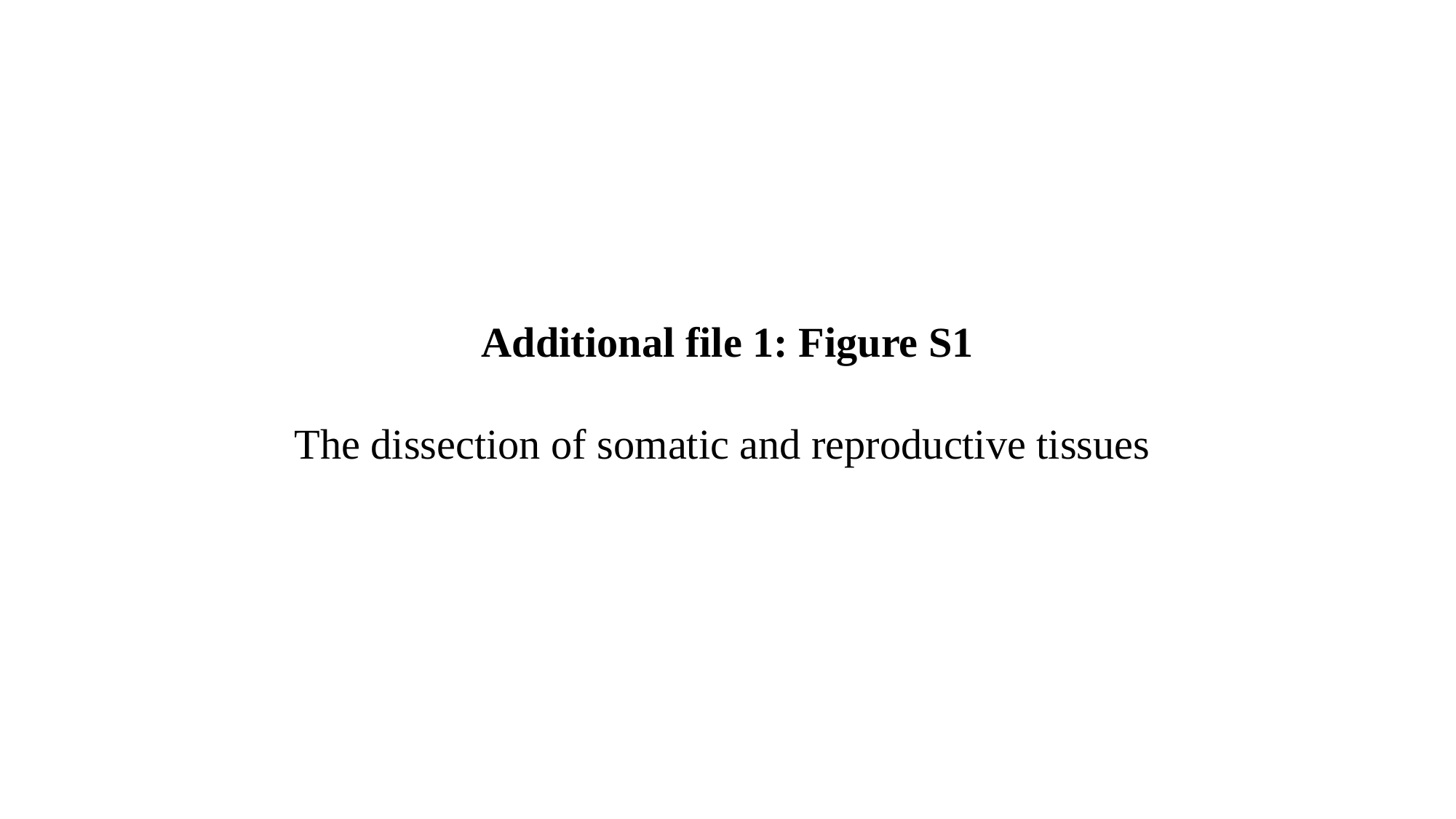

Additional file 1: Figure S1
The dissection of somatic and reproductive tissues

## Slide 2
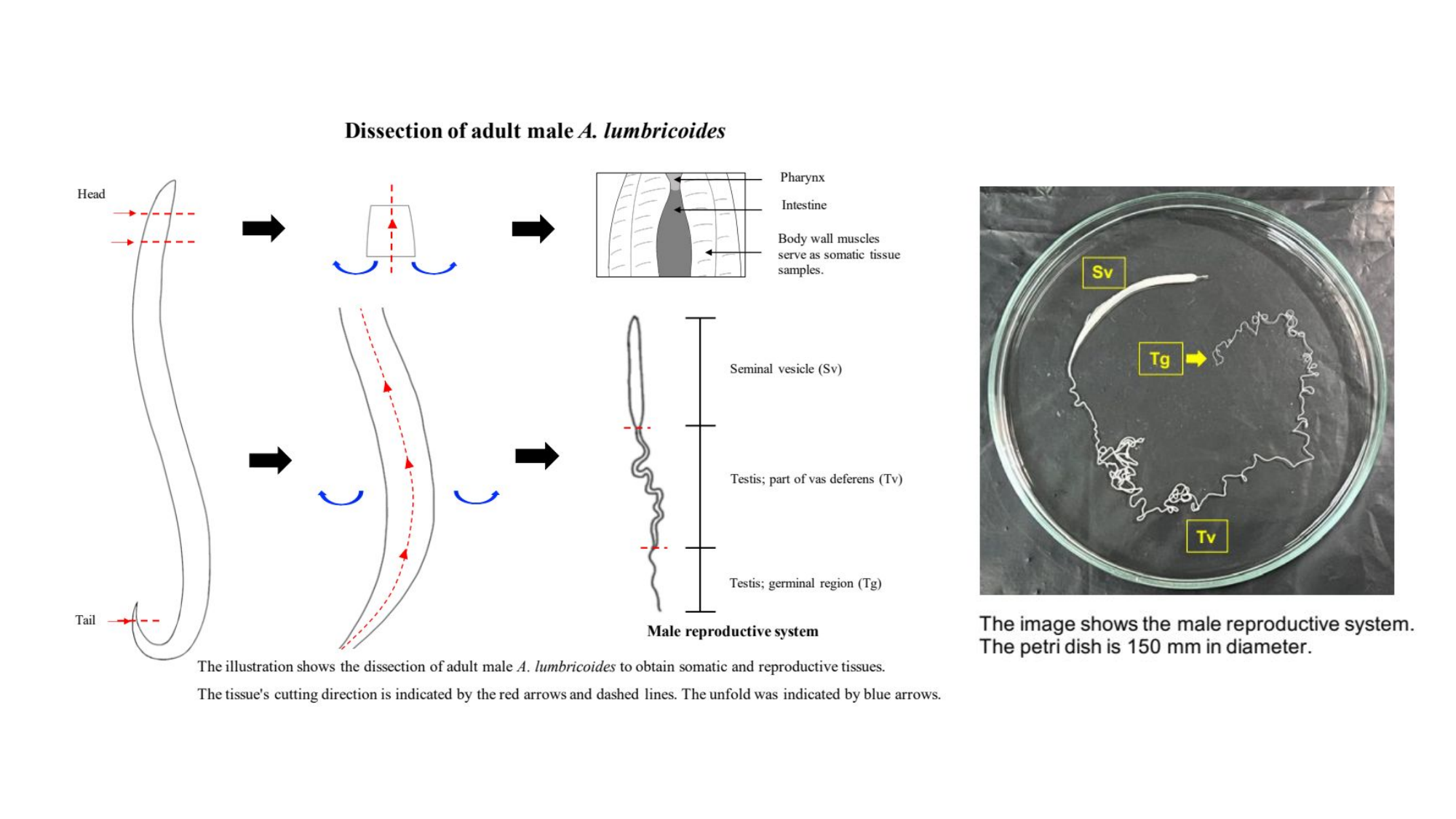

## Slide 3
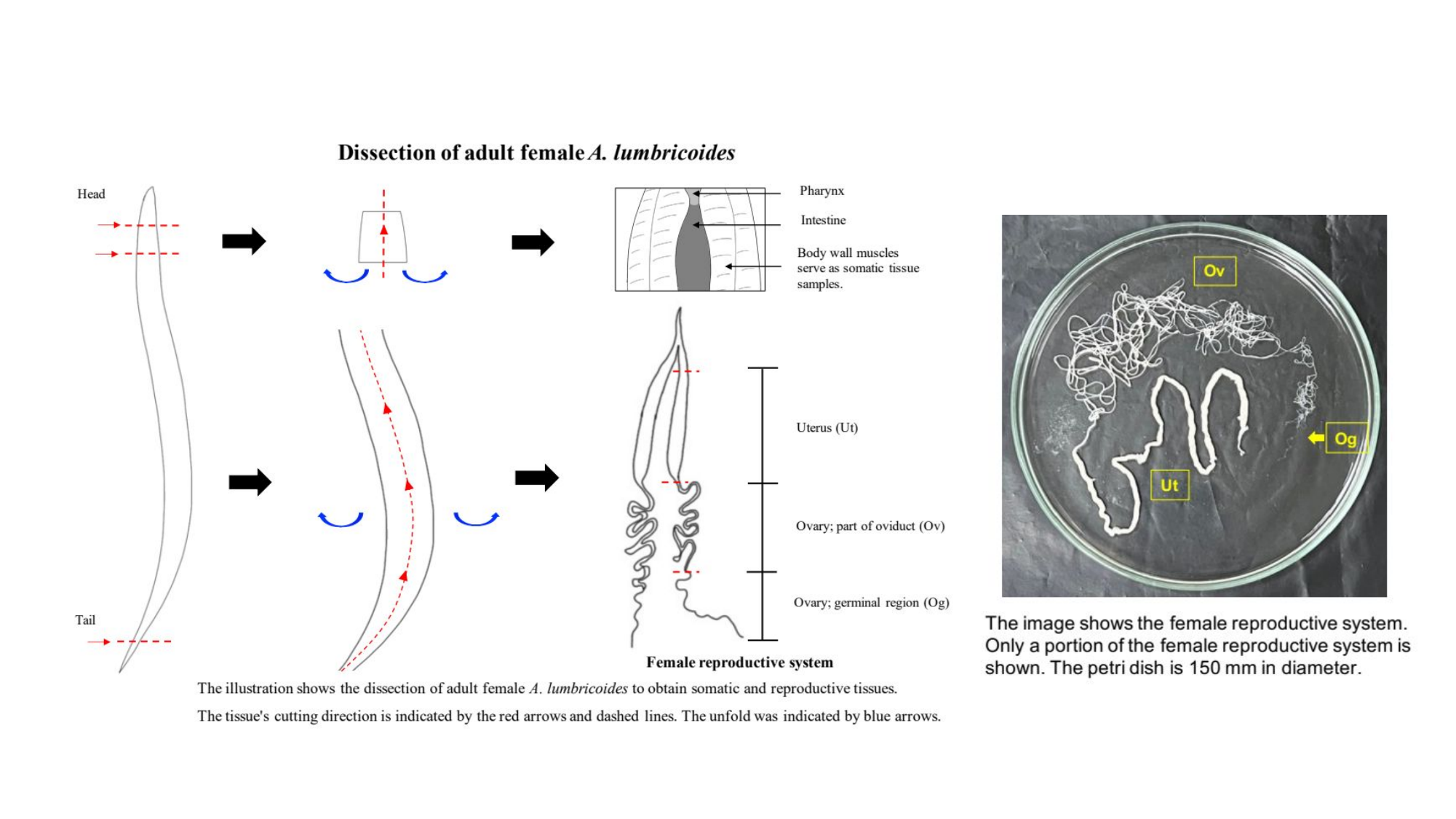

Supplement: Supplementary file 1 — Additional file 1: Figure S1. The dissection of somatic and reproductive tissues. [file 13071_2022_5602_MOESM1_ESM.pptx]
