# Supplementary material for: Transcriptome profiling of male and female Ascaris lumbricoides reproductive tissues
Source: Parasit Vectors. 2022 Dec 20;15:477. doi: 10.1186/s13071-022-05602-2 (PMC9768952; doi:10.1186/s13071-022-05602-2)
Supplement: Supplementary file 2 — Additional file 2: Table S1. The primer list for constructing library and validating gene expression. [file 13071_2022_5602_MOESM2_ESM.docx]

**Additional file 2: Table S1**. The primer sequences using in the library construction are shown below.

| **Primer** | **Sequences (5′ - 3′)** |
| --- | --- |
| P7 primer | CAAGCAGAAGACGGCATACGAGAT |
| P5 primer | AATGATACGGCGACCACCGAGATC |

**Additional file 2: Table S1**. The oligonucleotide primers used to validate gene expression in this study.

| **No** | **Primer name** |  | **Sequences (5′ - 3′)** | **bp** | **Length** | **Description (BlastX)** |
| --- | --- | --- | --- | --- | --- | --- |
| 1 | ALUE_0000324401-mRNA-1 | F | TGCAGCACATAAGGAACAGC | 20 | 112 bp | Eukaryotic translation initiation factor 6 (EIF6) |
|  |  | R | TGTACAATCGCCACATGGTC | 20 |  |  |
| 2 | ALUE_0001090701-mRNA-1 | F | CAAGTTGCCCTCAGAAAAGC | 20 | 104 bp | NADH cytochrome b5 reductase |
|  |  | R | TTGGCGTATATGGACGAACC | 20 |  |  |
| 3 | ALUE_0000300701-mRNA-1 | F | TGGCCAGGATAAGAATACGC | 20 | 138 bp | Chymotrypsin/elastase isoinhibitor 1 [Toxocara canis] |
|  |  | R | GTTCCGGGGAAGTTTATTGG | 20 |  |  |
| 4 | ALUE_0000531301-mRNA-1 | F | AAGAGCAATCTCGGCTGTTG | 20 | 71 bp | unnamed protein product [Toxocara canis] |
|  |  | R | GCTGCGATGGTATTGTTGTC | 20 |  |  |
| 5 | ALUE_0000279901-mRNA-1 | F | ACTGCGTCACAAGATGAACG | 20 | 74 bp | Uncharacterized protein Tcan_18476 [Toxocara canis] |
|  |  | R | GACAAAAGAGACGCCAAACC | 20 |  |  |
| 6 | ALUE_0002034701-mRNA-1 | F | TAACAGATTTGGGGCGACAG | 20 | 131 bp | hypothetical protein Tcan_12600 [Toxocara canis] |
|  |  | R | GATCACCATCGGTTTGTTGG | 20 |  |  |
| 7 | ALUE_0001490701-mRNA-1 | F | TTCTCGTGGTGCTTCAAGTG | 20 | 100 bp | hypothetical protein Tcan_13872 [Toxocara canis] |
|  |  | R | GGATTAGGCTTCACGCAATG | 20 |  |  |
| 8 | ALUE_0000064301-mRNA-1 | F | TCGGGTACGGTCATATTTGC | 20 | 73 bp | TWiK family of potassium channels protein 7 [Toxocara canis] |
|  |  | R | ATTCCAACCGTTGCGTAGAG | 20 |  |  |
| 9 | ALUE_0001851601-mRNA-1 | F | ATCTGCACTTGCTTGCACAC | 20 | 149 bp | neuropeptide receptor 2, partial [Ascaris suum] |
|  |  | R | CAAACATCACCACCATGCTC | 20 |  |  |

F: Forward primer, R: Reverse primer
