# Supplementary material for: Transcriptome profiling of male and female Ascaris lumbricoides reproductive tissues
Source: Parasit Vectors. 2022 Dec 20;15:477. doi: 10.1186/s13071-022-05602-2 (PMC9768952; doi:10.1186/s13071-022-05602-2)
Supplement: Supplementary file 3 — Additional file 3: Table S2. Sequencing data and quality assessment of A. lumbricoides discrete tissue samples. [file 13071_2022_5602_MOESM3_ESM.docx]

**Additional file 3: Table S2** Sequencing data and quality assessment of *A. lumbricoides* discrete tissue samples.

| **Sample** | **Replicate** | **Number**  **of raw reads**  **(Millions)** | **Average read length** | **Number**  **of clean reads**  **(Millions)** | **Number of**  **bases** | **Q20^a^**  **(%)** | **Q30^a^**  **(%)** | **GC**  **(%)** | **N^b^**  **(ppm)** | **Total reads**  **(Millions)** | **Total mapped reads^c^**  **(%)** | **Uniquely mapped reads^c^**  **(%)** | **Multiple mapped reads^c^**  **(%)** |
| --- | --- | --- | --- | --- | --- | --- | --- | --- | --- | --- | --- | --- | --- |
| Tg | Tg-1 | 41.8 | 148.5 | 41.7 | 6.2E-09 | 98.3 | 94.7 | 46.9 | 11.6 | 41.7 | 32.5 (78.1%) | 28.0 (67.1%) | 4.6  (11.0%) |
|  | Tg-2 | 43.0 | 148.8 | 42.9 | 6.4E-09 | 98.2 | 94.5 | 46.6 | 1.9 | 42.9 | 37.2 (86.6%) | 32.2 (75.1%) | 4.9  (11.5%) |
|  | Tg-3 | 43.8 | 148.3 | 43.6 | 6.5E-09 | 98.2 | 94.5 | 47.2 | 15.4 | 43.6 | 37.9 (87.0%) | 32.5 (74.5%) | 5.5  (12.5%) |
| Tv | Tv-1 | 41.8 | 148.5 | 41.7 | 6.1871E-09 | 98.2 | 94.5 | 46.9 | 11.6 | 41.7 | 32.1 (77.0%) | 25.7 (61.7%) | 6.4  (15.3%) |
|  | Tv-2 | 43.1 | 148.8 | 43.0 | 6.4E-09 | 97.9 | 93.9 | 46.7 | 15.3 | 43.0 | 36.3 (84.6%) | 29.3 (68.3%) | 7.0  (16.3%) |
|  | Tv-3 | 48.2 | 148.3 | 48.0 | 7.1E-09 | 97.9 | 93.8 | 46.8 | 15.0 | 48.0 | 39.4 (82.1%) | 30.3 (63.1%) | 9.1  (19.0%) |
| Sv | Sv-1 | 45.2 | 148.7 | 45.1 | 6.7E-09 | 98.5 | 95.2 | 46.3 | 11.6 | 45.1 | 38.9 (86.2%) | 33.2 (73.6%) | 5.7  (12.6%) |
|  | Sv-2 | 50.0 | 148.6 | 49.9 | 7.4E-09 | 98.3 | 94.9 | 46.9 | 15.2 | 49.9 | 44.5 (89.3%) | 38.6 (77.5%) | 5.9  (11.8%) |
|  | Sv-3 | 48.8 | 148.7 | 48.7 | 7.2E-09 | 98.3 | 94.8 | 46.9 | 15.4 | 48.7 | 43.7 (89.7%) | 37.9 (77.8%) | 5.8  (11.9%) |
| Stm | Stm-1 | 42.5 | 148.6 | 42.4 | 6.3E-09 | 98.25 | 94.75 | 47.96 | 15.63 | 42.4 | 37.8 (89.3%) | 33.9 (80.1%) | 3.9  (9.2%) |
|  | Stm-2 | 42.4 | 148.5 | 42.2 | 6.3E-09 | 98.24 | 94.8 | 47.55 | 15.62 | 42.2 | 37.0 (87.6%) | 33.1 (78.4%) | 3.9  (9.2%) |
|  | Stm-3 | 43.1 | 148.7 | 43.0 | 6.4E-09 | 98.34 | 94.97 | 47.97 | 15.11 | 43.0 | 37.4 (87.1%) | 33.3 (77.4%) | 4.1  (9.6%) |
| Og | Og-1 | 49.9 | 148.0 | 49.7 | 7.3E-09 | 98.2 | 94.9 | 49.2 | 15.4 | 49.7 | 44.6 (89.9%) | 40.5 (81.5%) | 4.2  (8.4%) |
|  | Og-2 | 43.7 | 148.4 | 43.4 | 6.4E-09 | 98.2 | 94.6 | 48.9 | 15.3 | 43.4 | 39.1 (90.0%) | 35.5 (81.8%) | 3.6  (8.3%) |
|  | Og-3 | 43.5 | 148.7 | 43.3 | 6.4E-09 | 98.2 | 94.8 | 48.5 | 15.3 | 43.3 | 38.8 (89.7%) | 35.3 (81.5%) | 3.5  (8.1%) |
| Ov | Ov-1 | 42.6 | 148.8 | 42.5 | 6.3E-09 | 98.2 | 94.6 | 48.1 | 15.1 | 42.5 | 38.2 (90.0%) | 34.7 (81.7%) | 3.5  (8.3%) |
|  | Ov-2 | 49.9 | 148.7 | 49.7 | 7.4E-09 | 98.2 | 94.8 | 48.1 | 15.1 | 49.7 | 44.5 (89.5%) | 40.3 (81.0%) | 4.2  (8.5%) |
|  | Ov-3 | 43.3 | 148.9 | 43.2 | 6.4E-09 | 98.2 | 94.8 | 48.6 | 15.2 | 43.2 | 38.8 (89.9%) | 35.3 (81.8%) | 3.5  (8.2%) |
| Ut | Ut-1 | 49.3 | 148.1 | 48.9 | 7.2E-09 | 97.4 | 93.3 | 48.8 | 15.3 | 48.9 | 33.0 (67.6%) | 30.0 (61.3%) | 3.0  (6.2%) |
|  | Ut-2 | 42.1 | 148.3 | 41.8 | 6.2E-09 | 97.2 | 92.9 | 48.7 | 15.2 | 41.8 | 27.1 (64.9%) | 24.2 (57.9%) | 2.9  (6.9%) |
|  | Ut-3 | 45.1 | 148.0 | 44.7 | 6.6E-09 | 96.6 | 92.0 | 48.6 | 15.1 | 44.7 | 30.0 (67.1%) | 27.3 (61.1%) | 2.7  (6.1%) |
| Stf | Stf-1 | 44.0 | 148.7 | 43.8 | 6.5E-09 | 98.4 | 95.0 | 47.3 | 15.4 | 43.8 | 38.8 (88.5%) | 34.9 (79.5%) | 3.9  (8.9%) |
|  | Stf-2 | 43.7 | 148.7 | 43.6 | 6.5E-09 | 98.3 | 95.0 | 48.0 | 15.4 | 43.6 | 38.5 (88.2%) | 34.6 (79.3%) | 3.9  (8.9%) |
|  | Stf-3 | 40.7 | 144.9 | 40.1 | 5.8E-09 | 98.6 | 95.5 | 49.3 | 15.3 | 40.1 | 36.1 (89.9%) | 32.7 (81.4%) | 3.4  (8.5%) |

^a^ Q20 or Q30 = The percentage of bases with quality scores (Phred quality score) higher than 20 or 30. ^b^ N(ppm) = The number of base 'N' per million bases. ^c^ Million reads. Tg: the germinal zone of the testis, Tv: testis, part of vas deferens, Sv: seminal vesicle, Stm: male somatic tissue, Og: the germinal zone of the ovary, Ov: ovary, part of the oviduct, Ut: uterus, Stf: female somatic tissue.
