# Supplementary material for: Transcriptome profiling of male and female Ascaris lumbricoides reproductive tissues
Source: Parasit Vectors. 2022 Dec 20;15:477. doi: 10.1186/s13071-022-05602-2 (PMC9768952; doi:10.1186/s13071-022-05602-2)
Supplement: Supplementary file 5 — Additional file 5: Figure S2. The distribution of reads in different genomic regions of somatic and reproductive tissue samples. [file 13071_2022_5602_MOESM5_ESM.pptx]

## Slide 1
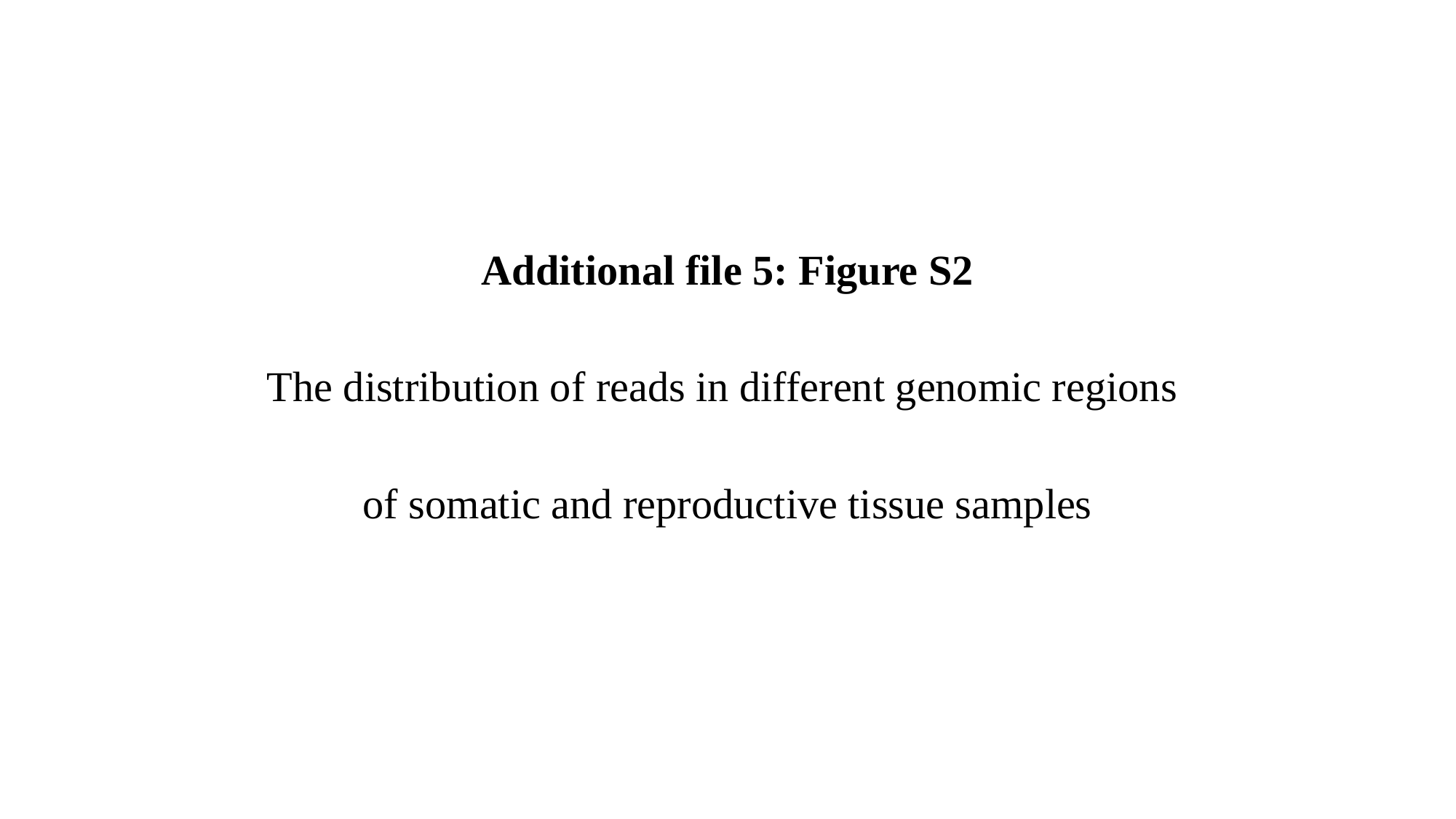

Additional file 5: Figure S2
The distribution of reads in different genomic regions
of somatic and reproductive tissue samples

## Slide 2
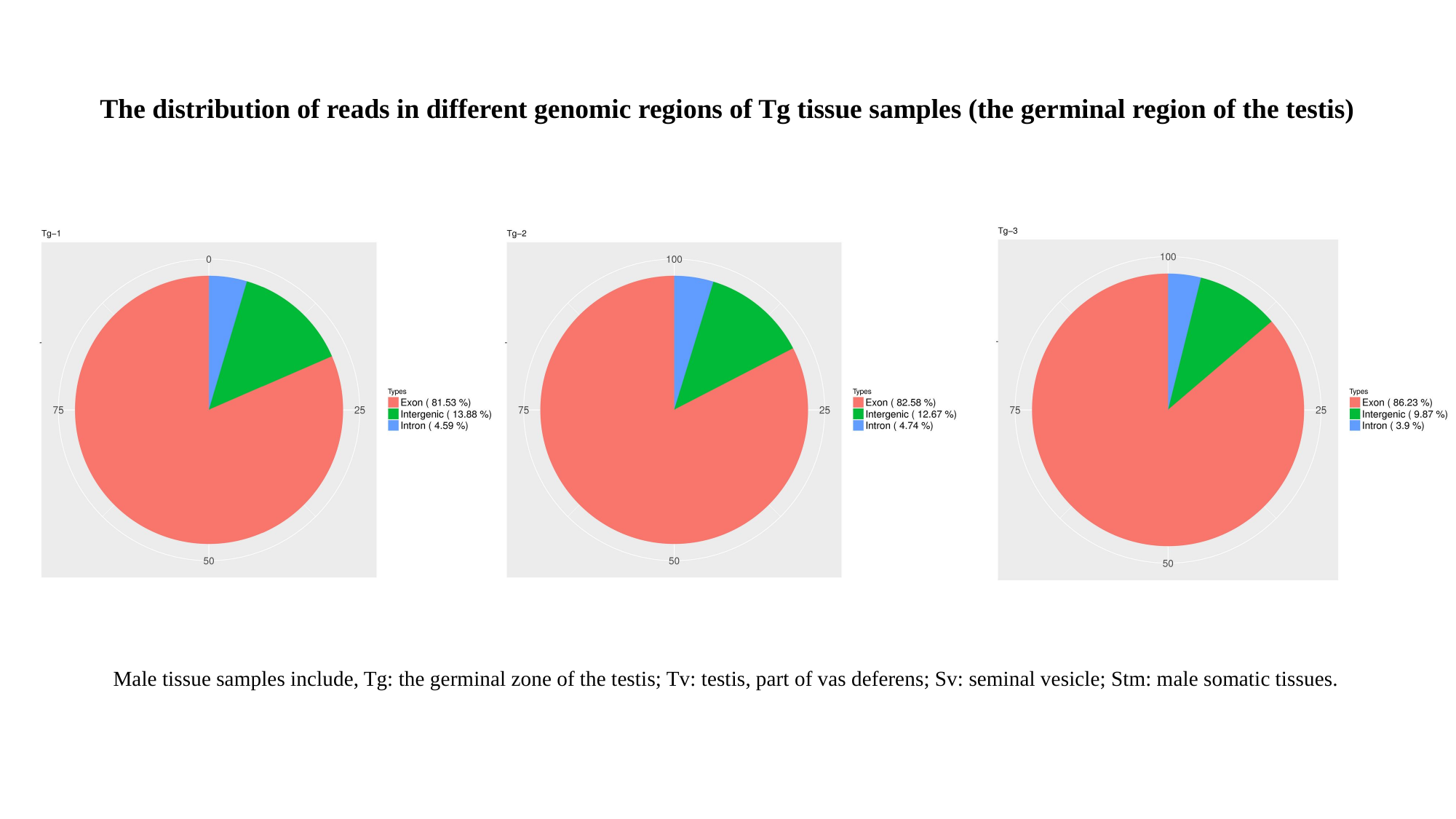

The distribution of reads in different genomic regions of Tg tissue samples (the germinal region of the testis)
Male tissue samples include, Tg: the germinal zone of the testis; Tv: testis, part of vas deferens; Sv: seminal vesicle; Stm: male somatic tissues.

## Slide 3
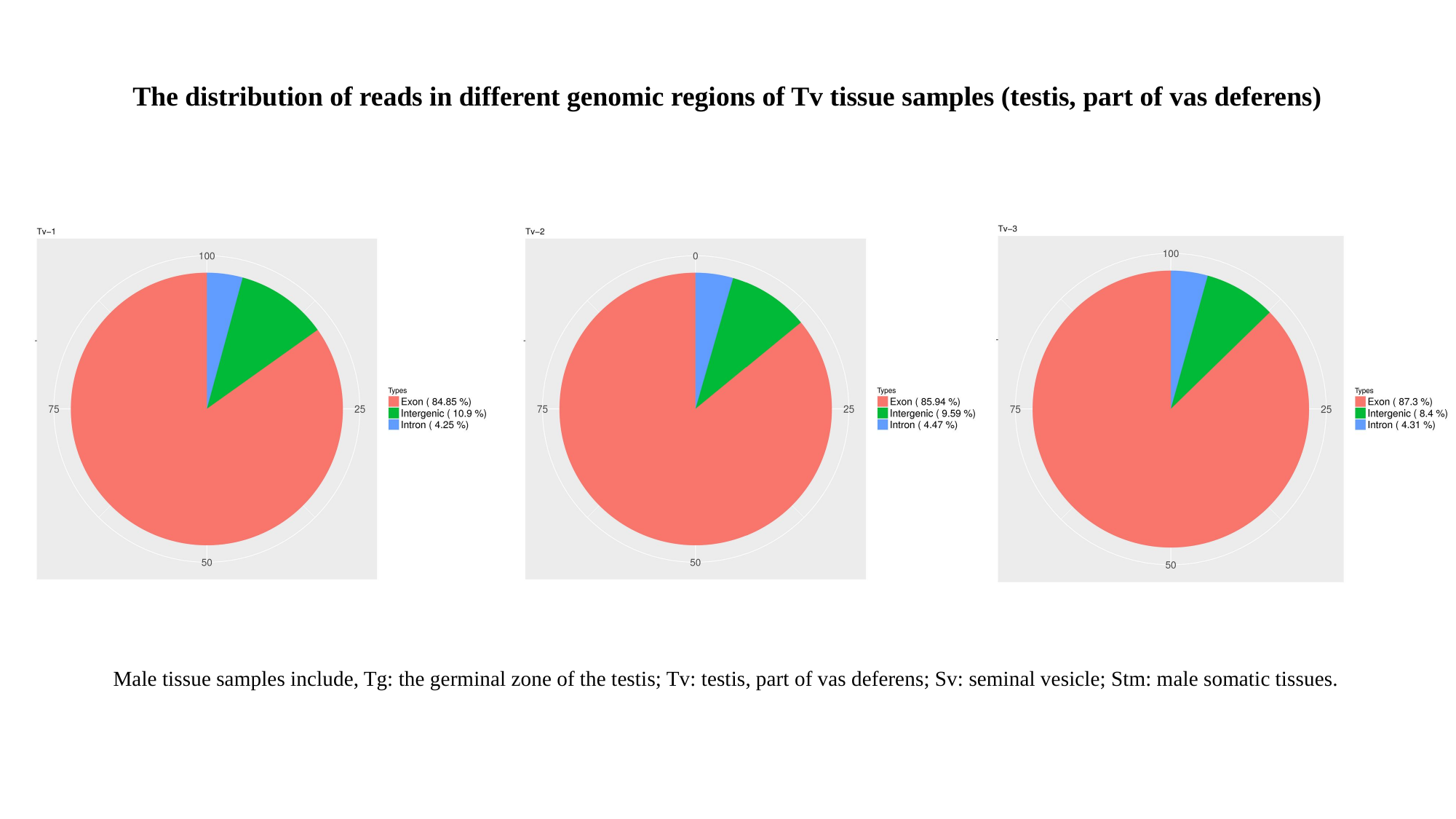

The distribution of reads in different genomic regions of Tv tissue samples (testis, part of vas deferens)
Male tissue samples include, Tg: the germinal zone of the testis; Tv: testis, part of vas deferens; Sv: seminal vesicle; Stm: male somatic tissues.

## Slide 4
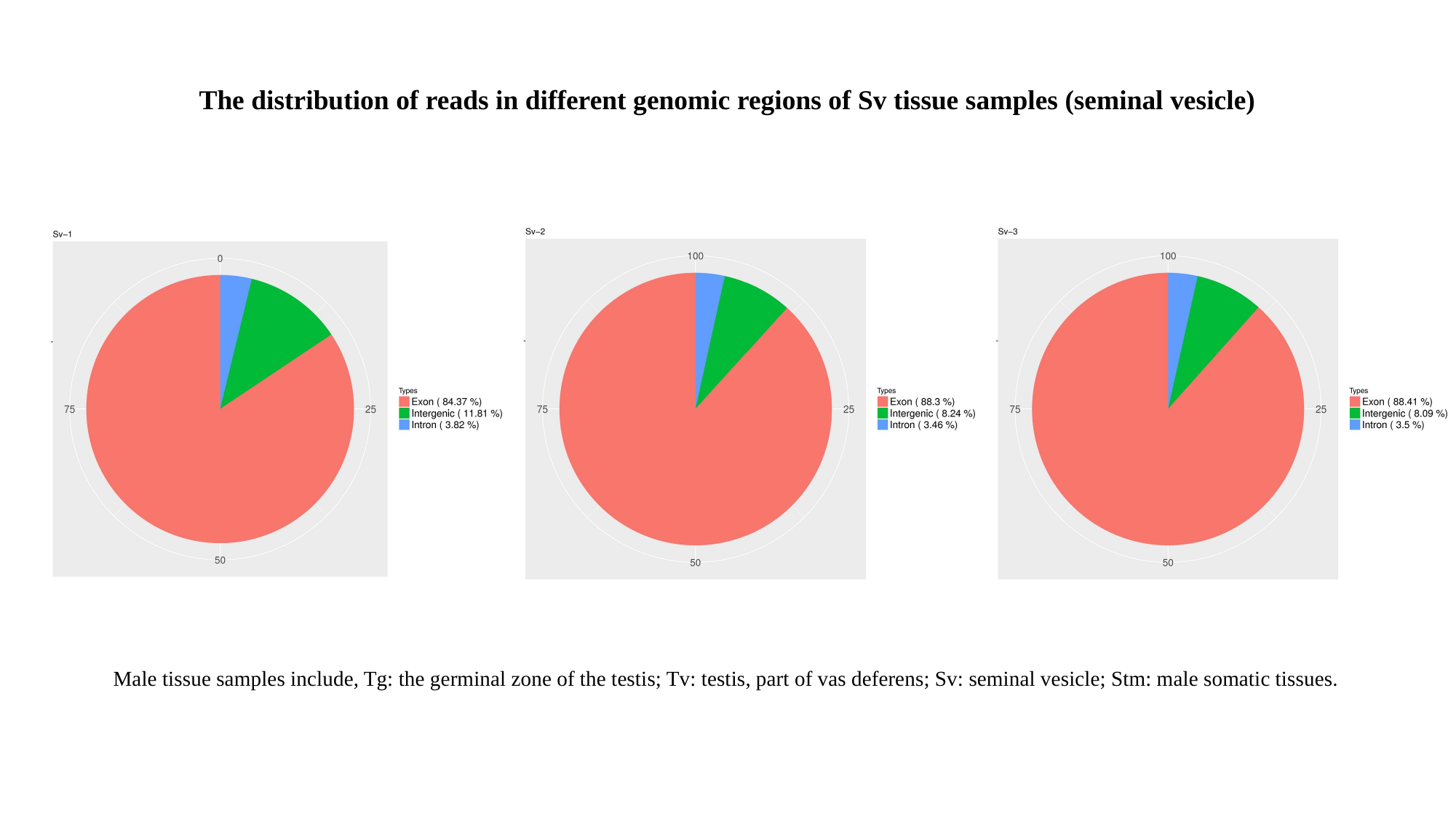

The distribution of reads in different genomic regions of Sv tissue samples (seminal vesicle)
Male tissue samples include, Tg: the germinal zone of the testis; Tv: testis, part of vas deferens; Sv: seminal vesicle; Stm: male somatic tissues.

## Slide 5
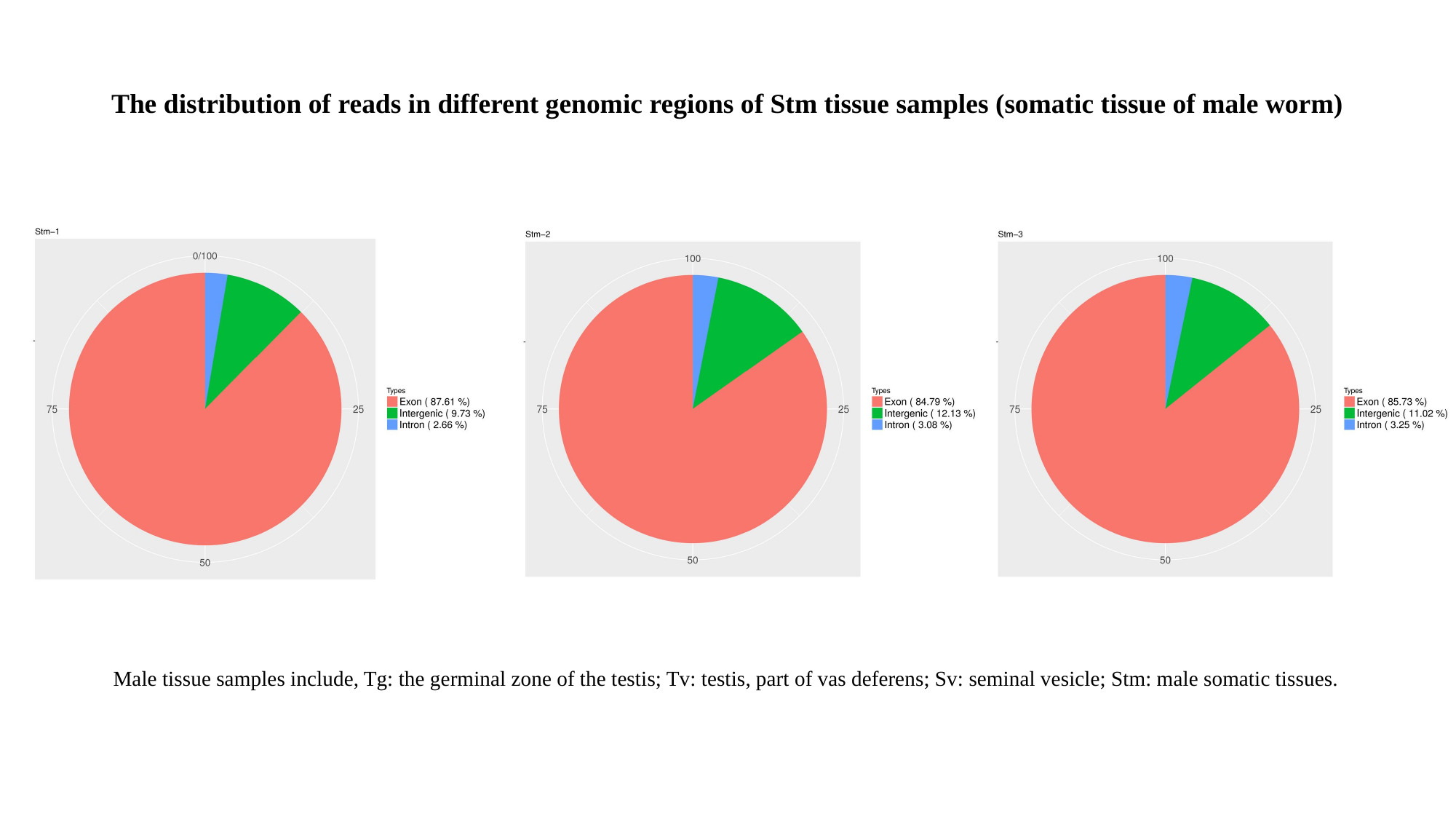

The distribution of reads in different genomic regions of Stm tissue samples (somatic tissue of male worm)
Male tissue samples include, Tg: the germinal zone of the testis; Tv: testis, part of vas deferens; Sv: seminal vesicle; Stm: male somatic tissues.

## Slide 6
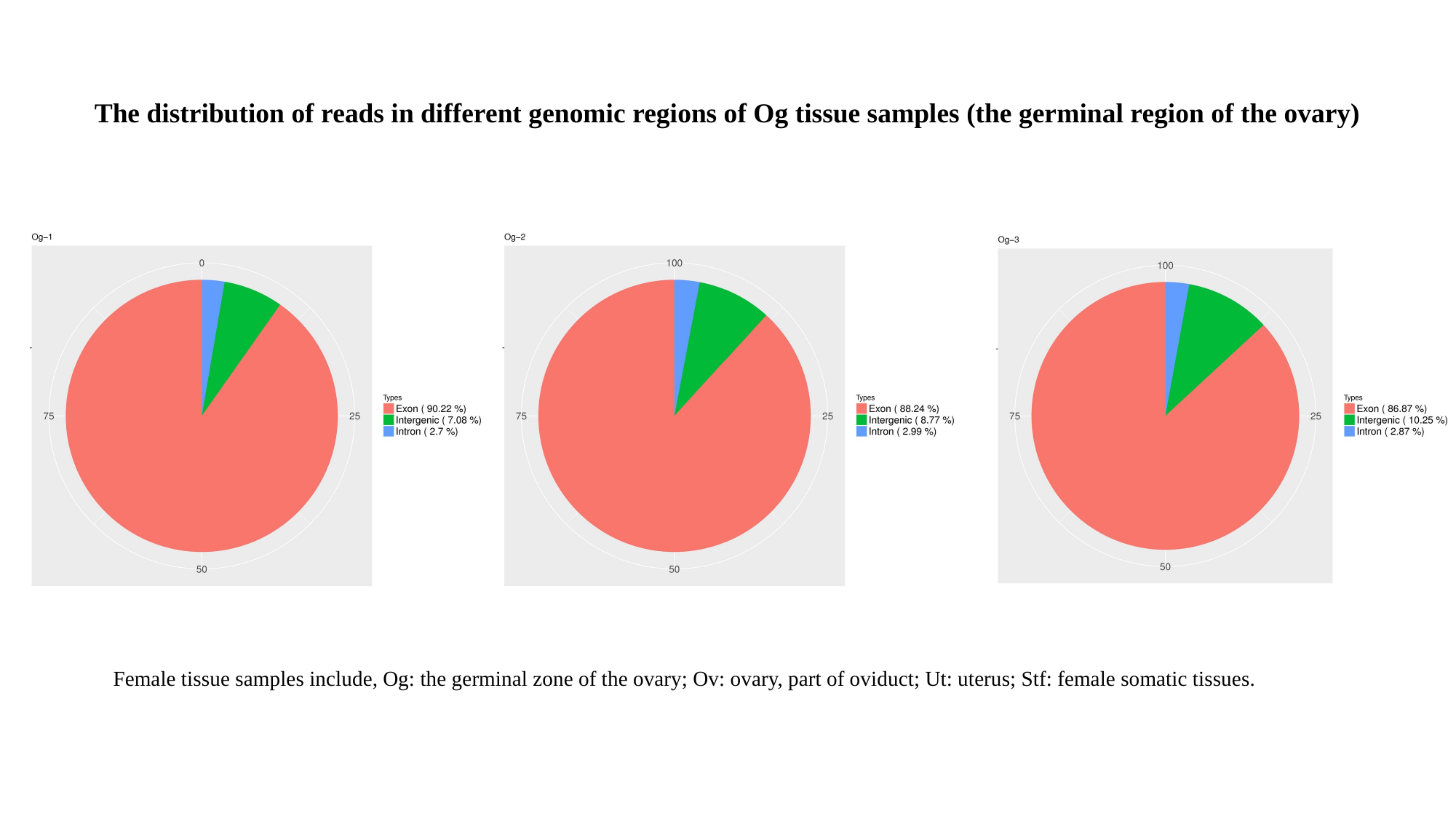

The distribution of reads in different genomic regions of Og tissue samples (the germinal region of the ovary)
Female tissue samples include, Og: the germinal zone of the ovary; Ov: ovary, part of oviduct; Ut: uterus; Stf: female somatic tissues.

## Slide 7
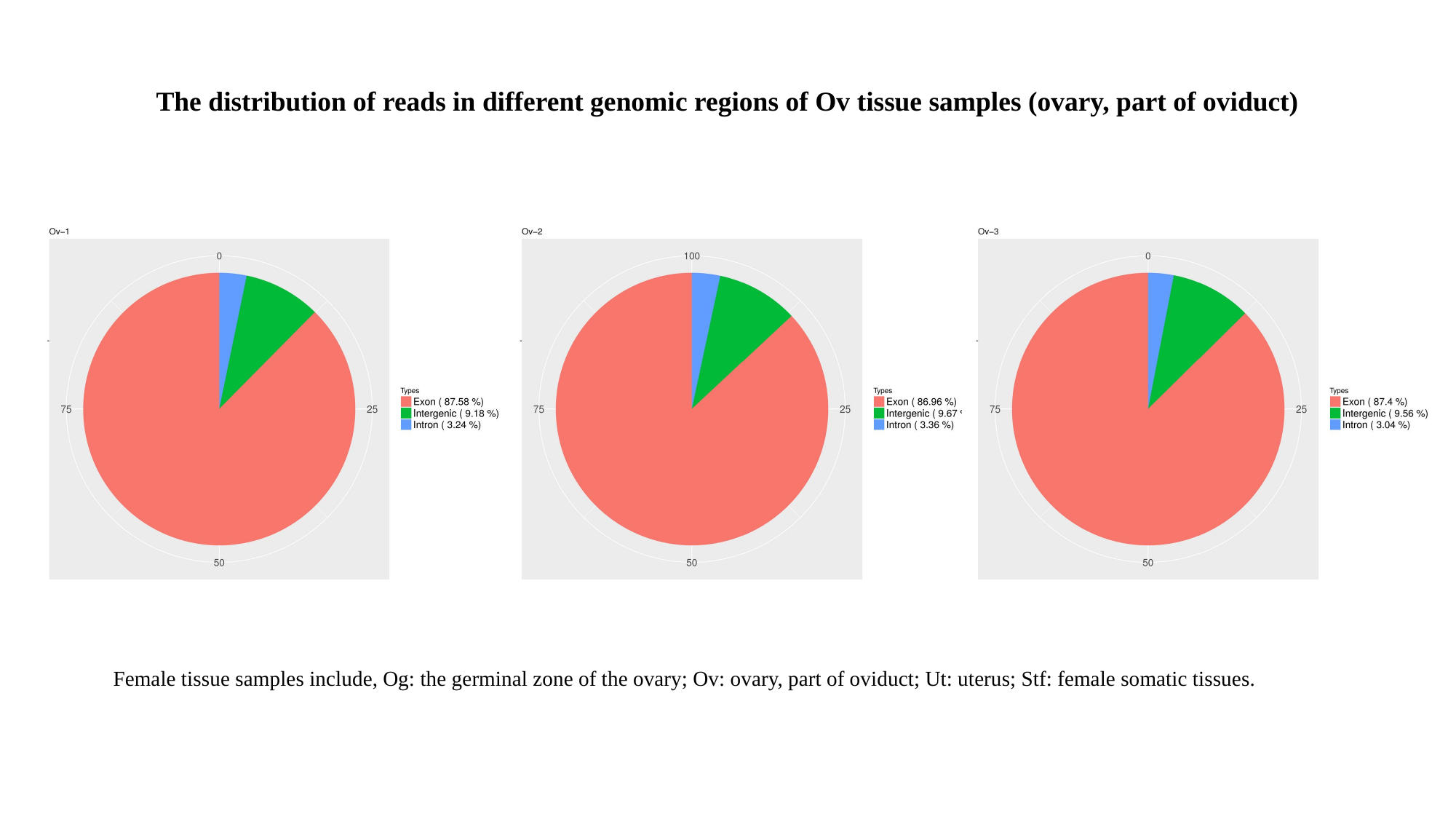

The distribution of reads in different genomic regions of Ov tissue samples (ovary, part of oviduct)
Female tissue samples include, Og: the germinal zone of the ovary; Ov: ovary, part of oviduct; Ut: uterus; Stf: female somatic tissues.

## Slide 8
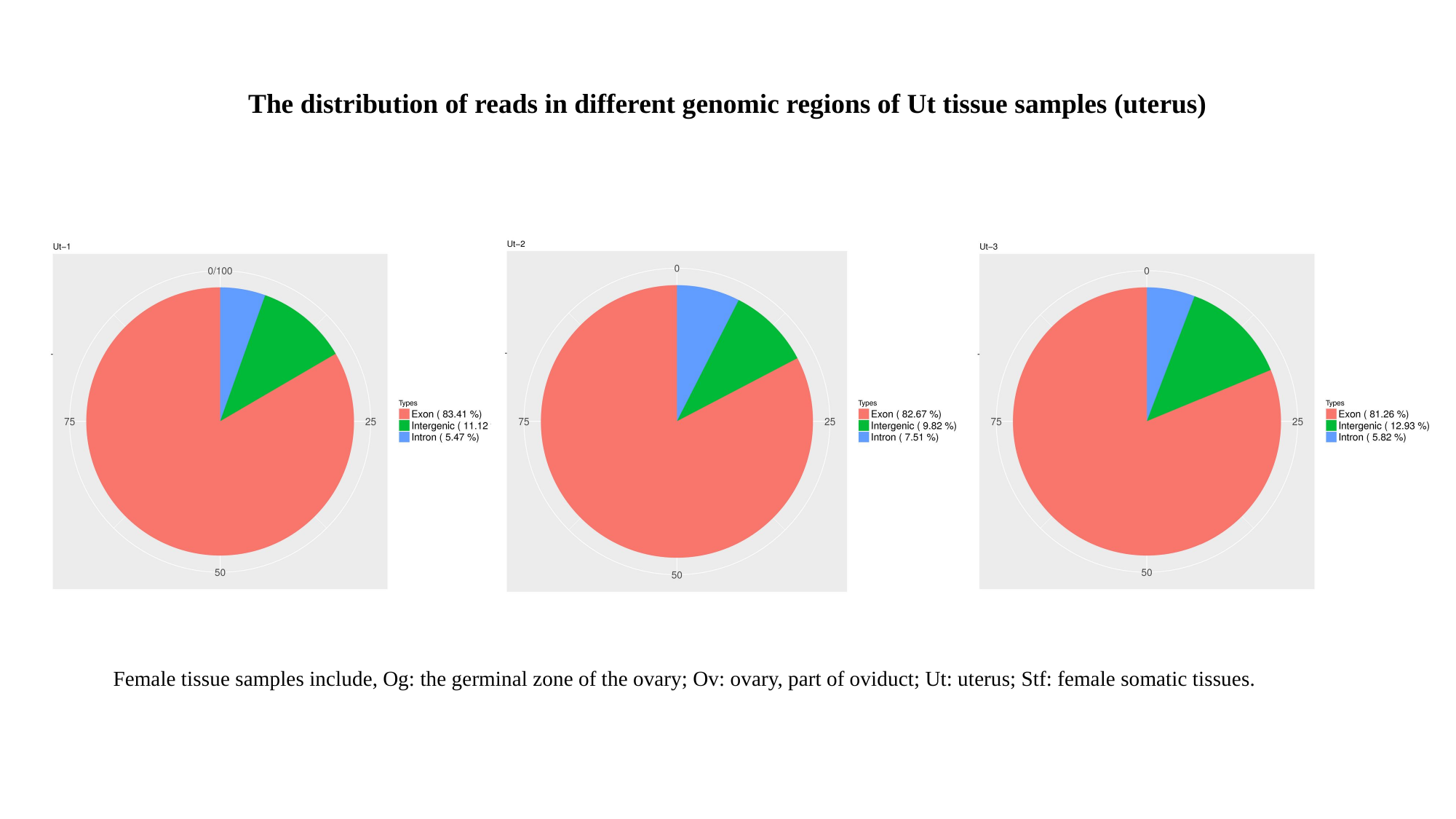

The distribution of reads in different genomic regions of Ut tissue samples (uterus)
Female tissue samples include, Og: the germinal zone of the ovary; Ov: ovary, part of oviduct; Ut: uterus; Stf: female somatic tissues.

## Slide 9
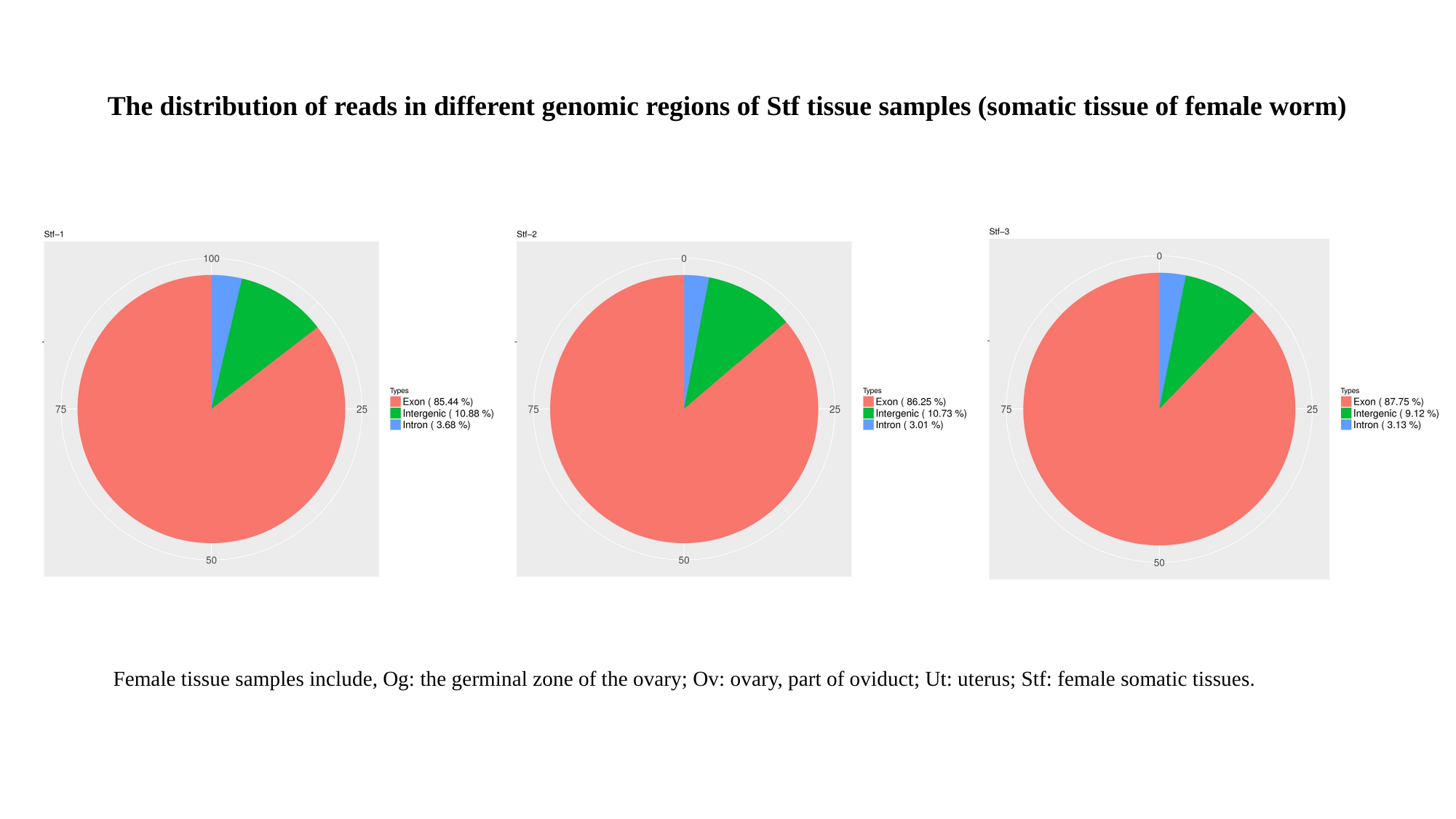

The distribution of reads in different genomic regions of Stf tissue samples (somatic tissue of female worm)
Female tissue samples include, Og: the germinal zone of the ovary; Ov: ovary, part of oviduct; Ut: uterus; Stf: female somatic tissues.
