# Supplementary material for: Transcriptome profiling of male and female Ascaris lumbricoides reproductive tissues
Source: Parasit Vectors. 2022 Dec 20;15:477. doi: 10.1186/s13071-022-05602-2 (PMC9768952; doi:10.1186/s13071-022-05602-2)
Supplement: Supplementary file 6 — Additional file 6: Figure S3. Overall quality assessment of RNA sequencing. [file 13071_2022_5602_MOESM6_ESM.pptx]

## Slide 1
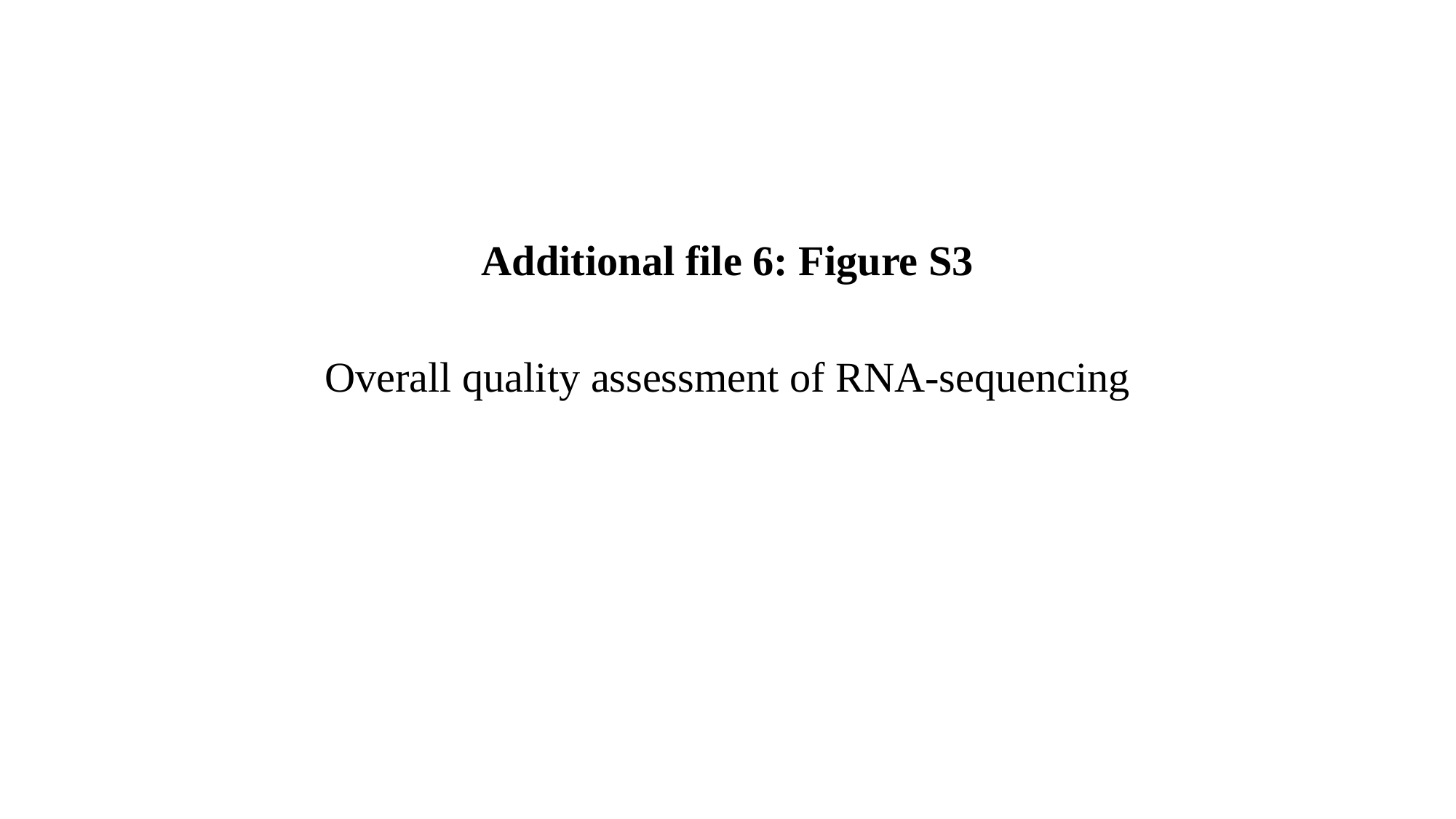

Additional file 6: Figure S3
Overall quality assessment of RNA-sequencing

## Slide 2
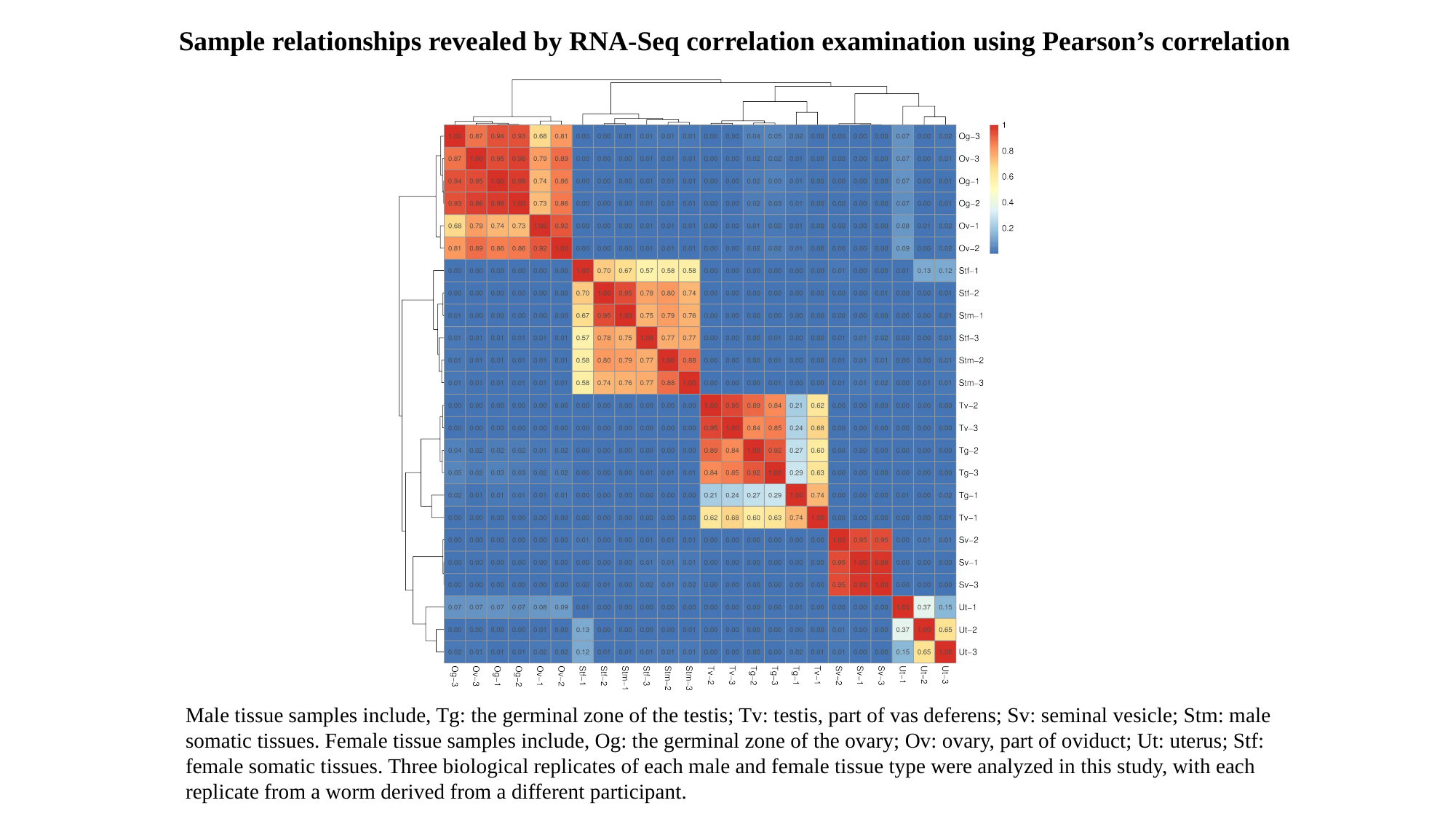

Sample relationships revealed by RNA-Seq correlation examination using Pearson’s correlation
Male tissue samples include, Tg: the germinal zone of the testis; Tv: testis, part of vas deferens; Sv: seminal vesicle; Stm: male somatic tissues. Female tissue samples include, Og: the germinal zone of the ovary; Ov: ovary, part of oviduct; Ut: uterus; Stf: female somatic tissues. Three biological replicates of each male and female tissue type were analyzed in this study, with each replicate from a worm derived from a different participant.

## Slide 3
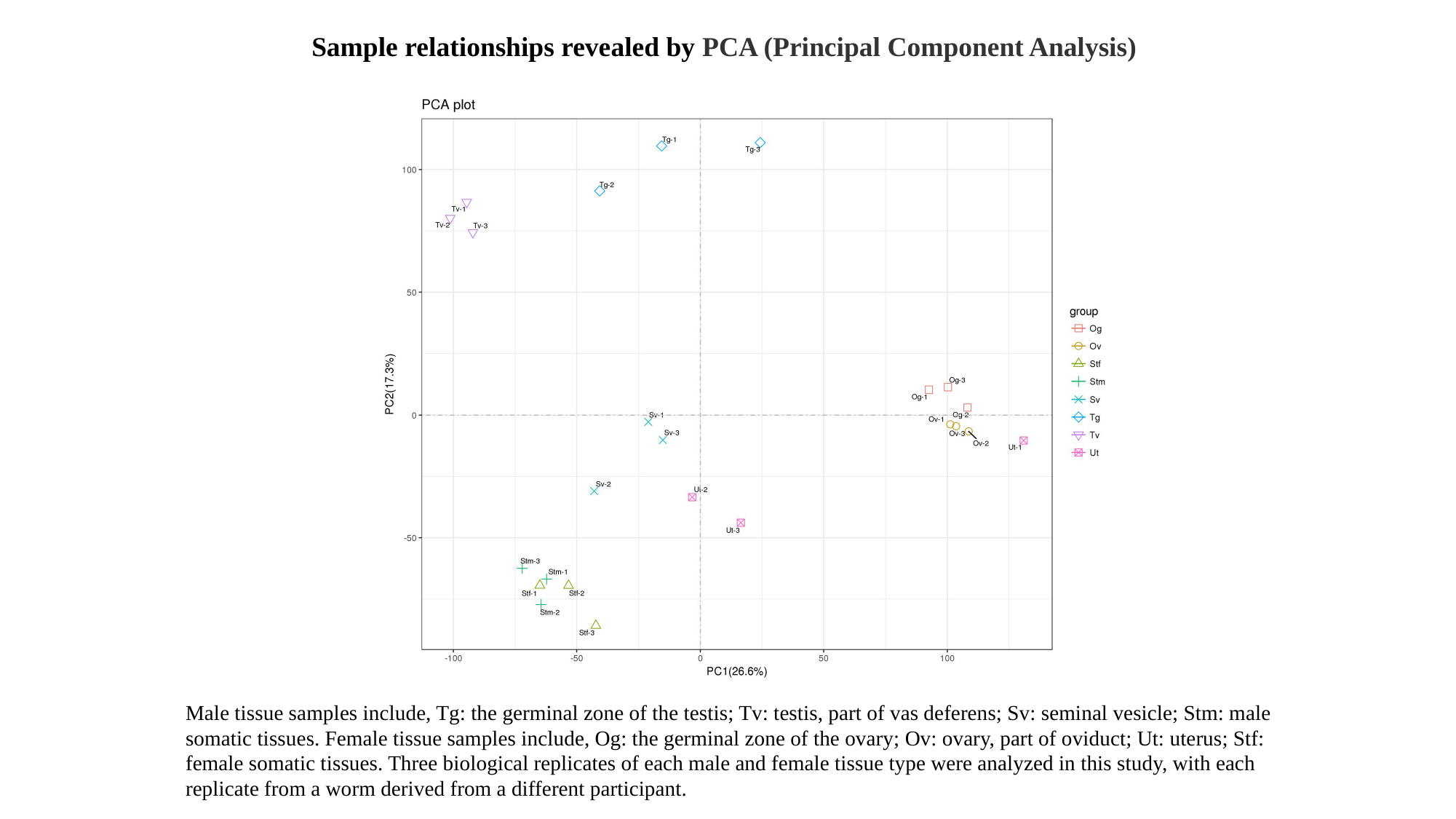

Sample relationships revealed by PCA (Principal Component Analysis)
Male tissue samples include, Tg: the germinal zone of the testis; Tv: testis, part of vas deferens; Sv: seminal vesicle; Stm: male somatic tissues. Female tissue samples include, Og: the germinal zone of the ovary; Ov: ovary, part of oviduct; Ut: uterus; Stf: female somatic tissues. Three biological replicates of each male and female tissue type were analyzed in this study, with each replicate from a worm derived from a different participant.

## Slide 4
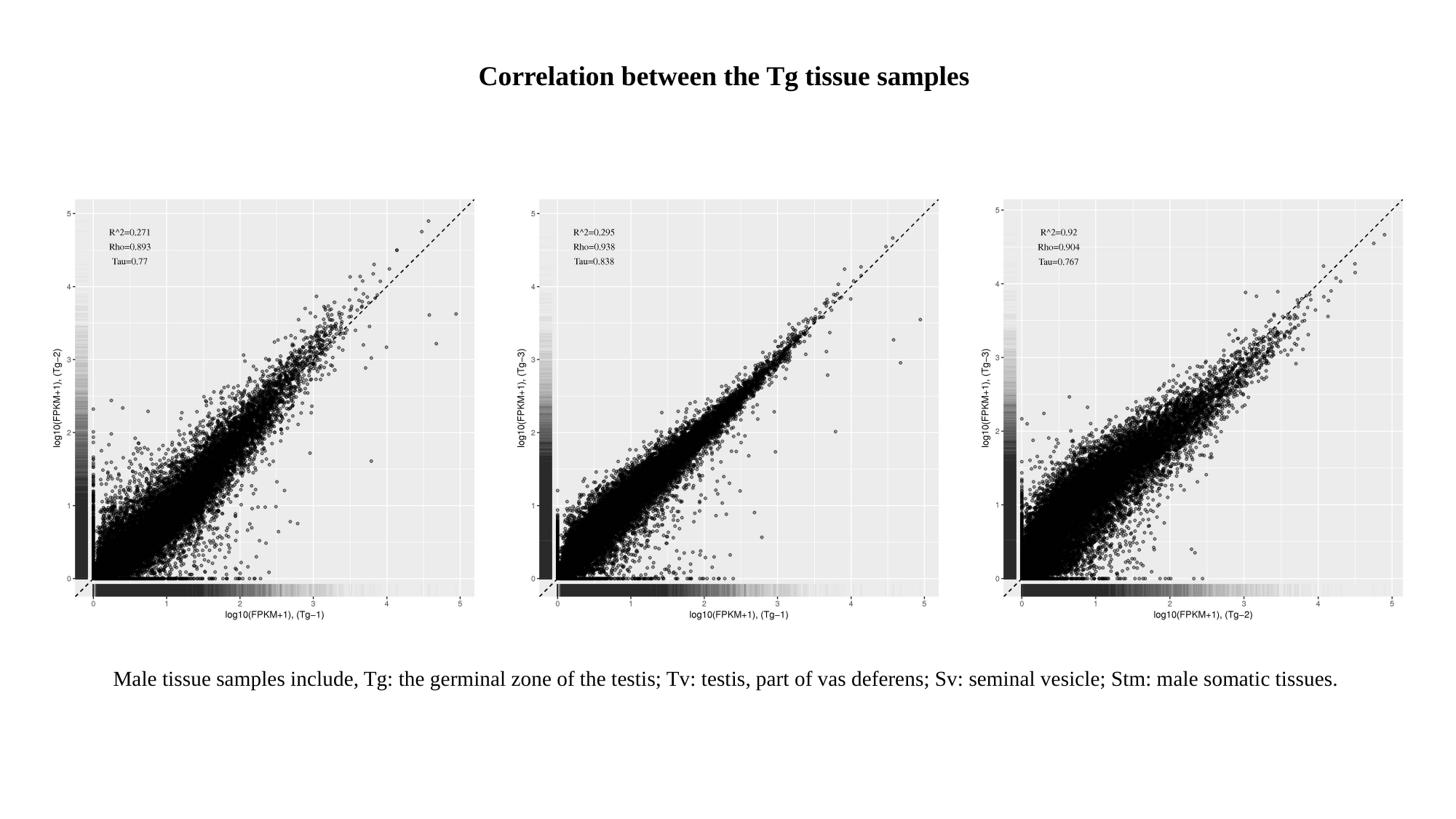

Correlation between the Tg tissue samples
Male tissue samples include, Tg: the germinal zone of the testis; Tv: testis, part of vas deferens; Sv: seminal vesicle; Stm: male somatic tissues.

## Slide 5
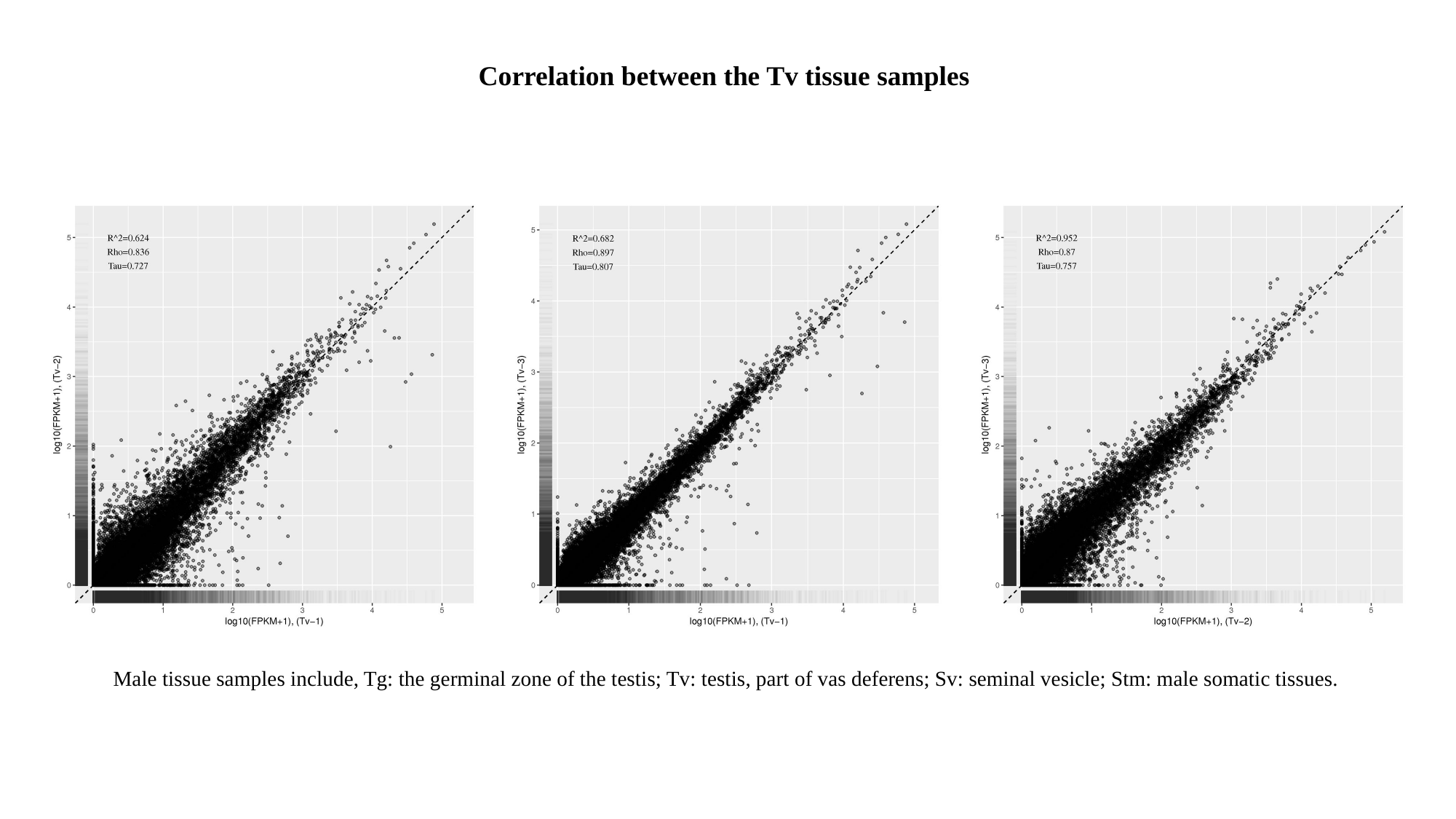

Correlation between the Tv tissue samples
Male tissue samples include, Tg: the germinal zone of the testis; Tv: testis, part of vas deferens; Sv: seminal vesicle; Stm: male somatic tissues.

## Slide 6
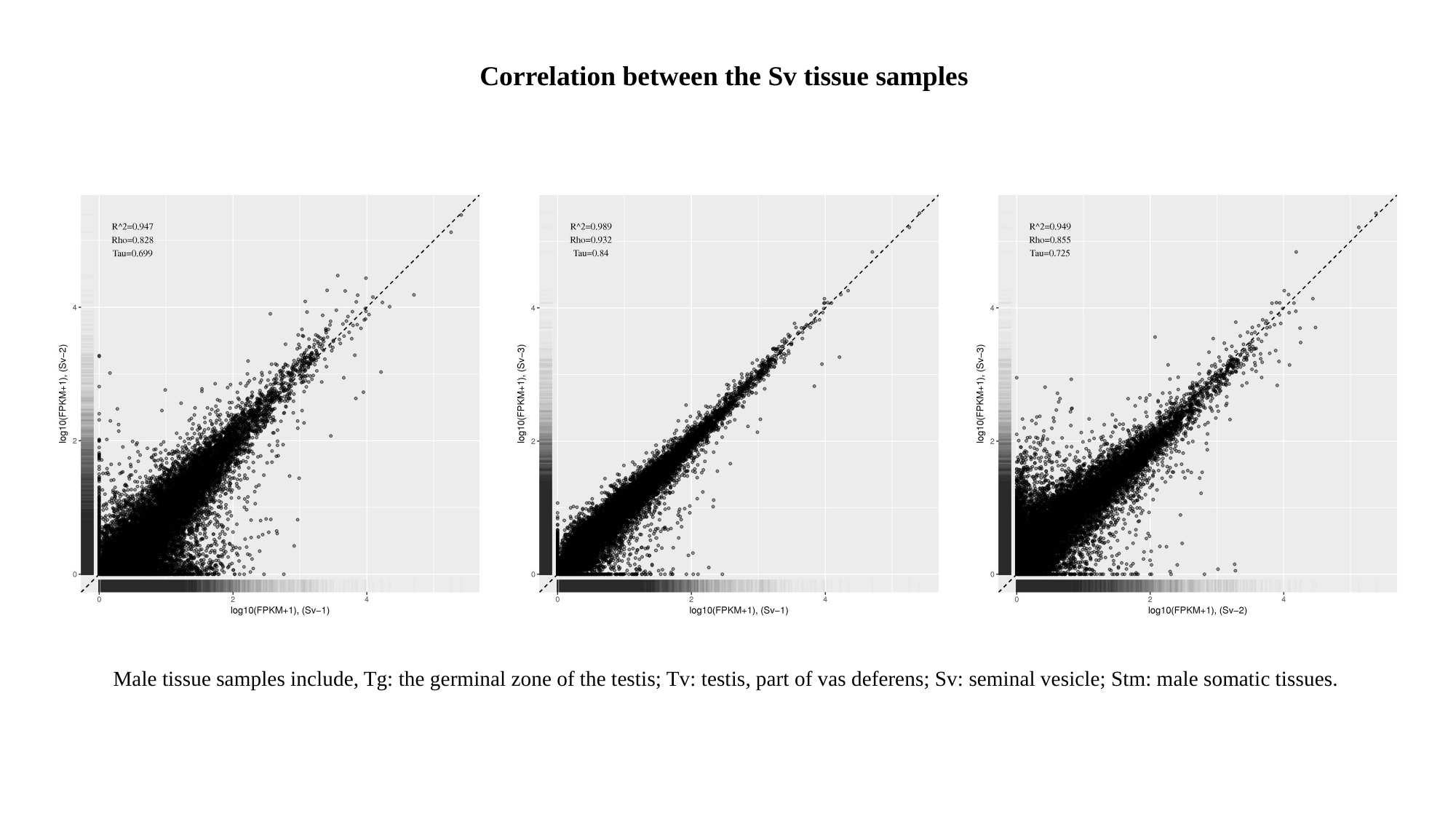

Correlation between the Sv tissue samples
Male tissue samples include, Tg: the germinal zone of the testis; Tv: testis, part of vas deferens; Sv: seminal vesicle; Stm: male somatic tissues.

## Slide 7
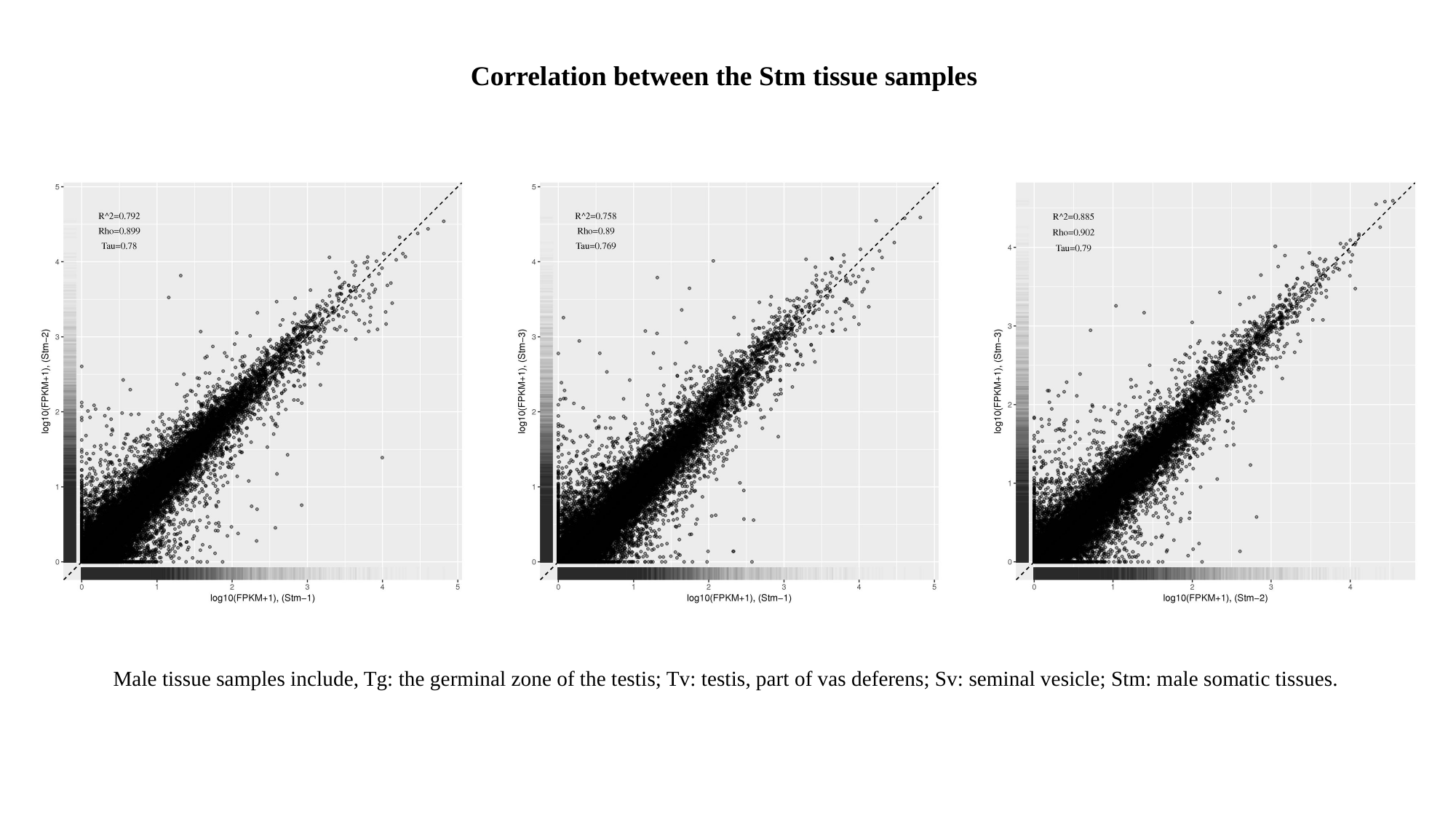

Correlation between the Stm tissue samples
Male tissue samples include, Tg: the germinal zone of the testis; Tv: testis, part of vas deferens; Sv: seminal vesicle; Stm: male somatic tissues.

## Slide 8
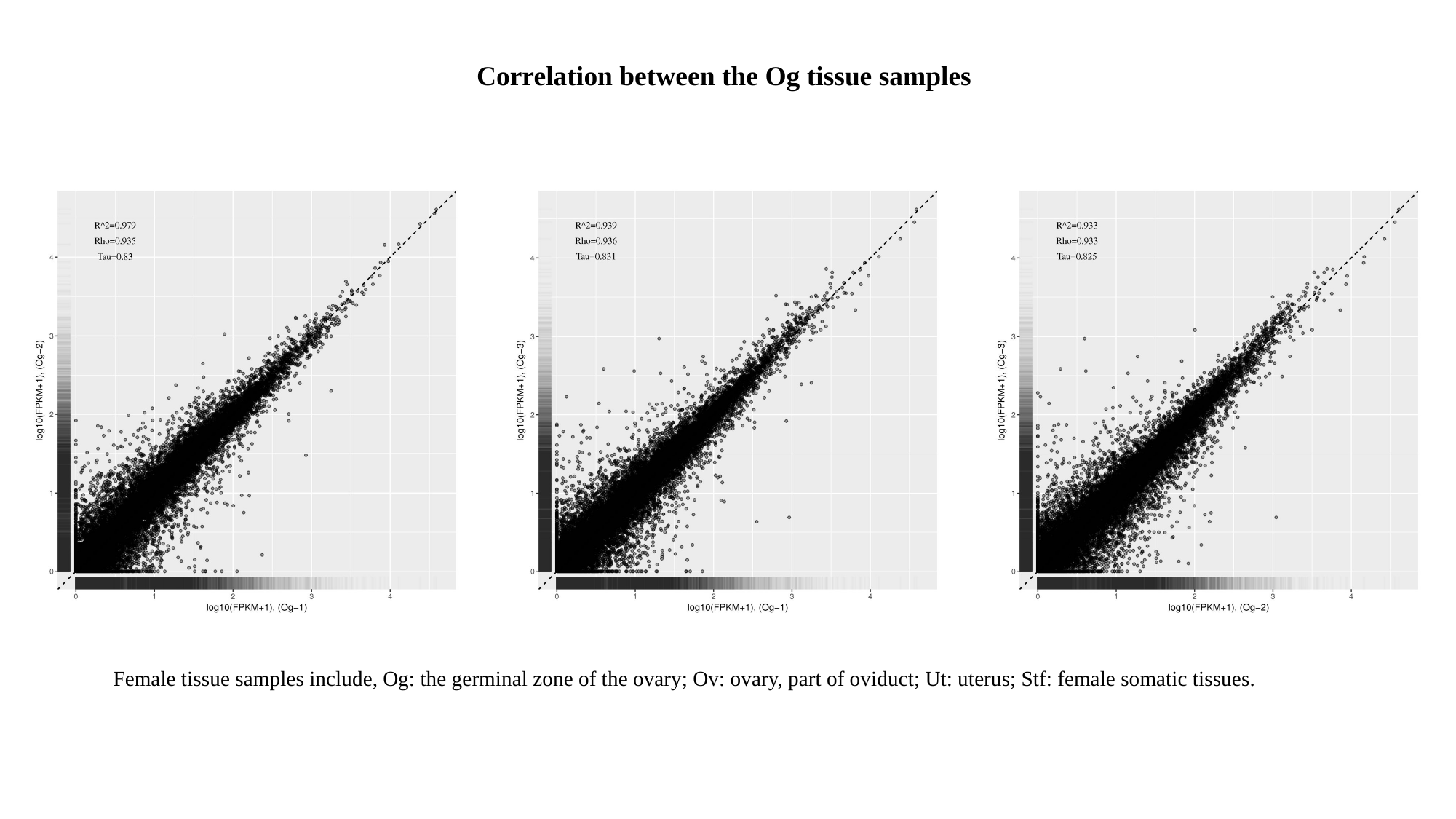

Correlation between the Og tissue samples
Female tissue samples include, Og: the germinal zone of the ovary; Ov: ovary, part of oviduct; Ut: uterus; Stf: female somatic tissues.

## Slide 9
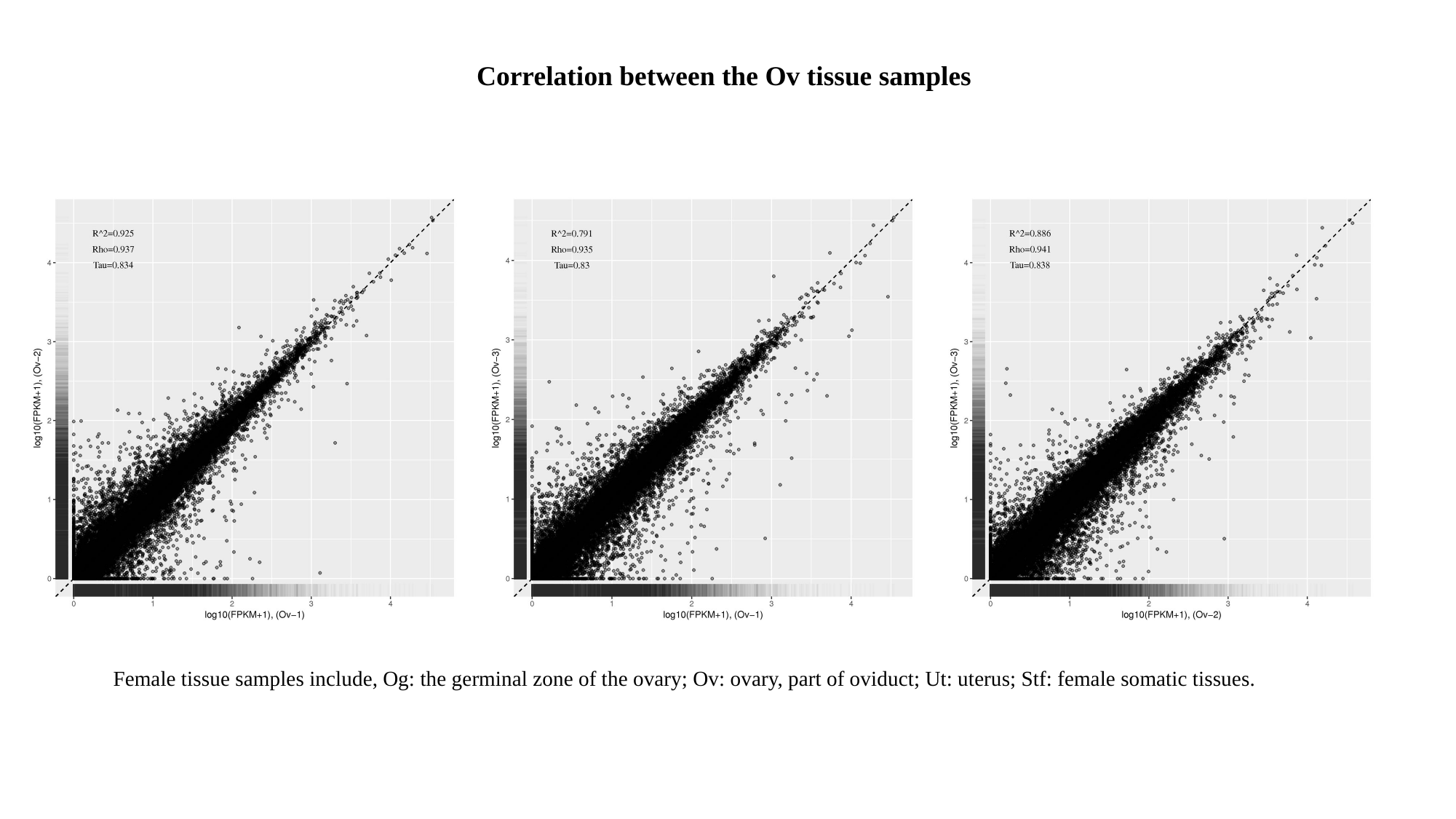

Correlation between the Ov tissue samples
Female tissue samples include, Og: the germinal zone of the ovary; Ov: ovary, part of oviduct; Ut: uterus; Stf: female somatic tissues.

## Slide 10
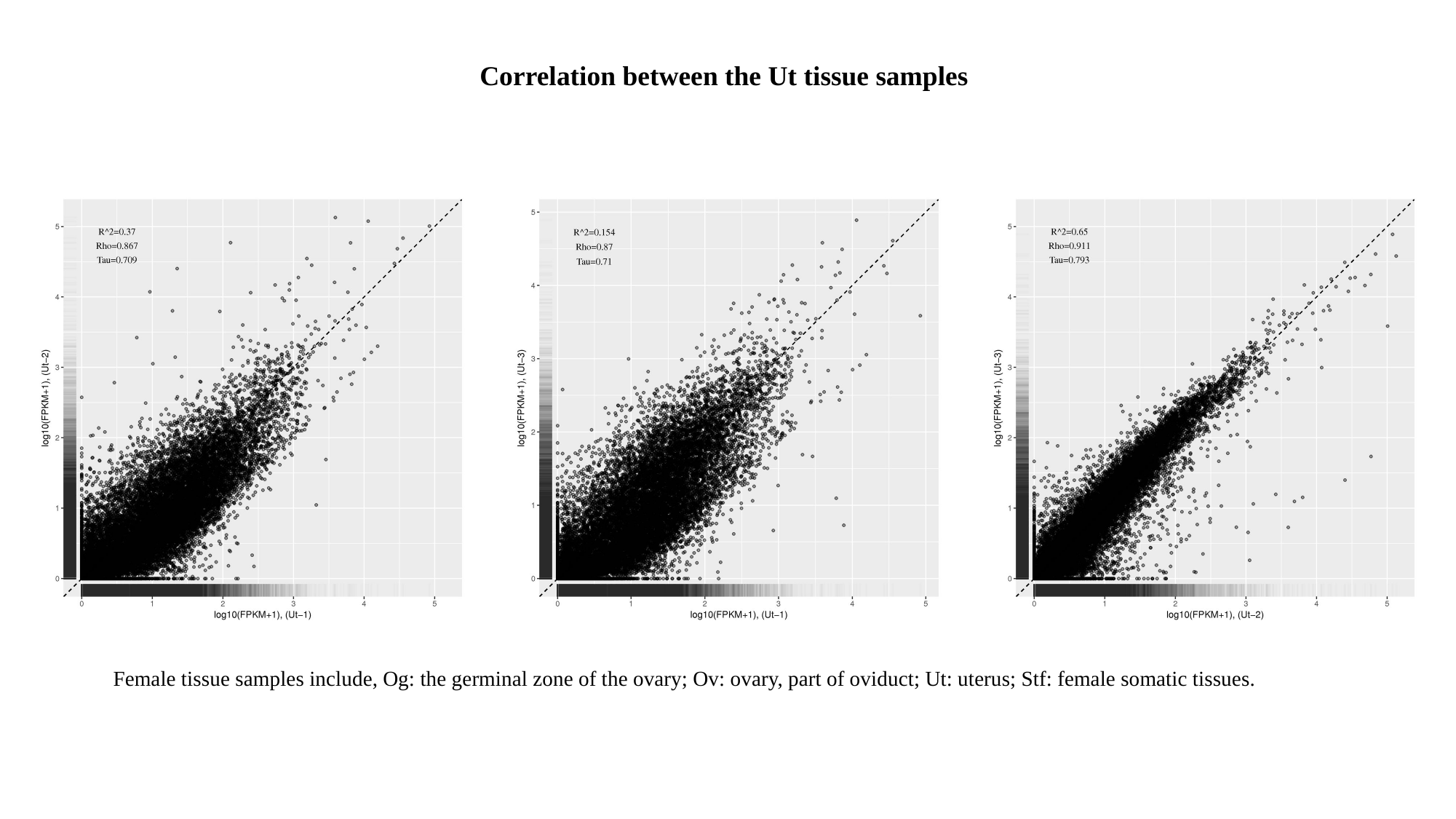

Correlation between the Ut tissue samples
Female tissue samples include, Og: the germinal zone of the ovary; Ov: ovary, part of oviduct; Ut: uterus; Stf: female somatic tissues.

## Slide 11
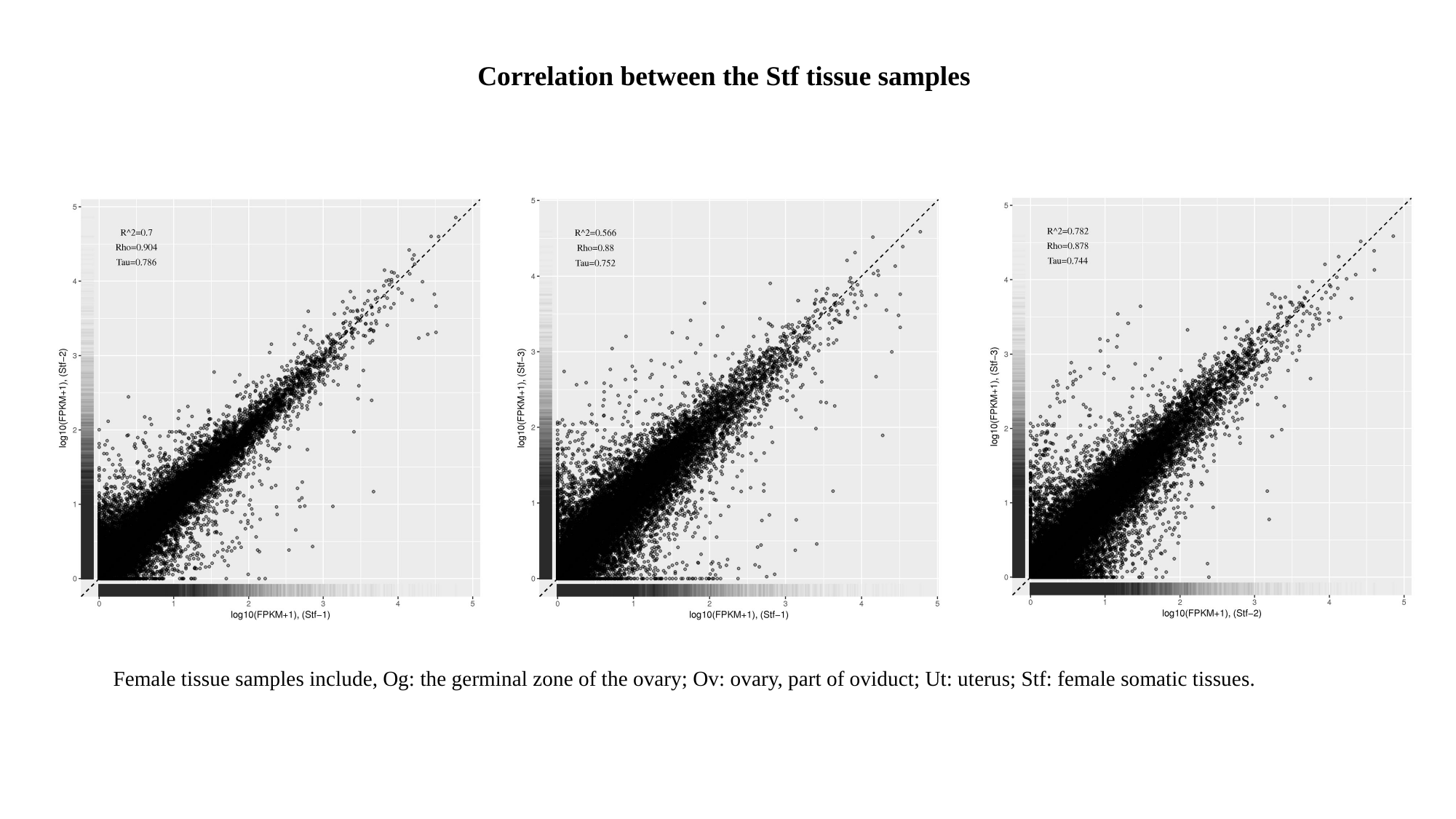

Correlation between the Stf tissue samples
Female tissue samples include, Og: the germinal zone of the ovary; Ov: ovary, part of oviduct; Ut: uterus; Stf: female somatic tissues.
