# Supplementary material for: Transcriptome profiling of male and female Ascaris lumbricoides reproductive tissues
Source: Parasit Vectors. 2022 Dec 20;15:477. doi: 10.1186/s13071-022-05602-2 (PMC9768952; doi:10.1186/s13071-022-05602-2)
Supplement: Supplementary file 11 — Additional file 11: Figure S4. Demonstration of important signaling pathways, including the Hippo signaling pathway-fly, Oxytocin signaling pathway and tight junction pathway [file 13071_2022_5602_MOESM11_ESM.pptx]

## Slide 1
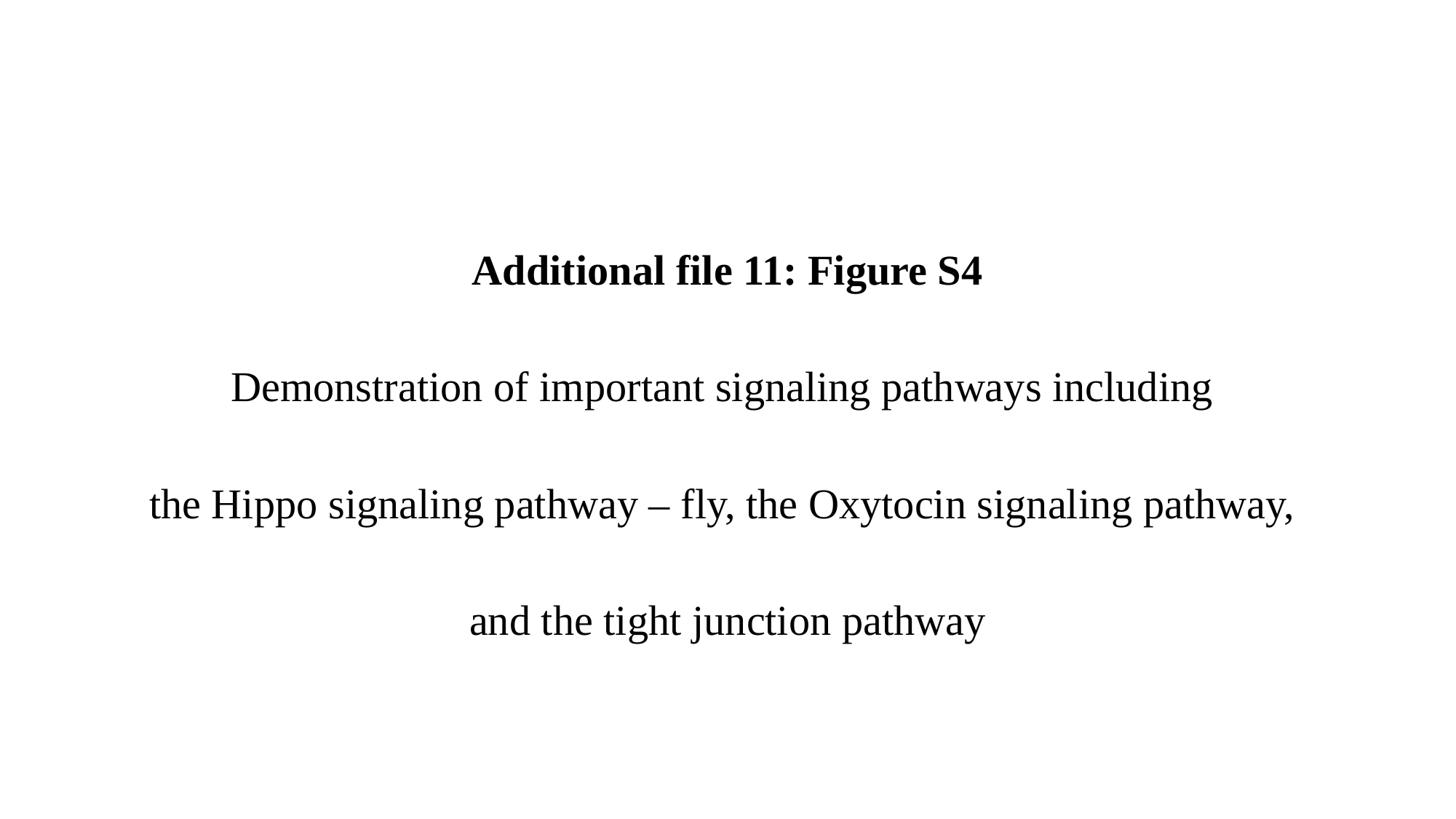

Additional file 11: Figure S4
Demonstration of important signaling pathways including
the Hippo signaling pathway – fly, the Oxytocin signaling pathway,
and the tight junction pathway

## Slide 2
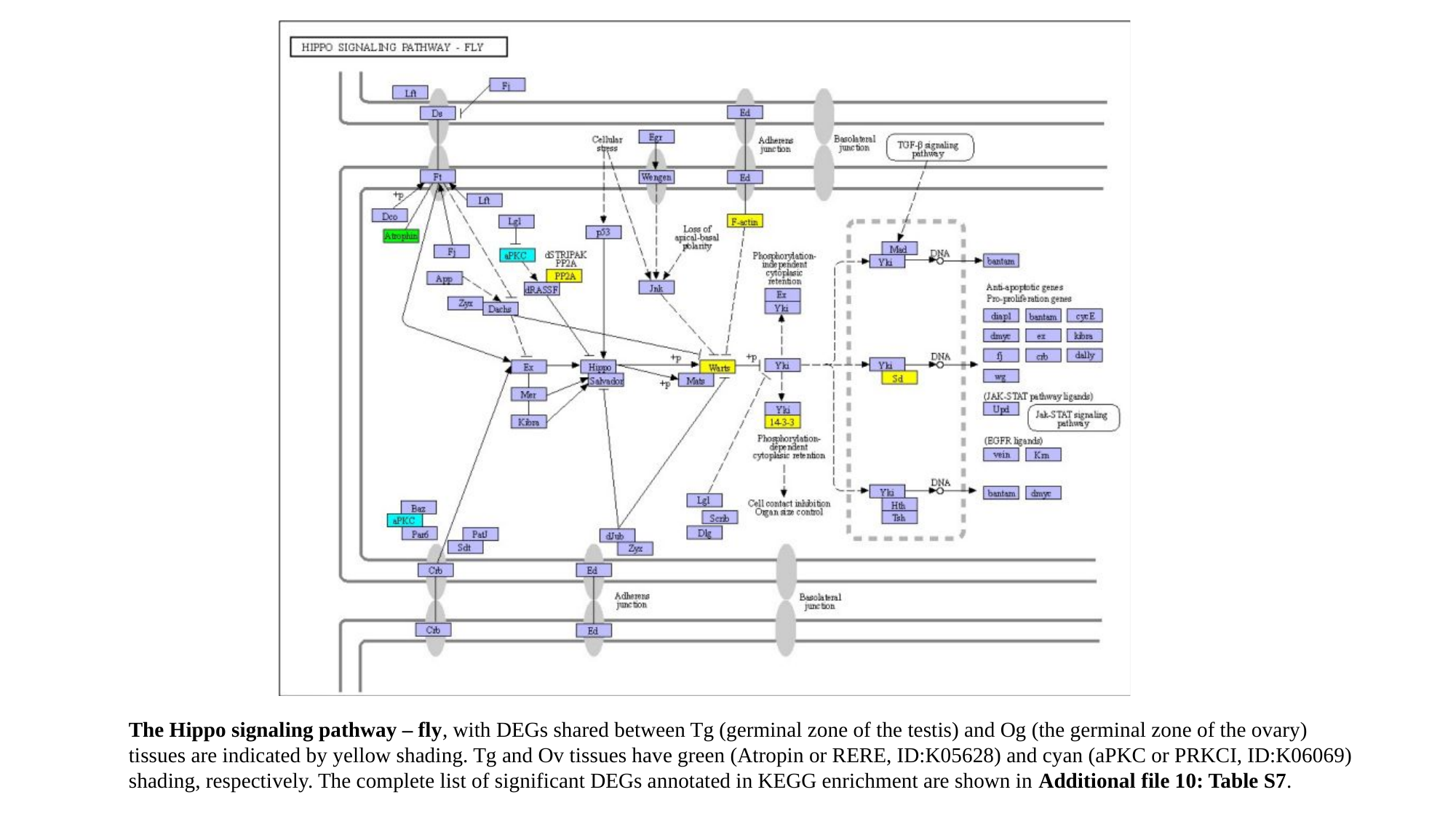

The Hippo signaling pathway – fly, with DEGs shared between Tg (germinal zone of the testis) and Og (the germinal zone of the ovary) tissues are indicated by yellow shading. Tg and Ov tissues have green (Atropin or RERE, ID:K05628) and cyan (aPKC or PRKCI, ID:K06069) shading, respectively. The complete list of significant DEGs annotated in KEGG enrichment are shown in Additional file 10: Table S7.

## Slide 3
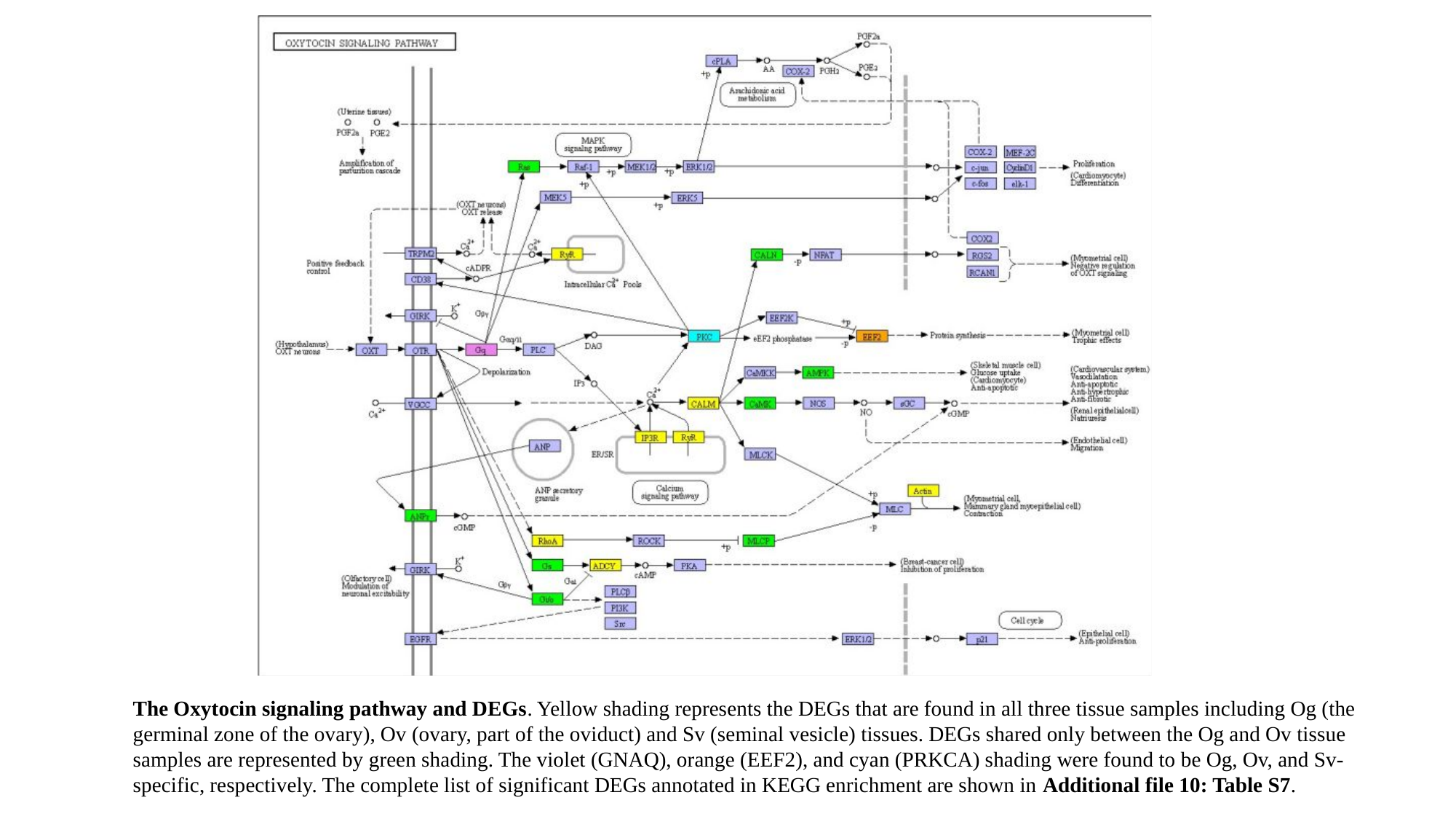

The Oxytocin signaling pathway and DEGs. Yellow shading represents the DEGs that are found in all three tissue samples including Og (the germinal zone of the ovary), Ov (ovary, part of the oviduct) and Sv (seminal vesicle) tissues. DEGs shared only between the Og and Ov tissue samples are represented by green shading. The violet (GNAQ), orange (EEF2), and cyan (PRKCA) shading were found to be Og, Ov, and Sv-specific, respectively. The complete list of significant DEGs annotated in KEGG enrichment are shown in Additional file 10: Table S7.

## Slide 4
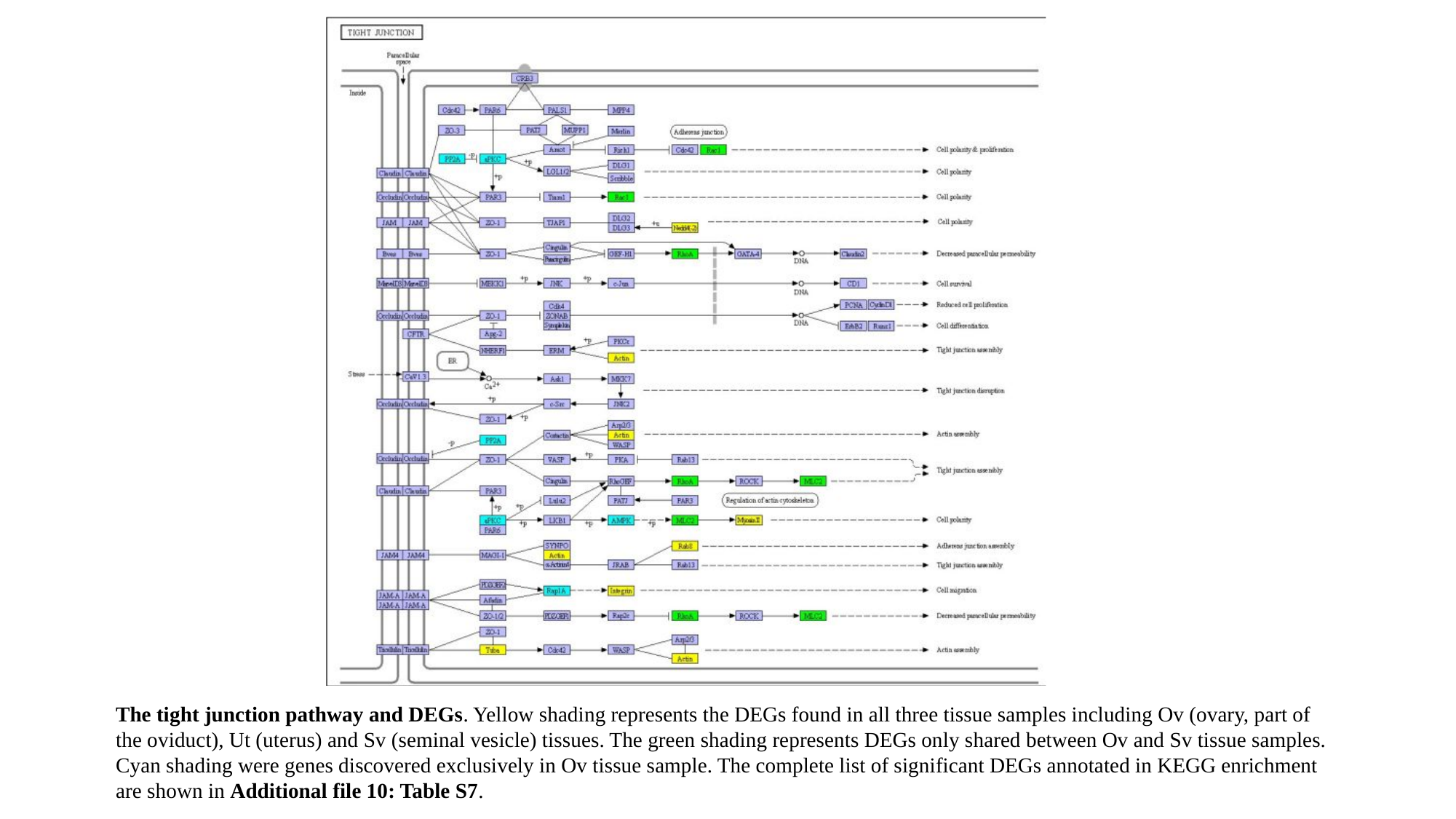

The tight junction pathway and DEGs. Yellow shading represents the DEGs found in all three tissue samples including Ov (ovary, part of the oviduct), Ut (uterus) and Sv (seminal vesicle) tissues. The green shading represents DEGs only shared between Ov and Sv tissue samples. Cyan shading were genes discovered exclusively in Ov tissue sample. The complete list of significant DEGs annotated in KEGG enrichment are shown in Additional file 10: Table S7.
