# Supplementary material for: Transcriptome profiling of male and female Ascaris lumbricoides reproductive tissues
Source: Parasit Vectors. 2022 Dec 20;15:477. doi: 10.1186/s13071-022-05602-2 (PMC9768952; doi:10.1186/s13071-022-05602-2)
Supplement: Supplementary file 13 — Additional file 13: Figure S5. Gene expression level of tissue-specific genes in A. lumbricoides discrete tissues [file 13071_2022_5602_MOESM13_ESM.pptx]

## Slide 1
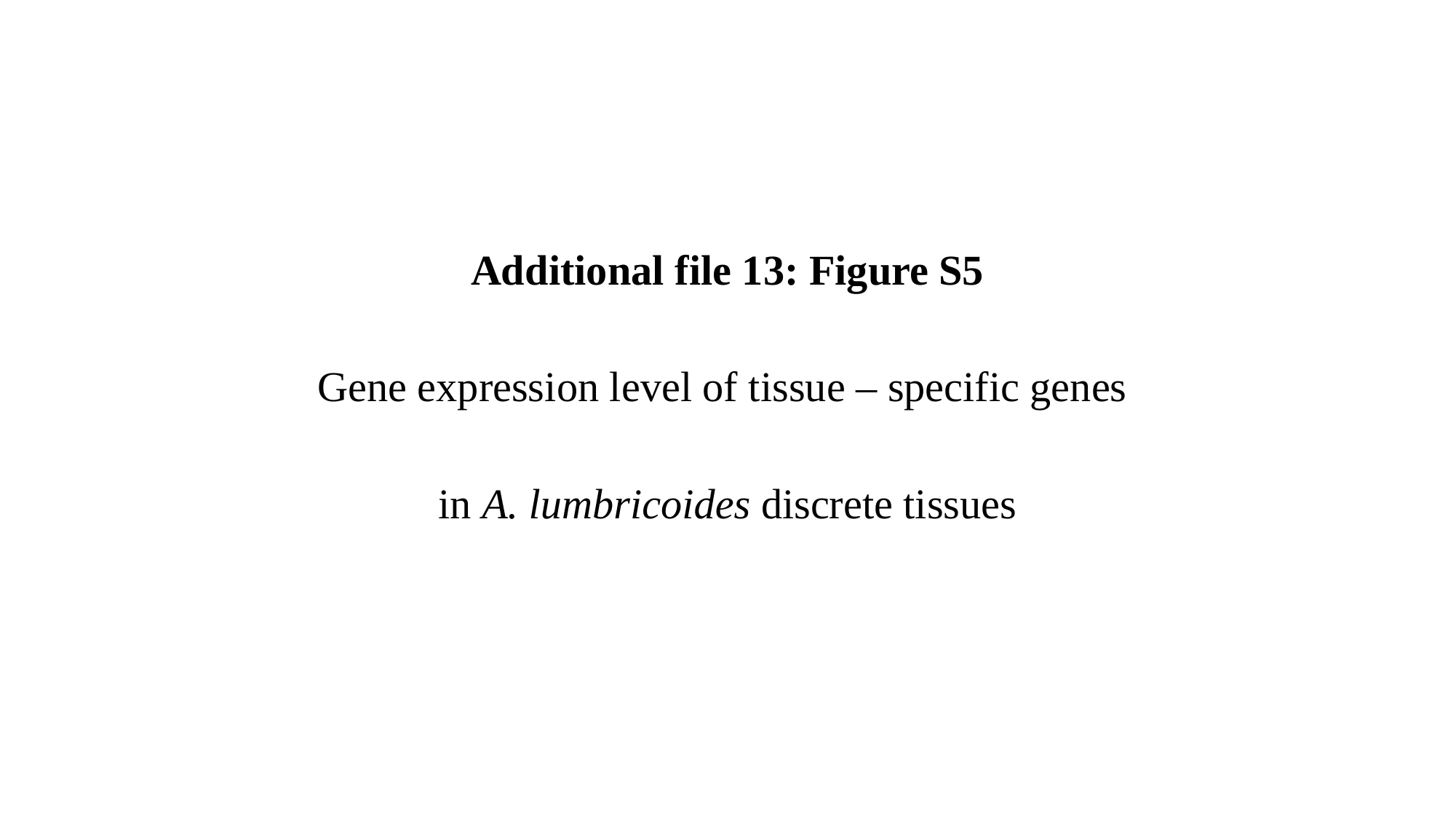

Additional file 13: Figure S5
Gene expression level of tissue – specific genes
in A. lumbricoides discrete tissues

## Slide 2
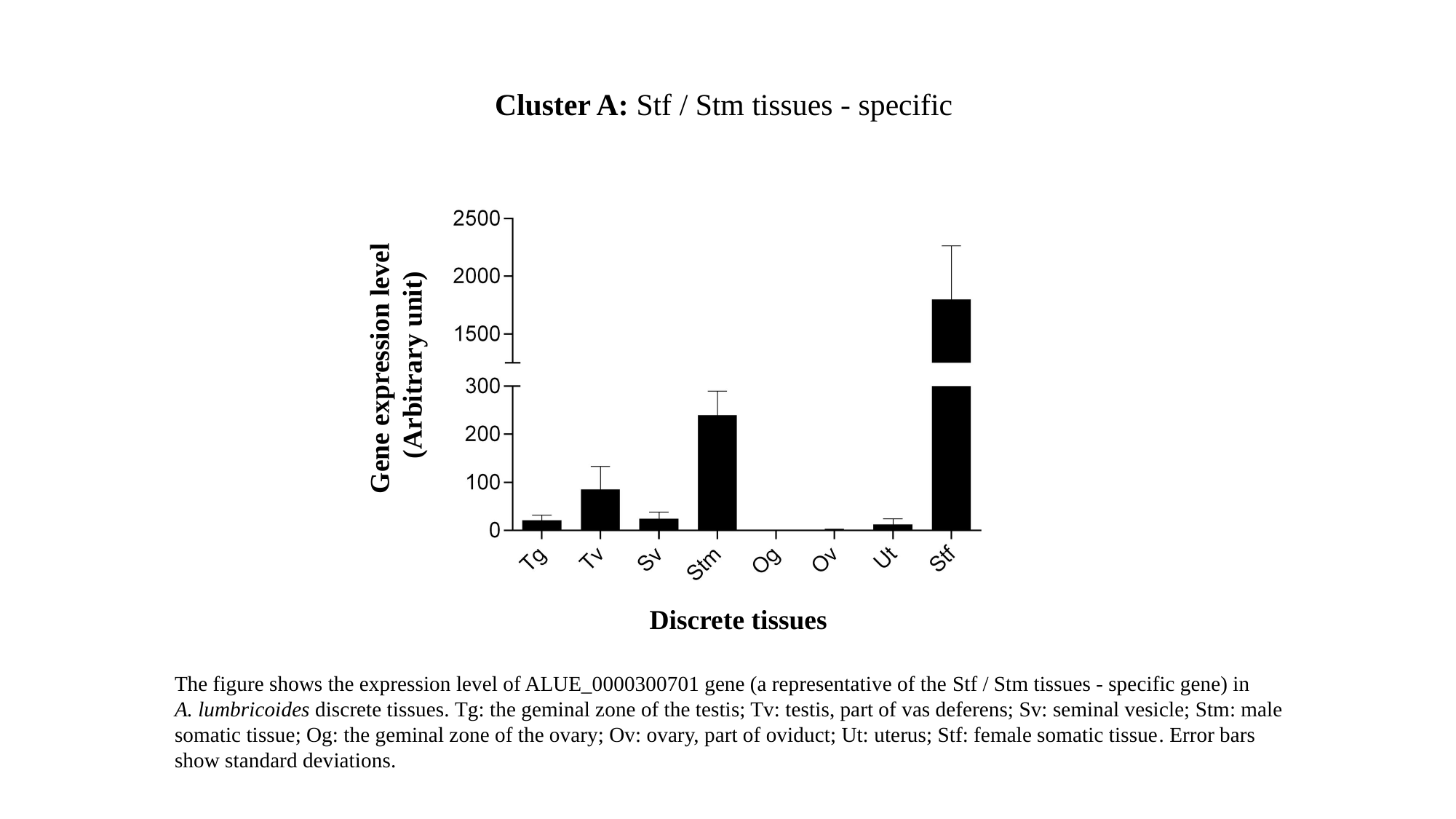

Cluster A: Stf / Stm tissues - specific
Gene expression level
(Arbitrary unit)
Discrete tissues
The figure shows the expression level of ALUE_0000300701 gene (a representative of the Stf / Stm tissues - specific gene) in
A. lumbricoides discrete tissues. Tg: the geminal zone of the testis; Tv: testis, part of vas deferens; Sv: seminal vesicle; Stm: male somatic tissue; Og: the geminal zone of the ovary; Ov: ovary, part of oviduct; Ut: uterus; Stf: female somatic tissue. Error bars show standard deviations.

## Slide 3
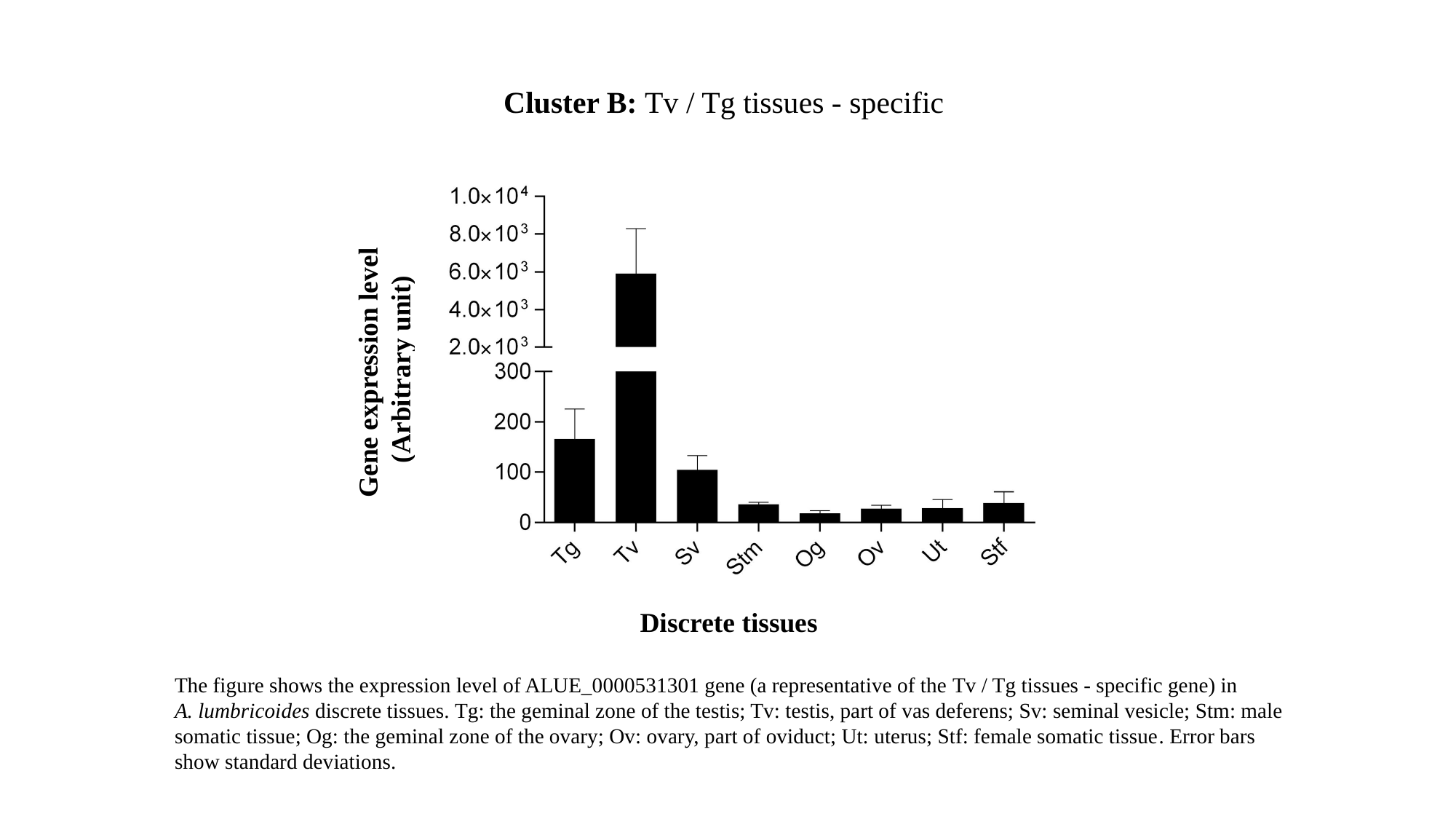

Cluster B: Tv / Tg tissues - specific
Gene expression level
(Arbitrary unit)
Discrete tissues
The figure shows the expression level of ALUE_0000531301 gene (a representative of the Tv / Tg tissues - specific gene) in
A. lumbricoides discrete tissues. Tg: the geminal zone of the testis; Tv: testis, part of vas deferens; Sv: seminal vesicle; Stm: male somatic tissue; Og: the geminal zone of the ovary; Ov: ovary, part of oviduct; Ut: uterus; Stf: female somatic tissue. Error bars show standard deviations.

## Slide 4
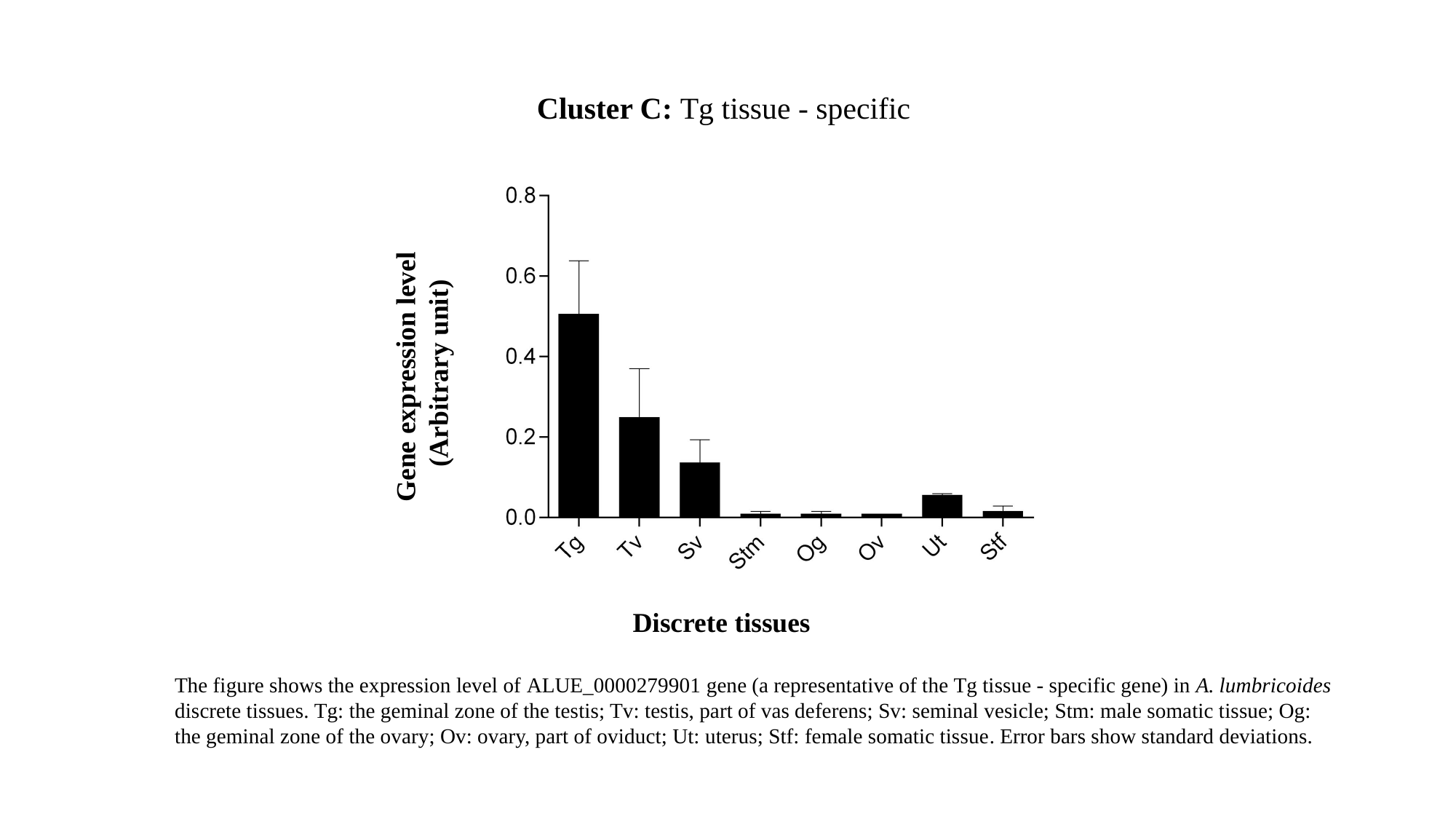

Cluster C: Tg tissue - specific
Gene expression level
(Arbitrary unit)
Discrete tissues
The figure shows the expression level of ALUE_0000279901 gene (a representative of the Tg tissue - specific gene) in A. lumbricoides discrete tissues. Tg: the geminal zone of the testis; Tv: testis, part of vas deferens; Sv: seminal vesicle; Stm: male somatic tissue; Og: the geminal zone of the ovary; Ov: ovary, part of oviduct; Ut: uterus; Stf: female somatic tissue. Error bars show standard deviations.

## Slide 5
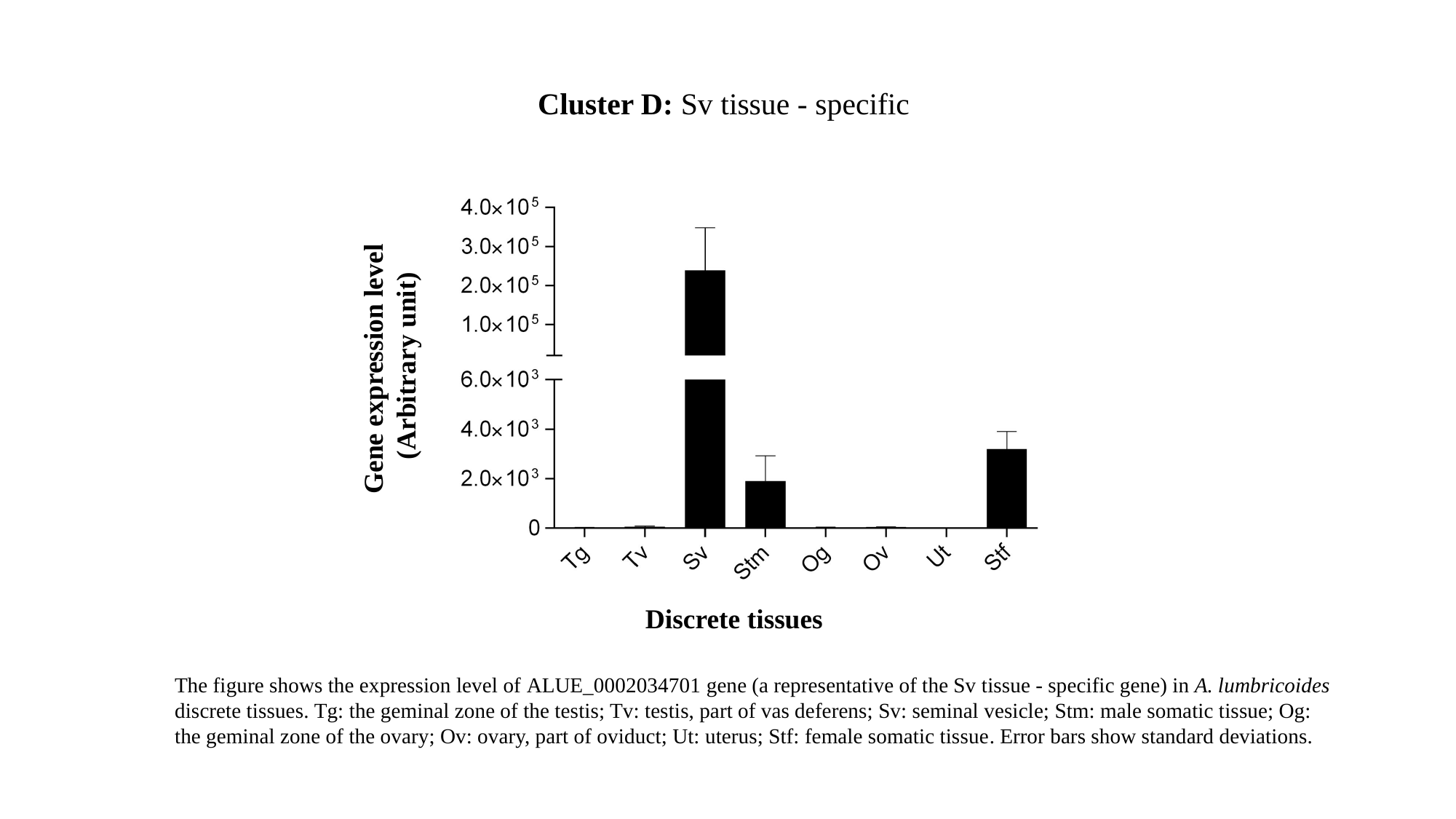

Cluster D: Sv tissue - specific
Gene expression level
(Arbitrary unit)
Discrete tissues
The figure shows the expression level of ALUE_0002034701 gene (a representative of the Sv tissue - specific gene) in A. lumbricoides discrete tissues. Tg: the geminal zone of the testis; Tv: testis, part of vas deferens; Sv: seminal vesicle; Stm: male somatic tissue; Og: the geminal zone of the ovary; Ov: ovary, part of oviduct; Ut: uterus; Stf: female somatic tissue. Error bars show standard deviations.

## Slide 6
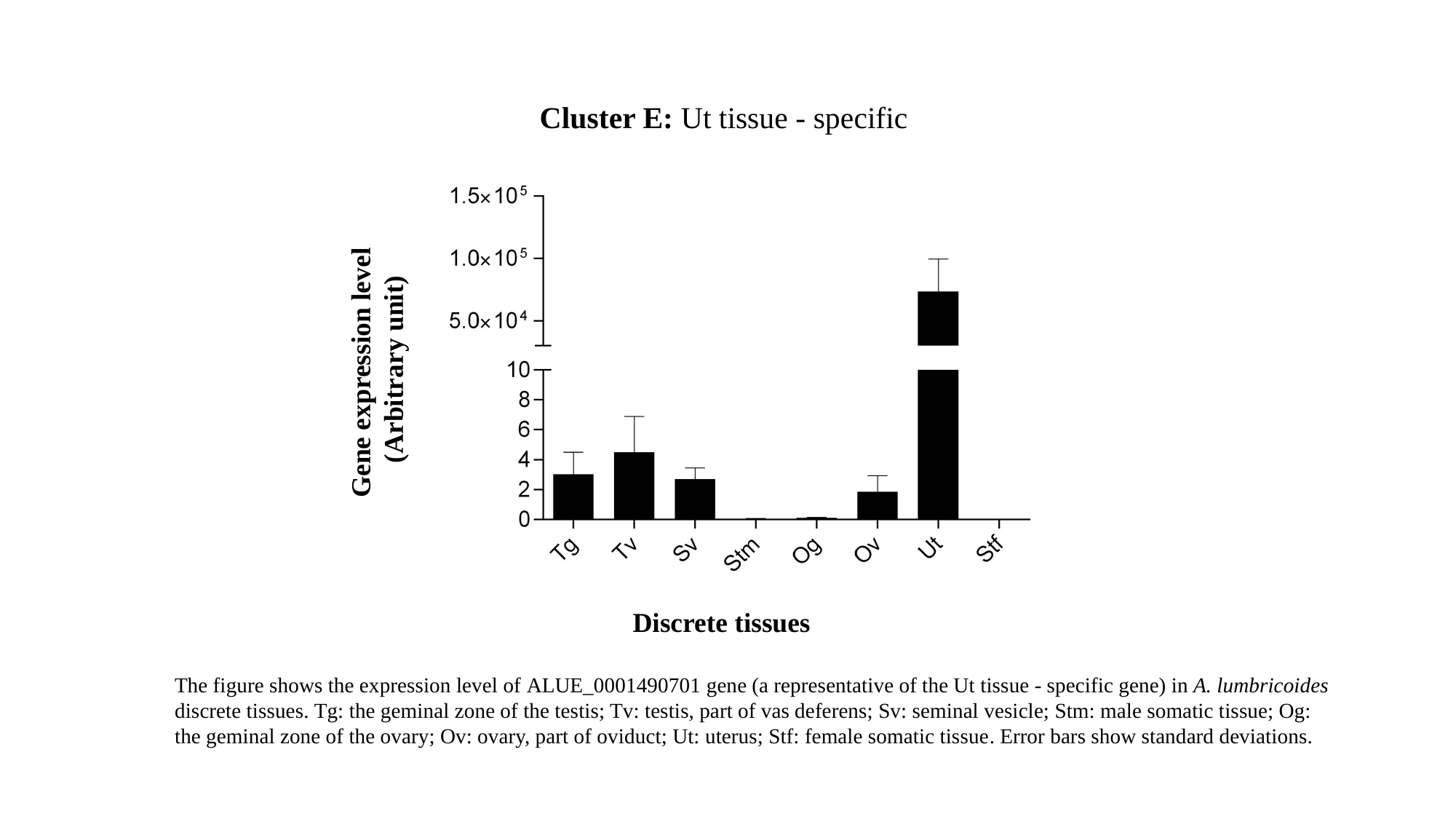

Cluster E: Ut tissue - specific
Gene expression level
(Arbitrary unit)
Discrete tissues
The figure shows the expression level of ALUE_0001490701 gene (a representative of the Ut tissue - specific gene) in A. lumbricoides discrete tissues. Tg: the geminal zone of the testis; Tv: testis, part of vas deferens; Sv: seminal vesicle; Stm: male somatic tissue; Og: the geminal zone of the ovary; Ov: ovary, part of oviduct; Ut: uterus; Stf: female somatic tissue. Error bars show standard deviations.

## Slide 7
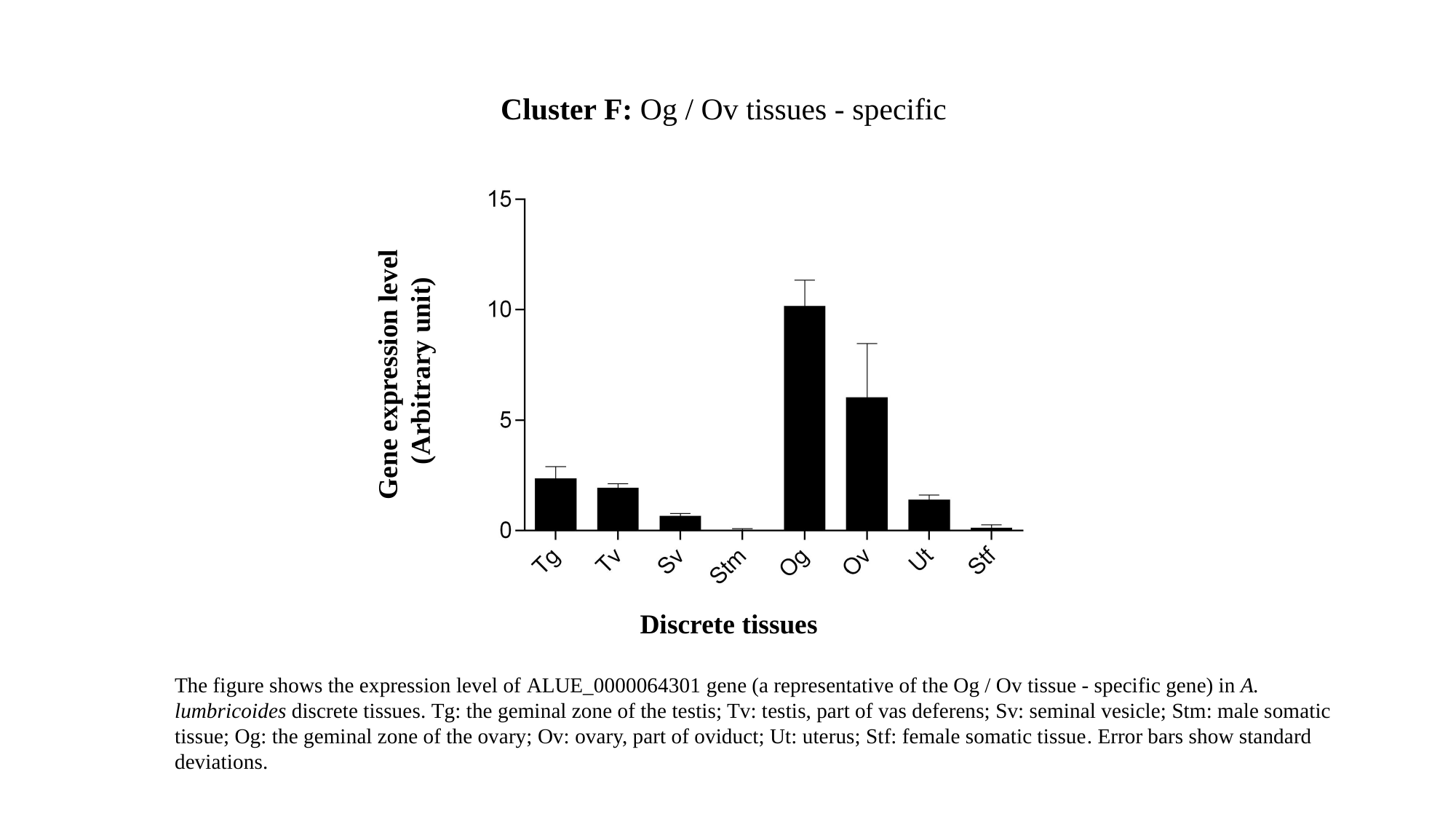

Cluster F: Og / Ov tissues - specific
Gene expression level
(Arbitrary unit)
Discrete tissues
The figure shows the expression level of ALUE_0000064301 gene (a representative of the Og / Ov tissue - specific gene) in A. lumbricoides discrete tissues. Tg: the geminal zone of the testis; Tv: testis, part of vas deferens; Sv: seminal vesicle; Stm: male somatic tissue; Og: the geminal zone of the ovary; Ov: ovary, part of oviduct; Ut: uterus; Stf: female somatic tissue. Error bars show standard deviations.

## Slide 8
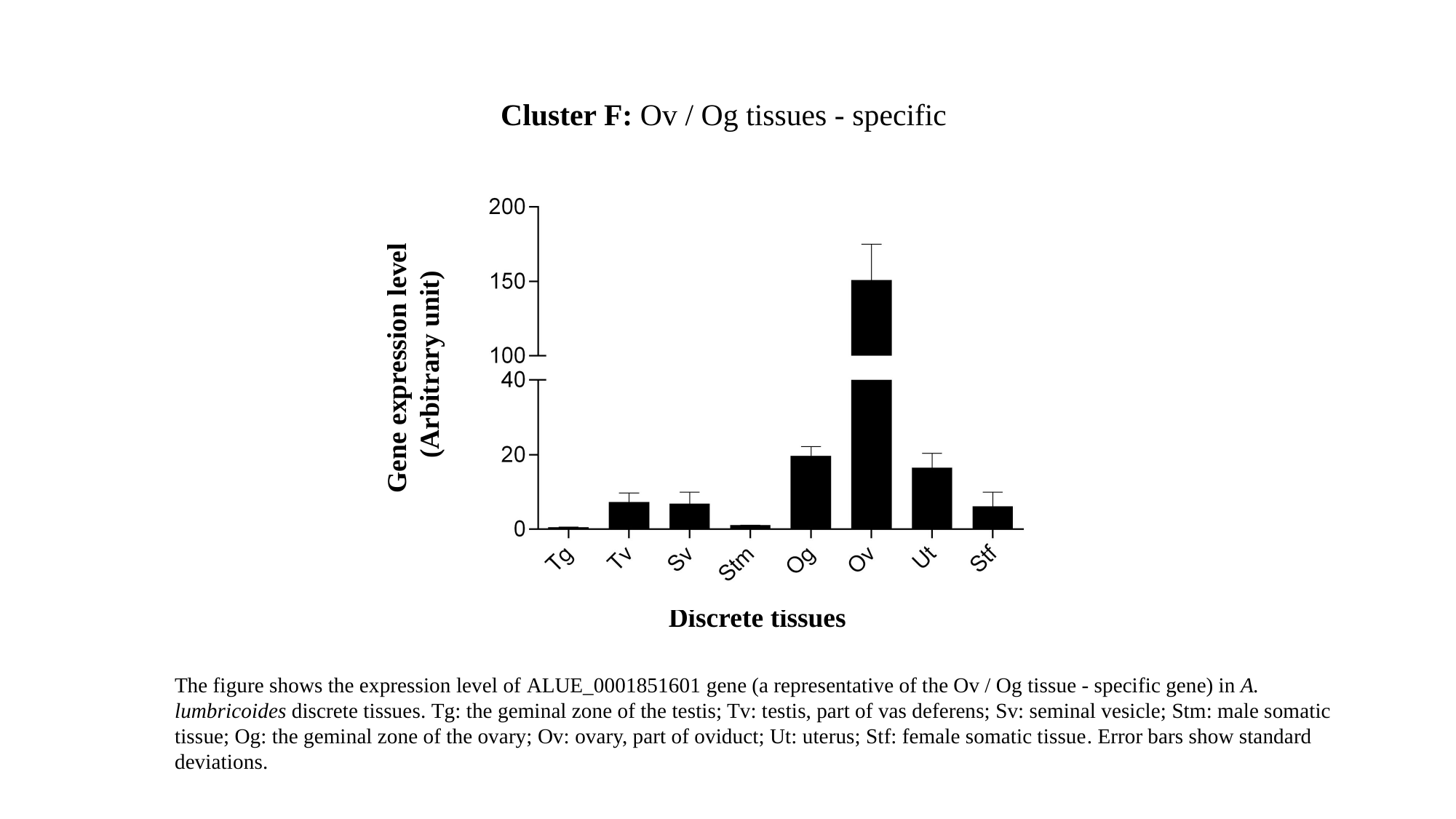

Cluster F: Ov / Og tissues - specific
Gene expression level
(Arbitrary unit)
Discrete tissues
The figure shows the expression level of ALUE_0001851601 gene (a representative of the Ov / Og tissue - specific gene) in A. lumbricoides discrete tissues. Tg: the geminal zone of the testis; Tv: testis, part of vas deferens; Sv: seminal vesicle; Stm: male somatic tissue; Og: the geminal zone of the ovary; Ov: ovary, part of oviduct; Ut: uterus; Stf: female somatic tissue. Error bars show standard deviations.
